# Genetic evolution of influenza H9N2 viruses isolated from various hosts in China from 1994 to 2013

**DOI:** 10.1038/emi.2017.94

**Published:** 2017-11-29

**Authors:** Chong Li, Shuoguo Wang, Guoxia Bing, Robert A Carter, Zejiang Wang, Jinliang Wang, Chenxi Wang, Lan Wang, Gang Wu, Robert G Webster, Yongqiang Wang, Honglei Sun, Yipeng Sun, Jinhua Liu, Juan Pu

**Affiliations:** 1 Key Laboratory of Animal Epidemiology, Ministry of Agriculture, College of Veterinary Medicine and State Key Laboratory of Agrobiotechnology, China Agricultural University, Beijing 100193, China; 2 Department of Infectious Diseases, St Jude Children’s Research Hospital, Memphis, TN 38105, USA; 3 China Animal Disease Control Center, Beijing 100125, China

**Keywords:** genetic evolution, genotype, geographic distribution, host range, H9N2 influenza virus

## Abstract

Influenza H9N2 subtype viruses and their reassortants (such as H7N9) are posing increasing threats to birds and humans in China. During 2009–2013, multiple novel subtype viruses with H9N2 original genes emerged in China. Yet, the genetic evolution of H9N2 viruses in various host organisms in China has not been systematically investigated since 2009. In the present study, we performed large-scale sequence analysis of H9N2 viral genomes from public databases, representing the spectrum of viruses isolated from birds, mammals and humans in China from 1994 to 2013, and updated the clade classification for each segment. We identified 117 distinct genotypes in 730 H9N2 viruses. We analyzed the sequences of all eight segments in each virus and found three important time points: the years 2000, 2006 and 2010. In the periods divided by these years, genotypic diversity, geographic distribution and host range changed considerably. Genotypic diversity fluctuated greatly in 2000 and 2006. Since 2010, a single genotype became predominant in poultry throughout China, and the eastern coastal region became the newly identified epidemic center. Throughout their 20-year prevalence in China, H9N2 influenza viruses have emerged and adapted from aquatic birds to chickens. The minor avian species and wild birds exacerbated H9N2 genotypes by providing diversified genes, and chickens were the most prevalent vector in which the viruses evolved and expanded their prevalence. It is the necessity for surveillance and disease control on live-bird markets, poultry farms and wild-bird habitats in China.

## Introduction

H9N2 subtype influenza viruses have become endemic in various types of terrestrial poultry in Eurasian and African countries and have caused sporadic infections in humans and mammals.^1, 2^ China is regarded as the epidemic center of H9N2 viruses.^3^ However, unlike highly pathogenic H5N1 avian influenza viruses, low pathogenicity H9N2 viruses have attracted less attention in disease management and public health control.^4^ During 2009–2013, multiple novel subtype viruses with H9N2 original genes emerged in China.^5^ In 2013, a novel reassortant H7N9 virus containing six internal genes from H9N2 viruses caused serious outbreaks in humans in China,^6, 7^ which led to intense scrutiny of the evolution of H9N2 viruses. The increased prevalence of H9N2 viruses in chickens after 2010 enhanced infectivity and antigenic drift.^5^ Furthermore, H9N2 viruses preferentially bind to the human-type sialic acid receptor and spread between ferrets by respiratory droplets.^4^ Between 2013 and 2016, an increasing number of human cases of H9N2 virus infection (*n*=18) were laboratory confirmed in China (http://www.who.int/influenza/human_animal_interface/HAI_Risk_Assessment/en/). From this evidence, it is apparent that H9N2 viruses have undergone considerable changes in their biologic characteristics, but the underlying genetic evolution driving these changes is largely unknown.

In China, H9N2 viruses were initially isolated from domestic ducks during an influenza virus surveillance in Hong Kong from 1976 to 1985.^8^ In the 1980s, the viruses were isolated from quail, which was the first evidence of H9N2 viruses in land-based poultry.^9^ Subsequently, H9N2 viruses spread to chickens in the Guangdong province of Southern China in 1994.^10, 11^ The viruses then adapted to chickens and other land-based birds, such as pheasants, chukars and other minor domestic poultry.^2, 10^ Therefore, quail populations in China most likely contributed to the interspecies transmission of H9N2 viruses from ducks to chickens.^2^ After 1998, the viruses have sporadically crossed to humans, pigs and other mammals in China, causing mild respiratory disease.^10^

Epidemiologic and genetic studies revealed that the hemagglutinin (HA) gene of H9N2 viruses bifurcates into the Eurasian and American lineages.^12^ The Eurasian lineage comprises three distinct sublineages: A/chicken/Beijing/1/94-like (BJ/94-like), A/quail/Hong Kong/G1/97-like (G1-like) and A/duck/Hong Kong/Y439/97 (Y439-like or Korean-like). The BJ/94-like and G1-like viruses mainly circulated in chickens and quails, respectively, in China since the mid-1990s.^10, 12, 13^ However, G1-like viruses also caused outbreaks in chickens in the Middle East and Germany.^2^ Korean-like viruses were first isolated from domestic ducks in Hong Kong in 1997, but similar viruses have been identified in disease outbreaks in chickens in South Korea since 1996.^2^

The internal genes of H9N2 viruses are more diversified than are the surface genes, which is the result of extensive reassortment of H9N2 viruses among chickens, ducks, minor poultry and wild-bird species.^2, 14^ A previous investigation of 330 H9N2 strains isolated from birds, pigs and humans in China from 1994 to 2008 identified 54 genotypes, which were further categorized into five distinct series (that is, the BJ/94, G1, BG, F/98 and Aq series), according to the sources of their genes.^14^ However, the genotype evolution of H9N2 from all hosts since 2009 is not well known.

We previously analyzed the sequence similarities of H9N2 viruses isolated specifically from chickens in China from 1994 to 2013 and found that the genes (especially HA) underwent extensive variation, forming 10 different clades and generating 69 genotypes.^5^ The continuous evolution of H9N2 viral genes resulted in additional phylogenetic groups that exceeded the previously designated clade boundaries in chickens. However, the genetic evolution in other hosts is relatively unknown. Therefore, it is necessary to update the divergent phylogenic clade classification of H9N2 viruses prevailing in China and to perform long-term, systematic genotypic analyses to elucidate the spatiotemporal evolution of H9N2 viruses in various hosts.

In this study, we performed a large-scale genomic sequence analysis of the sequences of 730 H9N2 viruses with all eight gene segments and the sequences of 560 viruses with partial segments. These viruses were isolated in China from 1994 to 2013. We identified 117 genotypes from 730 H9N2 viruses isolated from avian, mammalian and human hosts. In addition, we determined the spatiotemporal changes in genotypic diversity and viral host range.

## Materials and methods

### Sequence collection and alignment

All previously published sequences of H9N2 viruses isolated in China from 1994 to 2013 and related sequences were downloaded from the Influenza Research Database (www.FluDB.org), Global Initiative on Sharing Avian Influenza Data (www.gisaid.org) and the Influenza Virus Resource at the National Center for Biotechnology Information (www.ncbi.nlm.nih.gov/genomes/FLU). The accession numbers of the previously published sequences were summarized in the Supplementary Materials (Supplementary Table S2 and Supplementary Dataset S2). All replicate submissions were removed by identifying sets of isolates with identical sequences for all segments, with each isolate containing one to eight segments. The resulting sequences of each gene segment were aligned with MAFFT v6 software,^15^ manually adjusted to correct frameshift errors, and subsequently translated. Downstream phylogenetic analyses were performed on regions of the alignments containing few gaps across the sequences. These regions included the following intervals: HA g.190_1566, neuraminidase (NA) g.91_1272, nucleoprotein (NP) g.67_1014, nonstructural protein (NS) g.79_807, matrix protein (M) g.88_948, polymerase acidic protein (PA) g.796_2103, polymerase basic protein 1 (PB1) g.61_1437 and polymerase basic protein 2 (PB2) g.1168_2199.

### Phylogenetic analysis and clade classification

The randomized accelerated maximum-likelihood (RAxML7.2.8 Alpha) program was used to construct maximum-likelihood phylogenies for each segment.^16^ The general time-reversible model of nucleotide substitution was assumed, and the rates were assumed to vary according to the discrete gamma distribution with four rate categories. We constructed trees that included all H9N2 strains isolated from all hosts in China and Hong Kong since 1994 (all segments). We used 100-bootstrap maximum-likelihood trees to construct a majority-rule consensus tree from each bootstrap sample with the SumTrees (version 4.0.0) program.^17^ Bayesian phylogenetic trees were constructed for each segment by using the Bayesian evolutionary analysis sampling trees (BEAST version 1.6.1) software.^18^ The model of substitution and rate variation in each tree was duplicated from the corresponding RAxML tree, with the additional assumption of a lognormal distribution molecular clock. The BEAST trees were initiated with the RAxML majority-rule consensus tree to reduce the computation time required for the Markov chain Monte Carlo sampler to reach stationary distribution. Maximum clade credibility trees were constructed by using 2000 equidistant samples from the final 30% of the posterior sample of trees (*n*=25 × 10^6^ samples) with a Python script and tree annotator.^18^ The resulting trees were partitioned into clades with a median branch length distance threshold of 0.20.^19^ Clades with median root-to-tip distances equal to or less than 20% of the median root-to-tip distance of all clades in the tree were selected as clusters. Clades were then manually merged, if necessary, by their branching posterior and reported classification.^2, 14, 20^ Each clade was assigned a unique clade identification number.

### Genotypic analysis

Viral genotypes were analyzed for H9N2 viruses isolated from all hosts in China from 1994 to 2013. Isolates were genotyped if the sequences and clade assignments were available for all eight segments. The genotypes of such isolates (730 isolates with all eight available segments) were determined by the combination of clade assignments of each of the eight segments. Five hundred sixty isolates with partial segments were not included in the genotype analysis. Genotype identification numbers were assigned according to the previous study.^5^ The preceding 69 genotypes (G01–G69) stuck to the already reported genotypes and the additional new genotypes were added subsequently.^5^

### Statistical analysis

Analysis of variance based on general linear model, and three multiple comparison tests (the least-significant difference, Duncan’s multiple-range test and Student–Newman–Keuls test) were performed using SAS (version 9.4). A *P* value <0.05 was considered to indicate statistical significance.

## Results

### Phylogenetic analysis of surface genes

**Figure 1:**
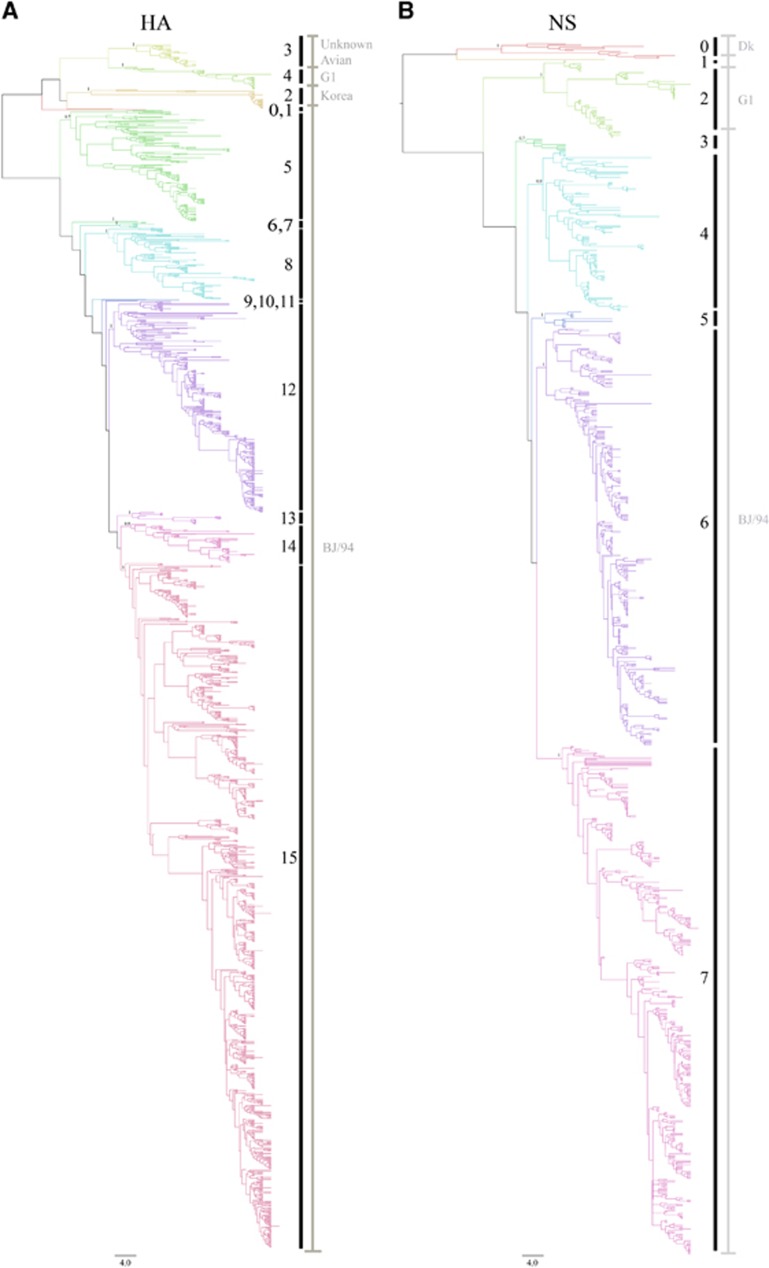
Phylogenetic analysis of H9N2 avian influenza viruses isolated from different hosts in China from 1994 to 2013. (**A**) Phylogenetic tree of the HA gene. (**B**) Phylogenetic tree of the NS gene. Phylogenetic trees of all eight segments with detailed virus information are depicted in Supplementary Figure S1. A summary of information for each clade is listed in Supplementary Table S1. Posterior values are shown for selected clades. Node branch colors indicate specific clades. Vertical black lines with numbers indicate the clade designated in this study, and vertical gray lines with gray words indicate the previous clade classification.

We constructed phylogenetic trees for all eight segments of the H9N2 viruses isolated from various hosts in China from 1994 to 2013. We partitioned the phylogenetic trees into clusters by using a top 20th-percentile cutoff.^19^ We then manually merged the clades, if necessary, by their branching posterior and reported classification.^2, 14, 20^ We found the HA sequences clustered into 16 clades (Figure 1A, Table 1,Supplementary Figure S1, Supplementary Table S1 and Supplementary Dataset S1). According to the nomenclature system for H5N1 influenza viruses defined by the World Health Organization/Organization for Animal Health/Food and Agriculture Organization H5 Evolution Working Group,^21^ the HA clades of H9N2 viruses were rationally named by clade number (0–15).

In our analyses, we observed that 12 independent clades evolved from the ancestral A/chicken/Beijing/1/94 (BJ/94-original) virus, indicative of the extensive variation of the BJ94-like H9 segment in China. Among the BJ/94-original viruses, the majority (1523 of 1607) was distributed in clades 5, 8, 12 and 15; whereas the most recent isolates were consolidated in clade 15. Unlike the BJ/94-original clade, other previously identified clades were relatively stable. We observed that 32 strains with HA genes corresponding with the G1-like clade were clustered in clade 4. Twenty-seven aquatic bird isolates of Korean-like lineage formed the present clade 2. Clade 3, which was the previously identified as an unknown avian clade,^14^ contained 31 strains isolated from quail and other minor avian species from 2000 to 2004. Thus, the *HA* gene of H9N2 viruses has undergone extensive variation, especially from the *HA* gene of the BJ/94-original viruses. Recently, clade 15 has replaced other clades, making it the unique predominant lineage.

According to previous classifications of H9N2 viruses in China, the NA lineages primarily included the BJ/94-like, G1-like and Korean-like lineages.^2, 14^ Here we found that all *NA* genes clustered within four clades (clade 0–3) (Table 1, Supplementary Figure S1, Supplementary Table S1 and Supplementary Dataset S1). Among the 1080 viruses in these clades, 1007 strains constituted clades 1 and 2, which evolved from the BJ/94-like clade. Clade 2 became dominant in China after 2010, and all H9N2 isolates in this clade possessed a three amino-acid deletion in the NA stalk region, which was not present in the isolates in clade 1. Only 27 strains belonged to clade 0, which possessed a Korean-like NA segment. Another small clade (clade 3) that contained G1-like NA strains was primarily detected in quails.

### Phylogenetic analysis of internal genes

The clades of H9N2 internal genes were previously named by host sources (for example, Dk1, Dk2, Dk3, Aq, Swine and unknown avian lineages), representative strains (for example, BJ/94, G1, F/98 and Ty/66 lineages), geographic locations (Korean lineage) or viral subtypes (for example, H5N1-Gx/Gd, H5N1-Z and H5N1-like lineages).^2, 14, 22^ Here we used numbers to name each clade according to the phylogeny of the internal genes, which we also did for the surface genes. All six internal segments were divided into 42 independent clades (Table 1, Supplementary Figure S1, Supplementary Table S1 and Supplementary Dataset S1).

The *PB2* genes of H9N2 viruses were previously classified into seven clades.^14^ However, we found that the *PB2* genes formed nine distinct lineages (clade 0–8) (Table 1 and Supplementary Table S1). Before 2010, clades 0, 6 and 7 were the primary clades with distinct host preferences. Clades 0 and 6 historically consisted of the BJ/94 and F/98 lineages, respectively, which mainly encompassed chicken-origin strains. Clade 7 previously included G1-lineage viruses, which were most frequently isolated from quail and other minor avian species. We found that clade 8 was the most prevalent clade after 2010, with all strains possessing the Dk1-original *PB2* gene. We also observed that clades 1 through 4 split from the root of clade 0. Clade 1 contained two duck strains. Clade 2 constituted a single H9N2 isolate (that is, A/duck/Hong Kong/Y439/1997), which contained the Korean-like *PB2* gene. The strains from clades 3 and 4 possessed H5N1-like *PB2* genes.^2, 14, 22^

In the PB1 phylogenetic tree, H9N2 viruses formed six lineages (clade 0–5) (Table 1 and Supplementary Table S1), unlike the previously identified seven clades.^14^ Clades 0 and 5 were the two largest groups, which were primarily restricted to chicken isolates. Clade 0 was previously characterized by the BJ/94 lineage, and clade 5 evolved from the A/Chicken/Shanghai/F/1998 virus, becoming the most prevalent clade in China after 2010. Clade 1 was also predominant and contained a G1-like PB1 segment, which was mainly isolated from quail and other minor avian species. Unlike most other clades, clade 2 consisted of multiple lineages (Dk1 and Dk2) and was isolated from diverse sources, including chicken, duck, swine and minor avian species. A single isolate of duck origin (A/duck/Shantou/7488/2004) with a Dk3-like PB1 segment constituted clade 3. Seventeen H9N2 strains from different sources, containing H5N1-like *PB1* genes,^2, 14, 22^ formed clade 4.

We observed that eight different lineages of the PA segment, rather than the nine previously described,^14^ formed clades 0 through 7 (Table 1 and Supplementary Table S1). Most of the *PA* genes were limited to clades 6 and 7. Clade 7 included the most recent strains, which contained F/98-like PA segments. However, we could not confirm the source of the *PA* genes in the strains of clade 6, which was previously identified as an unknown avian clade.^2, 14, 22^ Three chicken strains and one duck strain constituted clade 0, and the *PA* genes of these strains were highly related to those of H5N1 viruses.^2, 14, 22^ Both clades 1 and clade 2 contained H9N2 strains of avian and mammalian origins, which possessed BJ/94 and G1-like PA segments, respectively. A single strain of swine origin (A/swine/Guangdong/wxl/2004) and 23 wild waterfowl isolates constituted clade 3. Clade 4, whose strains were mainly isolated between 2000 and 2005 in China, was previously characterized by the Dk1 lineage. Only two strains had Dk2-like PA segments and formed clade 5.

The NP segment segregated into seven lineages, similar to those previously identified,^14^ forming clades 0 through 6. It should be noted that over half of the *NP* genes in the analyzed strains clustered into clade 6, which was previously characterized by the F/98 lineage. The *NP* genes of the clade 5 isolates were apparently derived from the A/goose/Hong Kong/W217/97 (H6N9) strain, which was previously deemed the Aq lineage.^2^ Both clades 0 and 1 were previously characterized by the BJ/94 lineage. Clade 3 consisted of H9N2 isolates from minor poultry and chickens with the G1-like *NP* gene. Only 27 H9N2 strains were of duck and waterfowl origin and contained a Dk-like NP segment and clustered in clade 4.

The *M* gene showed much less diversity than the other segments. Unlike the three previously identified clades,^14^ we categorized the *M* genes into four clades (clades 0–3) (Table 1 and Supplementary Table S1). The M segment of six strains (clade 0) were isolated primarily from chickens, and 27 (clade 1) were derived from viruses of duck origin. All other *M* genes of the H9N2 viruses formed clades 2 and 3, which were previously characterized by the BJ/94 and G1 lineages, respectively. Clade 3 supplanted clade 2 to become the prevalent lineage.

We found considerable diversity in the *NS* genes of H9N2 viruses in China. The number of lineages increased from three, as previously classified,^14^ to eight clades (clades 0–7) (Figure 1B, Table 1 and Supplementary Table S1). Clades 0 and 2 were previously characterized by the Dk and G1 lineages, respectively. The six remaining clades evolved further from the BJ/94 lineage, and clade 7 was predominant in China after 2010.

### Genotyping and chronology

**Figure 2:**
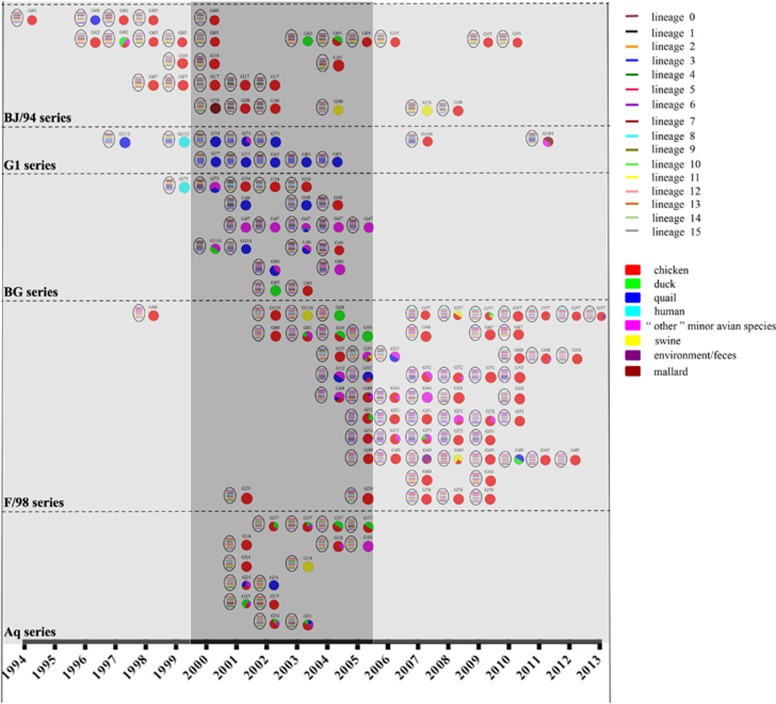
Genotypic prevalence and host distribution of H9N2 avian influenza viruses in China from 1994 to 2013. Virus particles are represented by ovals. The eight gene segments are indicated by horizontal bars within the ovals (from top to bottom: PB2, PB1, PA, HA, NP, NA, M and NS). The color of bars represents a distinct phylogenetic clade. Only the major genotypes that were detected for more than 2 years are represented. All detected genotypes (including major and transient genotypes) are provided in Supplementary Dataset S1. Pie charts indicate the host distribution of major genotypes in corresponding years, and each color represents a host species. Gray-shaded columns represent the three notable periods of H9N2 evolution: 1994 to 1999, 2000 to 2005 and 2006 to 2013.

**Figure 3:**
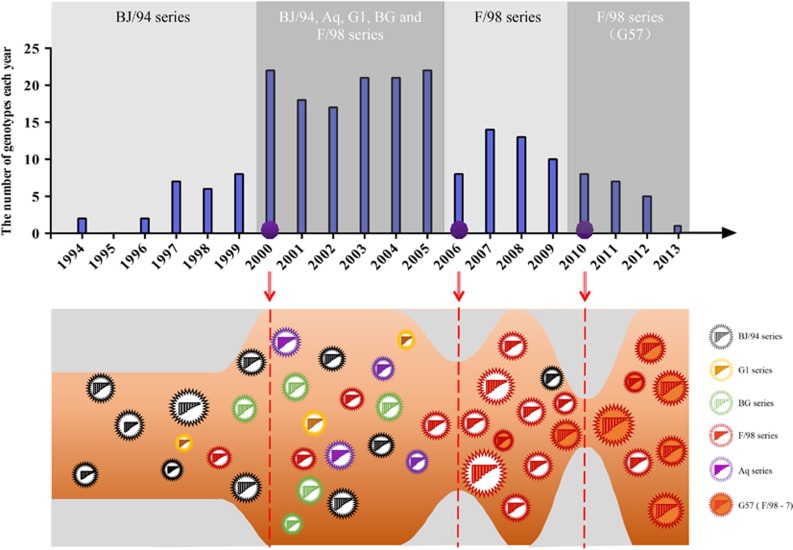
Genotypic diversity of H9N2 avian influenza viruses in China from 1994 to 2013. Top panel: light and dark gray-shaded areas depict distinct periods of H9N2 evolution. The predominant genotypic series of H9N2 viruses are displayed for each corresponding shade. Purple circles indicate three important time points. Bottom panel: the ‘genotype eruption’ in 2000; the ‘sudden decrease’ in 2006; and the ‘selection and subsequent predominance’ of G57 after 2010.

From the newly defined clades, we identified 117 genotypes among all eight segment sequences of 730 H9N2 viruses isolated from 1994 to 2013, including 45 major genotypes and 72 transient genotypes (Figure 2 and Supplementary Dataset S1). During this period, the genotypic diversity underwent two major changes: one genotype eruption in 2000 and two sudden decreases in 2006 and 2010 (Figure 3). Accordingly, we divided the 20-year period into four genotypic evolutionary stages. The difference of the genotypic diversity of H9N2 viruses between any two consecutive time periods was statistically significant (*P*<0.05). In the first stage (1994–1999), fewer genotypes (*n*=19) were prevalent, indicating limited gene reassortment (Figure 3). The predominant genotypes were G01, G02 and G05, which consisted of the BJ/94-original virus (Figure 2). In the second stage (2000–2005), the H9N2 virus experienced a dramatic increase in genetic diversity with 80 prevalent genotypes from cocirculating BJ/94, Aq, G1, BG and F/98 series viruses. The G31 (Aq series) and G47 (BG series) genotypes were the most predominant during this period. In the third stage (2006–2009), the number of genotypes first notably decreased to eight in 2006, and in the following years, the F/98 series outcompeted the remaining series and became predominant (Figure 3). In the last stage (2010–2013), among the various viruses of the F/98 series, the G57 genotype was evolutionarily selected and became the only dominant genotype until the last observed year (that is, 2013) (Figure 3). This is the first observation, to our knowledge, of such a bottleneck in the evolution of H9N2 viruses, despite their prevalence in China for the last 20 years. The G57 genotype generated the novel H7N9 viruses by providing six internal genes from 2011 to 2012.^5^

### Geographic distribution of genotypes

**Figure 4:**
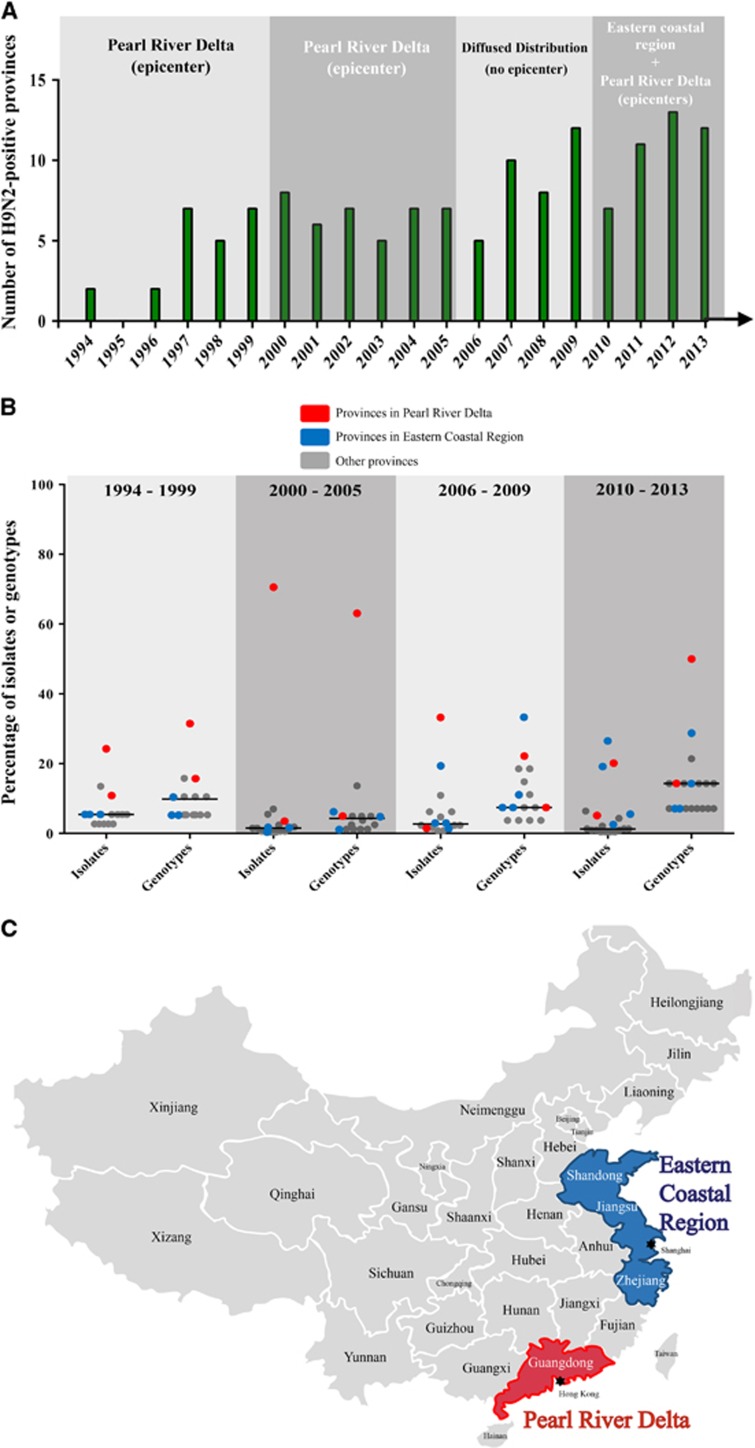
Geographic distribution of H9N2 influenza viruses in China from 1994 to 2013. (**A**) The number of H9N2-positive provinces in China. The primary circulation area in each period is indicated. (**B**) The prevalence of isolates and genotypes in their corresponding provinces for each period. The proportion of the isolates (or genotypes) from each province relative to the total number of isolates in all H9N2-positive provinces was calculated. Red dots represent the proportion of provinces in the Pearl River Delta region; blue dots represent the proportion of provinces in the eastern coastal region, and gray dots represent the proportion of all other provinces. (**C**) Map of the Pearl River Delta and eastern coastal regions in China.

The three critical time points of H9N2 viral evolution (2000, 2006 and 2010) were notably associated with the geographic distribution of the viruses (Figure 4 and Supplementary Dataset S1). We calculated the proportion of isolates (or genotypes) from each province relative to those from all H9N2-positive provinces to determine the epicenter of H9N2 viral outbreaks in China (Figures 4A and 4B). Before 2000, the total number of H9N2 isolates was relatively small, but the viruses were present in 15 provinces. Most of the strains were isolated in the Pearl River Delta region in southern China (including Guangdong province and Hong Kong). Between 2000 and 2005, H9N2 viruses were present in 16 provinces but were generally centralized in the Pearl River Delta region (Figures 4B and 4C). Specifically, in this region, the proportion of isolates in Guangdong province was 70.6%, and the proportion in Hong Kong was 3.6%. The proportion of H9N2 isolates in both areas was close to or higher than the median level of 1.5% in the total H9N2-positive provinces. Similarly, the proportion of genotypes identified in the Guangdong province and Hong Kong was 63.0% and 4.9%, respectively, which also accounted for the greatest frequency of H9N2 genotypes in China.

From 2006 to 2009, H9N2 viral genetic diversity sharply decreased, and the prevalence of isolates was diffusely distributed among the 16 provinces. However, the proportions of isolates and genotypes in Shandong province and Hong Kong were relatively higher. After 2010, the Pearl River Delta and eastern coastal regions became the concomitant epicenters of H9N2 viral outbreaks (Figures 4B and 4C). We defined the eastern coastal region of China as the Shandong, Jiangsu and Zhejiang provinces, including the municipality of Shanghai (Figure 4C).

In 2010, the number of H9N2 genotypes decreased and the virus was only isolated in seven provinces. After 2010, however, the number of affected provinces sharply rose to 19, and the eastern coastal region was the site of more frequent viral circulation (Figure 4B). This phenomenon illustrated that the increased prevalence of H9N2 viruses played a central role in generating novel H7N9 genotypes in East China in 2013.^5^ Together, our findings demonstrate that H9N2 viruses originating from South China spread throughout the country over 20 years, and the Pearl River Delta and the newly identified Eastern Coastal Regions are the two epidemic centers. Additional surveillance is necessary to fully appreciate the geographic distribution of H9N2 viruses in China.

### Host range of genotypes

We detected H9N2 viruses isolated from 1994 to 2013 in 22 host species of birds, mammals and humans (Supplementary Dataset S1). Based on our data, chickens and the minor avian species (include the quail and ‘other’ minor avian species) were the most prevalent hosts. We found that 60.1% of the isolates and 66.7% of the genotypes were isolated from chickens, and 17.1% of the isolates and 43.6% of the genotypes were isolated from the minor avian species. The G1 series was primarily restricted to quail and the ‘other’ minor avian species, similar to that of previous findings.^2, 22^ In contrast, the remaining series were distributed in multiple hosts (Figure 2).

**Figure 5:**
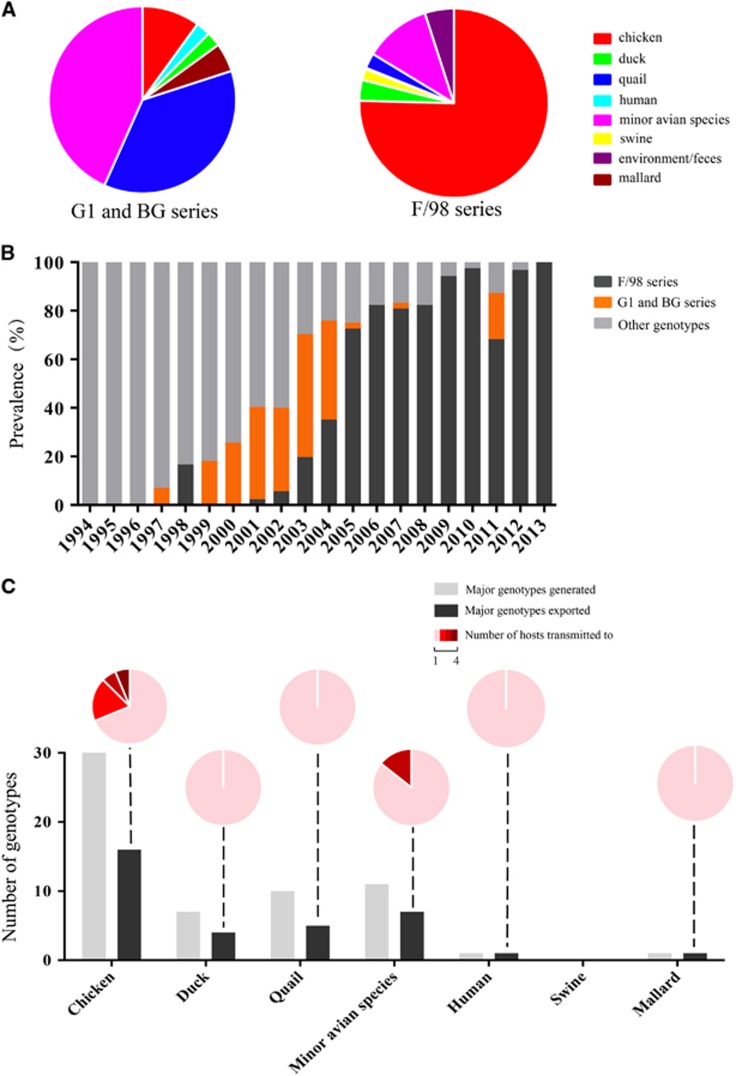
Prevalence in hosts and interspecies transmission of the major genotypes of H9N2 avian influenza viruses. (**A**) The proportion of host distribution of the G1 and BG or F/98 series of H9N2 viruses. (**B**) The prevalence of the G1 and BG (orange bars), F/98 (black bars) or other (gray bars) series of H9N2 viruses in China from 1994 to 2013. The G1 and BG series primarily circulated in quail and other minor poultry. The F/98 series primarily circulated in chickens. (**C**) Interspecies transmission of the major genotypes of H9N2 viruses. The histogram depicts the total number of major genotypes generated (gray bars) and the major genotypes exported to different hosts (black bars). The interspecies transmission ability of the major genotypes from each host is illustrated in pie charts. For example, in chickens, 16 genotypes were exported to other hosts, and 11 of these transmitted to only one different host (pink), three transmitted to two different hosts (red), one transmitted to three different hosts (dark red) and one transmitted to four different hosts (maroon).

**Figure 6:**
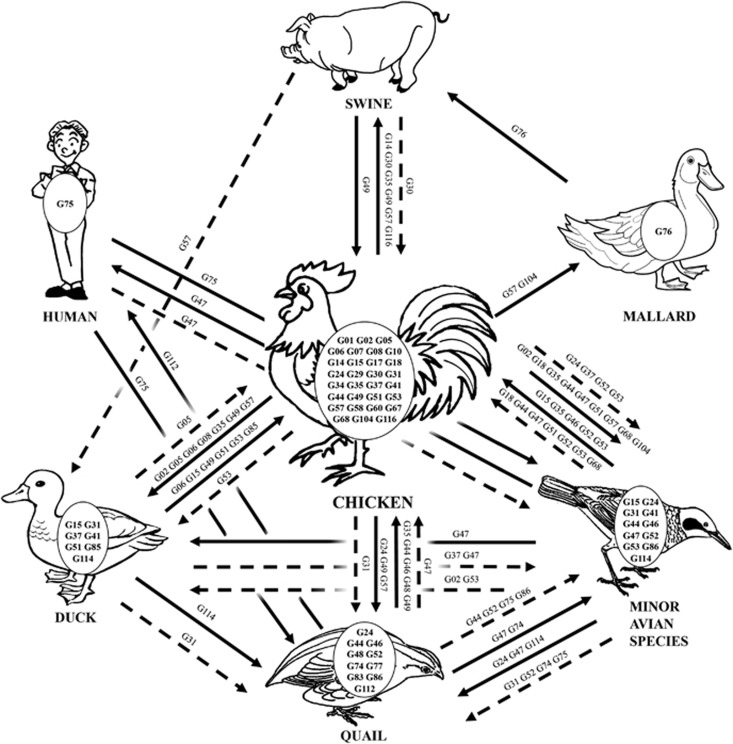
Ecology of H9N2 avian influenza viruses in China from 1994 to 2013. A representative diagram illustrates the interspecies transmission events of the major genotypes of H9N2 viruses. The major genotypes generated in each host are displayed in ovals within each host pictures. Solid lines represent confirmed gene flow directions. Dashed lines indicate indirect evidence of gene flow.

Interspecies transmission of H9N2 viruses among multiple hosts affected the viral ecologic system. Previous studies of epidemiologic data collected before 2005 revealed that quail contributed to the interspecies transmission of H9N2 viruses from ducks to chickens and played an important role in generating new genotypes.^22^ Our data confirm this finding. We found that quail conferred the generation of the BG series, which were reassortants between the G1 and BJ/94 series.^14, 22^ In addition, quail transmitted the G1 and BG series (or their viral genes) to other hosts (Figures 5A and 5B), including humans in 1999 (Figure 2 and Supplementary Dataset S1). However, after 2005, the G1 and BG series gradually decreased to the low level and the F/98 series became dominant. The F/98 series was initially isolated in chickens, increasingly prevalent in this host (Figures 5A and 5B) and transmitted to other animals (Figure 2), revealing an important role of chickens in the viral ecologic system. Of the 45 major genotypes isolated, 30 were generated in chickens, and 16 out of 30 were transmitted to other hosts (Figure 5C). The genotypes exported from chickens demonstrated the greatest ability to transmit to multiple hosts (*n*≥2), indicative of frequent viral transmission or wider viral host tropism of these genotypes (Figure 5C). Consequently, chickens, which account for the biggest poultry population in China, rather than quails, became a critical host for generating and transmitting these genotypes (Figure 6).

We observed that reassortment events within specific segments changed the host range of H9N2 viruses (Table 2). For example, after acquiring the PB1 segment of clade 4, the host range of the G116 genotype in chickens and swine expanded to the G35 genotype in chickens, swine, ducks, quails and the ‘other’ minor avian species. The newly identified clade 15 HA segment also exerted a similar effect. The G49 and G57 genotypes, which contain the clade 15 HA, were transmitted to three additional hosts than were the G58 and G68 genotypes, which had same segment constellation, except for the *HA* gene. In addition, the *PB2* gene of clade 8 and the *PA* gene of clade 6 bestowed similar enhanced transmission capability.

## Discussion

In this study, we analyzed the phylogenetic and genotypic evolution of H9N2 viruses isolated from various hosts from 1994 to 2013 in China. We updated the clade classification of H9N2 viruses and identified 117 genotypes, including 45 major and 72 transient genotypes. We elucidated three important time points (2000, 2006 and 2010) in the evolution of these viruses in China. In the periods divided by these time points, the genotypic diversity, geographic distribution and host range of H9N2 viruses underwent considerable changes.

We previously performed a phylogenetic study of H9N2 viruses isolated specifically from chickens in China from 1994 to 2013.^5^ To understand the ecology of H9N2 generation and transmission, we employed the same strategy to analyze the genotypic evolution of the H9N2 viruses from all hosts in China from 1994 to 2013. Compared with the traditionally classified clades, the clade classification advanced here revealed great changes in the *HA* and *NS* genes of these viruses. The BJ/94-original *HA* and *NS* genes further evolved into 13 and 5 clades, respectively. Among these newly identified clades, clade 15 HA and clade 7 NS were components of the predominant G57 genotype, which contributed six internal genes to the novel H7N9 viruses.^5^ Moreover, the reassortment of the clade 15 *HA* gene seems to increase the viral host range. Therefore, it is necessary to continually update H9N2 clade classification to follow their genetic evolution. Such continual updating of clade classification is well established in the surveillance of H5 viruses.^21, 23^

Based on the clade classification, we analyzed the genotypic evolution of H9N2 viruses in China during 1994–2013. The period from 1994 to 1999 represents an initial era of adaptation and transmission of H9N2 viruses from aquatic birds to terrestrial poultry in China. H9N2 viruses originally circulated in the Pearl River Delta region in southern China, and gradually spread to 15 provinces. At this time, the G1, BJ/94, BG and F/98 genotypic series were restricted to quail, chickens and other minor avian species, with few gene exchanges among these species.

In the following period between 2000 and 2005, the genotypic diversity of H9N2 viruses greatly increased, which resulted from frequent gene exchanges within different hosts. This was particularly evident in the G1-like genes and the Korean-like genes in quail and other minor avian species, which reassorted with the BJ/94-like and F/98-like series to enhance the diversity of their genotypes. This phenomenon was also observed in other studies.^2, 14^ The increase in genotypic diversity of H9N2 viruses in China may be explained by two hypotheses. The first hypothesis suggests that chickens, which are the largest avian population, increased the concentration of H9N2 viruses in one region, which greatly contributed to viral transmission between chickens and other hosts. The second hypothesis suggests that immune pressure within the chicken population contributed to the diversification of H9N2 genotypes. Since the late 1990s, chicken farmers in China have administered commercially inactivated vaccines raised against the BJ/94 and F/98 series to control H9N2 viral infections. This immune pressure may drive H9N2 viruses to become more adaptive through reassortment with G1-like and the Korean-like genes from quail and wild birds, respectively. During the second period of our analysis, the extent of the geographic distribution of H9N2 viruses was not considerably changed, and the Pearl River Delta region remained the epidemic center.

After 2006, the diversified genotypes of the F/98 series generated in chickens were evolutionarily selected and become predominant, while the genotypes of the other series gradually decreased. This suggests that H9N2 viruses adapted by selecting the most advanced genotype. Subsequently, in 2010, the G57 genotype was further selected from the F/98 series and became the only predominant genotype. This is the first such event to occur during the 20-year prevalence of H9N2 viruses in China. We previously revealed that this G57 genotype contributed to the country-wide outbreak of H9N2 viruses in chickens and the subsequent emergence of the novel H7N9 reassortant with six H9N2 internal genes.^5^ The G57 genotype was found in 2007 and initially prevalent in the eastern coastal region. In the 2010–2013 period, the eastern coastal region became the more important epicenter of H9N2 infections. Like Pearl River Delta, the environmental factors, such as warm climate, bird migration, the developed poultry industries and trade, may facilitate the distribution of H9N2 viruses in the eastern coastal region. In the third period between 2006 and 2013, H9N2 evolution has been largely characterized by G57 genotypic selection and expansion.

Since H9N2 viruses were isolated in farmed chickens and became endemic, this host has played an increasingly important role in the evolution of *H9N2* genes by generating and transmitting novel genotypes. Since 2010, the chicken-origin H9N2 viruses account for the majority H9N2 strains. Besides, the genotypes generated in chickens have shown changed biologic properties. Particularly, the recent predominant G57 genotype was generated in chickens and transmitted to other birds and mammals. Despite no human-origin G57 H9N2 strains were found in our data, it was reported that this genotype was isolated from a human patient in Hong Kong at the year of 2014.^5^ These recent H9N2 strains exhibited increased infectivity and transmission in chickens and ferrets,^4, 5^ which creates serious concerns for public health. In addition, the G57 H9N2 viruses have provided genes to generate 11 novel reassortants of subtypes other than H7N9 (for example, H5N2, H7N7 and H10N8 viruses). These novel reassortants were isolated in birds, swine and humans primarily after 2010.^5^ From 2013 to 2016, an increasing number of human H9N2 cases (*n*=18) were confirmed in China (http://www.who.int/influenza/human_animal_interface/HAI_Risk_Assessment/en/). Therefore, chickens have profoundly affected the evolution of H9N2 viruses.

In this analysis, we included all the available 8162 sequences of H9N2 influenza viruses isolated in China from 1994 to 2013 uploaded in the NCBI, FluDB and GISAID, however the sample bias or the limitation of sequences on hosts and geographical coverage cannot be avoided, which may not be possible to adequately profile the emergence and evolution of H9N2 viruses. But, our findings of the H9N2 genetic evolution had the match with those of previous studies on understanding the viral behaviors. Statistical analysis showed that the genotypic diversity of H9N2 viruses between any two consecutive time periods was changed significantly (*P*<0.05). The important time points of 2000,^2, 14^ 2006^14^ and 2010^5^ were also, respectively, indicated in different studies. The changes of genotypes in poultry species that we found have good correspondences with the isolation rate (or prevalence) in these hosts demonstrated by previous epidemiological surveys.^2, 5, 24, 25, 26^ Recent phylogeography study also confirmed our results about the geographic distribution of H9N2 viruses in China.^27^ Our study presents a comprehensive summary of H9N2 evolution in the recent decades. However, more systematic epidemiological surveillance and analysis are urgently needed to better understand the circulation of H9N2 influenza virus in China.

In summary, our findings indicate that throughout the 20-year prevalence of H9N2 viruses in China, minor avian species and wild birds exacerbate the genotypes by providing diversified genes, and chickens act as a vector, permitting genetic selection of potentially virulent genotypes and expanding prevalence. In this ecologic environment, H9N2 viruses appear to have evolved into an adaptive pathogen with a wide host range. Not surprisingly, the infectivity of H9N2 viruses has increased rapidly, especially in mammals. H9N2 viruses are predicted to enter into another evolutionary phase with increased genotypic diversity and accelerated changes in biologic characteristics. In addition to live-bird markets, poultry farms and wild-bird habitats are important sites for surveillance and disease control.

## Figures and Tables

**Table 1:** Comparison of clade classifications in the current and previous studies^a^

**Segment**	**Previous clade**	**Current clade**
HA	*n*=4 (BJ/94, G1, Unknown avian, Korea)	*n*=16 (clade 0–clade 15)
NA	*n*=3 (BJ/94, G1, Korea)	*n*=4 (clade 0–clade 3)
PB2	*n*=9 (BJ/94, Korea, Dk1, Dk2, GX, F/98, H5N1-GS/GD, H5N1-Z, G1)	*n*=9 (clade 0–clade 8)
PB1	*n*=7 (BJ/94, G1, Dk1, Dk2, Dk3, H5N1-like, F/98)	*n*=6 (clade 0–clade 5)
PA	*n*=8 (H5N1-like, BJ/94, G1, Swine, Dk1, Dk2, Unknown avian, F/98)	*n*=8 (clade 0–clade 7)
NP	*n*=6 (BJ/94, G1, Dk, Aq, Ty/66, F/98)	*n*=7 (clade 0–clade 6)
M	*n*=3 (BJ/94, G1, Dk)	*n*=4 (clade 0–clade 3)
NS	*n*=3 (BJ/94, G1, Dk)	*n*=8 (clade 0–clade 7)

Abbreviations: Beijing, BJ; duck, Dk; Guangdong, GD; goose, Gs; Hong Kong, HK; turkey, Ty.

a The previous clade classification was referred to ref.^14^

**Table 2:** Reassortment with specific segments change the host range of H9N2 influenza virus

**Pair^a^**	**Genotype**	**Variable clade/segment**	**Host range**
1	G49	6/PB2	Chicken, duck, quail, swine
	G57	8/PB2	Chicken, duck, quail, mallard, swine, minor avian species
2	G116	5/PB1	Chicken, swine
	G35	4/PB1	Chicken, duck, quail, swine, minor avian species
3	G75	2/PA	Human, quail, minor avian species
	G47	6/PA	Chicken, duck, human, quail, minor avian species
4	G58	14/HA	Chicken
	G49	15/HA	Chicken, duck, quail, swine
5	G68	12/HA	Chicken, minor avian species
	G57	15/HA	Chicken, duck, quail, mallard, swine, minor avian species

Abbreviations: hemagglutinin, HA; polymerase acidic protein, PA; polymerase basic protein 1, PB1; polymerase basic protein 2, PB2.

a In each pair, the two genotypes of viruses have the same genetic constellation except the specific exchangeable clades of corresponding segment.
