# Supplementary material for: Genetic evolution of influenza H9N2 viruses isolated from various hosts in China from 1994 to 2013
Source: Emerg Microbes Infect. 2017 Nov 29;6(11):e106–. doi: 10.1038/emi.2017.94 (PMC5717095; doi:10.1038/emi.2017.94)
Supplement: Supplementary Table S1 [file emi201794x1.docx]

**Supplementary Table S1** Information of clades for H9N2 influenza viruses isolated in China from 1994 to 2013.

| **Segment** | **Clade** | **Year** | **No. isolates** | | **Isolation source** | **Representative strain** |  |
| --- | --- | --- | --- | --- | --- | --- | --- |
| HA | 0 | 1998 | 1 | Ck | | Ck/Shaoguan/13/98 |  |
|  | 1 | 1994 | 1 | Ck | | Ck/HK/739/94 |  |
|  | 2 | 1997 - 2012 | 27 | Dk, MAS | | Dk/HK/Y439/97 |  |
|  | 3 | 2000 - 2004 | 31 | Qa, MAS | | Qa/ST/4700/2002 |  |
|  | 4 | 1997 - 2013 | 32 | Ck, Qa, Hu, Ma | | Qa/HK/G1/97 |  |
|  | 5 | 1994 - 2008 | 157 | Ck, Dk, Hu, Qa, MAS, Sw | | Ck/BJ/1/94 |  |
|  | 6 | 1997 - 1998 | 3 | Dk, Sw | | Dk/HK/w213/97 |  |
|  | 7 | 1997 - 1999 | 6 | Ck, MAS | | Ck/HK/G9/97 |  |
|  | 8 | 1997 - 2011 | 101 | Ck, Dk, Qa, Sw, MAS | | Ck/SC/5/97 |  |
|  | 9 | 1998 | 1 | Hu | | A/Shantou/239/98 |  |
|  | 10 | 2002 | 1 | Ck | | Ck/GD/126/02 |  |
|  | 11 | 1997 | 1 | Dk | | Dk/HK/Y280/97 |  |
|  | 12 | 1996 - 2012 | 298 | Ck, Dk, Qa, Sw, MAS | | Qa/SH/8/96 |  |
|  | 13 | 1998 - 2007 | 17 | Ck, Hu, Dk, Sw, MAS | | Ck/BJ/9/98 |  |
|  | 14 | 1998 - 2011 | 55 | Ck, Dk, Hu, Sw | | A/Shaoguan/408/98 |  |
|  | 15 | 1999 - 2013 | 967 | Ck, Dk, Qa, MAS, Hu, Sw, Eq, En, Fe, Ca, Ma | | Ck/SD/JN/99 |  |
| NA | 0 | 1997 - 2012 | 27 | Dk, MAS | | Dk/HK/Y439/97 |  |
|  | 1 | 1997 - 2012 | 368 | Ck, Dk, Eq, Hu, Qa, Sw, Ca, Ma, MAS | | Ck/HK/G9/97 |  |
|  | 2 | 1994 - 2013 | 639 | Ck, Dk, Qa, Sw, En, Fe, Hu, Ma, MAS | | Ck/BJ/1/94 |  |
|  | 3 | 1997 - 2005 | 46 | Ck, Qa, Hu, MAS | | Qa/HK/G1/97 |  |
| PB2 | 0 | 1994 - 2010 | 74 | Ck, Dk, Qa, Sw, MAS | | Ck/BJ/1/94 |  |
|  | 1 | 2008 | 2 | Dk | | Dk/JS/1/08 |  |
|  | 2 | 1997 | 1 | Dk | | Dk/HK/Y439/97 |  |
|  | 3 | 2005 | 2 | Ck | | Ck/GX/521/05 |  |
|  | 4 | 2000 - 2012 | 29 | Ck, Qa, Sw, MAS | | Ck/China/Guangxi1/00 |  |
|  | 5 | 2000 - 2008 | 75 | Ck, Dk, Qa, Sw, MAS | | Ck/GX/6/00 |  |
|  | 6 | 1998 - 2012 | 271 | Ck, Dk, Sw, Qa, En, Fe, MAS | | Ck/SH/F/98 |  |
|  | 7 | 1997 - 2011 | 172 | Ck, Dk, Hu, Ma, Qa, MAS | | Qa/HK/G1/97 |  |
|  | 8 | 2004 - 2013 | 258 | Ck, Dk, En, Eq, Hu, Ma, Qa, Sw, MAS | | Dk/ST/7488/04 |  |
| PB1 | 0 | 1994 - 2010 | 227 | Ck, Dk, Qa, Sw, MAS | | Ck/BJ/1/94 |  |
|  | 1 | 1997 - 2011 | 168 | Ck, Dk, Hu, Ma, Qa, Sw, MAS | | Qa/HK/G1/97 |  |
|  | 2 | 1997 - 2012 | 91 | Ck, Dk, Qa, Sw, MAS | | Dk/HK/Y439/97 |  |
|  | 3 | 2004 | 1 | Dk | | Dk/ST/7488/04 |  |
|  | 4 | 2004 - 2006 | 17 | Ck, Dk, Qa, Sw, MAS | | Ck/GX/521/05 |  |
|  | 5 | 1998 - 2013 | 346 | Ck, Dk, En, Eq, Fe, Eq, Hu, Qa, Sw, Ca, MAS | | Ck/SH/F/98 |  |
| PA | 0 | 2000 - 2007 | 4 | Ck, Dk | | CK/China/Guangxi1/00 |  |
|  | 1 | 1994 - 2010 | 75 | Ck, Dk, Qa, Sw, MAS | | Ck/BJ/1/94 |  |
|  | 2 | 1997 - 2002 | 33 | Ck, Qa, Hu, MAS | | Qa/HK/G1/97 |  |
|  | 3 | 2004 - 2012 | 24 | Sw, MAS | | Sw/GD/wxl/04 |  |
|  | 4 | 1999 - 2008 | 78 | Ck, Dk, Qa, Sw, MAS | | Dk/ST/2143/00 |  |
|  | 5 | 2004 - 2011 | 2 | Dk, MAS | | Dk/ST/163/04 |  |
|  | 6 | 1997 - 2011 | 264 | Ck, Dk, Ma, Hu, Qa, MAS | | Ck/HK/NT142/03 |  |
|  | 7 | 1998 - 2013 | 369 | Ck, Dk, Eq, En, Fe, Hu, Ma, Qa, Sw, Ca, MAS | | Ck/SH/F/98 |  |
| NP | 0 | 2002 | 1 | Dk | | Dk/JS/nf/02 |  |
|  | 1 | 1994 - 2010 | 166 | Ck, Dk, Hu, Qa, Sw, MAS | | Ck/BJ/1/94 |  |
|  | 2 | 1998 - 2000 | 2 | Ck | | Ck/HLJ/u/98 |  |
|  | 3 | 1997 - 2011 | 79 | Ck, Hu, Ma, Qa, MAS | | Qa/HK/G1/97 |  |
|  | 4 | 1997 - 2011 | 27 | Dk, MAS | | Dk/HK/Y439/97 |  |
|  | 5 | 2000 - 2008 | 74 | Ck, Dk, Qa, Sw, MAS | | GF/ST/1677/00 |  |
|  | 6 | 1998 - 2013 | 588 | Ck, Dk, En, Eq, Fe, Qa, Sw, Hu, Ma, Ca MAS | | Ck/SH/F/98 |  |
| M | | 0 | 1998 - 2013 | 6 | Ck, Sw | | Ck/HN/nd/98 |
|  |  | 1 | 1997 - 2012 | 29 | Dk, Sw, MAS | | Dk/HK/Y439/97 |
|  |  | 2 | 1994 - 2010 | 362 | Ck, Dk, Qa, Sw, MAS | | Ck/BJ/1/94 |
|  |  | 3 | 1997 - 2013 | 540 | Ck, Dk, En, Eq, Fe, Qa, Sw, Hu, Ma, Ca MAS | | Qa/HK/G1/97 |
| NS | 0 | 1997 - 2011 | 12 | Ck, Dk, MAS | | Dk/HK/Y439/97 |  |
|  | 1 | 2002 | 1 | Ck | | Ck/BJ/nl/02 |  |
|  | 2 | 1997 - 2011 | 60 | Ck, Hu, Ma, Qa, MAS | | Qa/HK/G1/97 |  |
|  | 3 | 1994 - 2002 | 9 | Ck | | Ck/BJ/1/94 |  |
|  | 4 | 1997 - 2010 | 123 | Ck, Dk, Qa, Sw, MAS | | Ck/HK/G9/97 |  |
|  | 5 | 1995 - 2007 | 13 | Ck, Dk, MAS | | Ck/BJ/1/95 |  |
|  | 6 | 1996 - 2011 | 319 | Ck, Dk, Eq, Hu, Qa, Sw, MAS | | Qa/SH/8/96 |  |
|  | 7 | 1998 - 2013 | 389 | Ck, Dk, En, Fe, Hu, Ma, Qa, Sw, MAS | | Ck/SH/F/98 |  |

Abbreviations: Beijing, BJ; canine, Ca; chicken, Ck; duck, Dk; environment, En; equine, Eq; feces, Fe; Guangdong, GD; Guangxi, GX; Heilongjiang, HLJ; Hong Kong, HK; human, Hu; Jiangsu, JS; minor avian species, MAS; number of, No.; quail, Qa; Sichuan, SC; Shandong, SD; Shanghai, SH; Shantou, ST; swine, Sw.
