# Supplementary material for: Genetic evolution of influenza H9N2 viruses isolated from various hosts in China from 1994 to 2013
Source: Emerg Microbes Infect. 2017 Nov 29;6(11):e106–. doi: 10.1038/emi.2017.94 (PMC5717095; doi:10.1038/emi.2017.94)
Supplement: Supplementary Table S2 [file emi201794x2.docx]

**Supplementary Table S2** Accession numbers of the previously published sequences included in the phylogenetic analysis.

| PB2 | |  | PB1 | |  | PA | |  | HA | |
| --- | --- | --- | --- | --- | --- | --- | --- | --- | --- | --- |
| DQ064566 | NCBI |  | CY023742 | NCBI |  | DQ485215 | NCBI |  | KF971981 | NCBI |
| DQ981535 | NCBI |  | KF260620 | fludb |  | DQ485223 | NCBI |  | AF156377 | NCBI |
| AF508661 | NCBI |  | CY023606 | NCBI |  | DQ485207 | NCBI |  | KF972061 | NCBI |
| DQ064568 | NCBI |  | EF155407 | NCBI |  | HQ425333 | NCBI |  | KF972109 | NCBI |
| EU914201 | NCBI |  | JF916719 | NCBI |  | AF156452 | NCBI |  | AY043014 | NCBI |
| AF508655 | NCBI |  | DQ064541 | NCBI |  | EU532057 | NCBI |  | CY024016 | NCBI |
| AF508653 | NCBI |  | JQ904460 | NCBI |  | CY075053 | NCBI |  | KF971997 | NCBI |
| AF222629 | NCBI |  | KF260567 | NCBI |  | DQ064508 | NCBI |  | KF971965 | NCBI |
| CY075027 | NCBI |  | KF260587 | NCBI |  | CY075037 | NCBI |  | KF972021 | NCBI |
| EU914197 | NCBI |  | CY023366 | NCBI |  | DQ064514 | NCBI |  | KF972069 | NCBI |
| JQ904459 | NCBI |  | CY023790 | NCBI |  | AF156445 | NCBI |  | KF972037 | NCBI |
| JF916710 | NCBI |  | KF746865 | NCBI |  | AF508674 | NCBI |  | KF972013 | NCBI |
| JQ639775 | NCBI |  | KF260597 | NCBI |  | DQ064510 | NCBI |  | KF972053 | NCBI |
| CY024693 | NCBI |  | KF260591 | NCBI |  | JF795135 | NCBI |  | KC162234 | NCBI |
| CY075035 | NCBI |  | DQ226169 | NCBI |  | CY075045 | NCBI |  | KF972085 | NCBI |
| EU914194 | NCBI |  | CY023782 | NCBI |  | EU532061 | NCBI |  | JX437685 | NCBI |
| AF222630 | NCBI |  | DQ064542 | NCBI |  | EU532058 | NCBI |  | AF156379 | NCBI |
| CY077082 | NCBI |  | KF746754 | NCBI |  | KF746755 | NCBI |  | KF972101 | NCBI |
| DQ981567 | NCBI |  | AF508629 | NCBI |  | DQ064512 | NCBI |  | KF972029 | NCBI |
| AF222631 | NCBI |  | CY005511 | NCBI |  | KF746809 | NCBI |  | CY024032 | NCBI |
| AF508651 | NCBI |  | CY023526 | NCBI |  | AF156450 | NCBI |  | KF971957 | NCBI |
| DQ064543 | NCBI |  | KF260573 | NCBI |  | KF746834 | NCBI |  | KF972077 | NCBI |
| AF508658 | NCBI |  | KF260603 | NCBI |  | AF508677 | NCBI |  | KF972045 | NCBI |
| EU502898 | NCBI |  | CY055154 | NCBI |  | AF508680 | NCBI |  | KF971973 | NCBI |
| DQ465397 | NCBI |  | KF746874 | NCBI |  | DQ064503 | NCBI |  | KF972117 | NCBI |
| AF156433 | NCBI |  | CY023510 | NCBI |  | CY075029 | NCBI |  | KF971949 | NCBI |
| CY024629 | NCBI |  | CY024694 | NCBI |  | DQ064511 | NCBI |  | KF972093 | NCBI |
| CY075043 | NCBI |  | CY023758 | NCBI |  | CY005516 | NCBI |  | KF971989 | NCBI |
| KF746824 | NCBI |  | FJ013035 | NCBI |  | JF916712 | NCBI |  | KF972005 | NCBI |
| DQ064545 | NCBI |  | AF156423 | NCBI |  | DQ064495 | NCBI |  | EF154913 | NCBI |
| DQ064544 | NCBI |  | CY023398 | fludb |  | CY005510 | NCBI |  | EF154914 | NCBI |
| EU914198 | NCBI |  | DQ226170 | NCBI |  | KF746761 | NCBI |  | CY024560 | NCBI |
| AF222627 | NCBI |  | KF260613 | NCBI |  | DQ064497 | NCBI |  | EF154939 | NCBI |
| DQ981559 | NCBI |  | CY023478 | NCBI |  | AF508675 | NCBI |  | EF154948 | NCBI |
| AF508652 | NCBI |  | EF155403 | NCBI |  | DQ064490 | NCBI |  | EF154952 | NCBI |
| AF222816 | NCBI |  | KF746833 | NCBI |  | KF746875 | NCBI |  | EF154909 | NCBI |
| AF222628 | NCBI |  | CY023518 | NCBI |  | AF156444 | NCBI |  | EF154960 | NCBI |
| KF746759 | NCBI |  | CY023590 | NCBI |  | JF916720 | NCBI |  | EF154912 | NCBI |
| CY005518 | NCBI |  | CY023598 | NCBI |  | AF508673 | NCBI |  | EF154918 | NCBI |
| AF156436 | NCBI |  | KF260608 | NCBI |  | DQ064489 | NCBI |  | EF154922 | NCBI |
| EU502906 | NCBI |  | CY077081 | NCBI |  | DQ064509 | NCBI |  | EF154937 | NCBI |
| CY075051 | NCBI |  | CY024630 | NCBI |  | EU532060 | NCBI |  | EF154956 | NCBI |
| DQ064564 | NCBI |  | KF260570 | NCBI |  | AF156447 | NCBI |  | EF154941 | fludb |
| DQ064565 | NCBI |  | KF260592 | NCBI |  | DQ064515 | NCBI |  | EF154962 | NCBI |
| DQ064563 | fludb |  | DQ226166 | NCBI |  | AF156446 | NCBI |  | EF154908 | NCBI |
| AF156438 | NCBI |  | KF260576 | NCBI |  | DQ064500 | NCBI |  | EF154938 | NCBI |
| EU516317 | NCBI |  | DQ064522 | NCBI |  | DQ064505 | NCBI |  | EF154923 | NCBI |
| KF746753 | NCBI |  | KF260614 | NCBI |  | CY023863 | fludb |  | EF154949 | NCBI |
| DQ064562 | NCBI |  | KF260595 | NCBI |  | CY024631 | NCBI |  | EF154961 | NCBI |
| DQ064558 | NCBI |  | JF916711 | NCBI |  | JQ639785 | NCBI |  | EF154942 | NCBI |
| DQ064560 | NCBI |  | CY024654 | NCBI |  | JQ639777 | NCBI |  | EF154926 | NCBI |
| JF795133 | NCBI |  | KF260563 | NCBI |  | DQ064506 | NCBI |  | EF154936 | NCBI |
| DQ064561 | NCBI |  | CY075036 | NCBI |  | DQ064504 | NCBI |  | EF154955 | NCBI |
| HQ425331 | NCBI |  | JF795122 | NCBI |  | CY023895 | NCBI |  | CY023096 | NCBI |
| DQ064554 | NCBI |  | GQ373050 | NCBI |  | CY024639 | NCBI |  | EF154959 | NCBI |
| DQ064547 | NCBI |  | CY024438 | NCBI |  | DQ465399 | NCBI |  | EF154917 | NCBI |
| DQ064546 | NCBI |  | CY023390 | NCBI |  | EU086295 | NCBI |  | EF154921 | NCBI |
| DQ226176 | NCBI |  | EU882861 | NCBI |  | EU502900 | NCBI |  | EF154930 | NCBI |
| KF746873 | NCBI |  | KF260569 | NCBI |  | DQ226154 | NCBI |  | EF154958 | NCBI |
| AF222817 | NCBI |  | KF260594 | NCBI |  | EF070738 | NCBI |  | EF154924 | NCBI |
| DQ981591 | NCBI |  | EF155408 | NCBI |  | DQ064507 | NCBI |  | JQ901657 | NCBI |
| DQ064549 | NCBI |  | CY023574 | NCBI |  | CY023855 | NCBI |  | EF154974 | NCBI |
| KF746832 | NCBI |  | CY023470 | NCBI |  | CY023871 | fludb |  | KF259135 | NCBI |
| EU914200 | NCBI |  | EF155405 | NCBI |  | KF746826 | NCBI |  | EF154977 | NCBI |
| EU914195 | NCBI |  | EU502899 | NCBI |  | CY077083 | NCBI |  | JQ901651 | NCBI |
| DQ064559 | NCBI |  | KF260589 | NCBI |  | AF508683 | NCBI |  | JQ901653 | NCBI |
| EF070740 | NCBI |  | EF155401 | NCBI |  | CY024695 | NCBI |  | EF154979 | NCBI |
| JF916718 | NCBI |  | DQ226168 | NCBI |  | CY023879 | NCBI |  | GU053179 | NCBI |
| JQ639783 | NCBI |  | KF260580 | NCBI |  | DQ064492 | NCBI |  | JQ901648 | NCBI |
| CY024637 | NCBI |  | JF795108 | NCBI |  | DQ064491 | NCBI |  | KF259136 | NCBI |
| CY005512 | NCBI |  | CY075052 | NCBI |  | EU532063 | NCBI |  | AB080226 | NCBI |
| DQ064548 | NCBI |  | KF260609 | NCBI |  | DQ226155 | NCBI |  | JQ901649 | fludb |
| KF142478 | NCBI |  | CY024334 | NCBI |  | CY023903 | NCBI |  | JQ901655 | fludb |
| AF156434 | NCBI |  | AY664778 | fludb |  | DQ064494 | NCBI |  | AY206676 | NCBI |
| EU086263 | NCBI |  | DQ064536 | NCBI |  | EU502908 | NCBI |  | KF259132 | NCBI |
| CY023725 | NCBI |  | DQ064518 | NCBI |  | AF222820 | NCBI |  | AF156378 | NCBI |
| KF971994 | NCBI |  | KF260588 | NCBI |  | AF222821 | fludb |  | JQ901652 | fludb |
| KC162238 | NCBI |  | GQ373049 | NCBI |  | DQ064493 | NCBI |  | KF188316 | NCBI |
| DQ485213 | NCBI |  | CY023630 | NCBI |  | EF155274 | NCBI |  | CY055148 | NCBI |
| KF972114 | NCBI |  | EU081870 | NCBI |  | DQ064498 | NCBI |  | CY024552 | NCBI |
| KF972090 | NCBI |  | CY023774 | NCBI |  | CY024119 | NCBI |  | KF259081 | NCBI |
| KF971954 | NCBI |  | KF260586 | fludb |  | EF155272 | NCBI |  | KF259131 | fludb |
| KF972034 | NCBI |  | CY005505 | NCBI |  | EF155291 | NCBI |  | KF188335 | NCBI |
| KF972042 | NCBI |  | EU086261 | NCBI |  | AF523447 | NCBI |  | KF259130 | NCBI |
| KF972018 | NCBI |  | CY023374 | NCBI |  | GU053183 | NCBI |  | KF259133 | NCBI |
| KF972066 | NCBI |  | KF260583 | NCBI |  | EF155278 | NCBI |  | JQ901650 | NCBI |
| KF971978 | NCBI |  | KF260585 | NCBI |  | CY023095 | NCBI |  | EF154975 | NCBI |
| KF972074 | NCBI |  | KF260593 | NCBI |  | DQ064501 | NCBI |  | KF188323 | NCBI |
| KF972098 | NCBI |  | GQ373034 | NCBI |  | EF155279 | NCBI |  | JQ901658 | NCBI |
| DQ485205 | NCBI |  | CY005517 | fludb |  | CY024207 | NCBI |  | JQ901654 | fludb |
| EF154862 | NCBI |  | KF260560 | NCBI |  | EF155282 | NCBI |  | JQ901656 | NCBI |
| KF971946 | NCBI |  | KF260607 | NCBI |  | EF155273 | NCBI |  | AY281745 | NCBI |
| CY024237 | NCBI |  | AY664775 | NCBI |  | EF155287 | NCBI |  | KF688983 | NCBI |
| KF972058 | NCBI |  | CY075028 | NCBI |  | AJ404636 | NCBI |  | AF508566 | NCBI |
| KF972002 | NCBI |  | CY023438 | NCBI |  | EF155286 | NCBI |  | AF461526 | NCBI |
| EF612749 | NCBI |  | DQ981552 | NCBI |  | EF155277 | NCBI |  | DQ064366 | NCBI |
| KF972106 | NCBI |  | CY024358 | NCBI |  | EF155295 | NCBI |  | DQ064373 | NCBI |
| KF972010 | NCBI |  | GQ373044 | NCBI |  | AF156449 | NCBI |  | AF508564 | NCBI |
| KF972026 | NCBI |  | CY023614 | NCBI |  | AF257191 | NCBI |  | FJ190131 | NCBI |
| JX437691 | NCBI |  | DQ997510 | NCBI |  | EF155292 | NCBI |  | AY513715 | NCBI |
| KF972082 | NCBI |  | KF260590 | NCBI |  | DQ064499 | NCBI |  | FJ190112 | NCBI |
| KF971962 | NCBI |  | AF156422 | NCBI |  | AF523446 | NCBI |  | AF536693 | NCBI |
| KF972050 | NCBI |  | EU516318 | NCBI |  | AY043028 | NCBI |  | FJ190130 | NCBI |
| KF971986 | NCBI |  | GQ373048 | NCBI |  | EF155289 | fludb |  | FJ190129 | NCBI |
| KF971970 | NCBI |  | CY023654 | NCBI |  | EF155288 | NCBI |  | AF536689 | NCBI |
| CY024669 | NCBI |  | KF260600 | fludb |  | JF795129 | NCBI |  | DQ064376 | NCBI |
| CY024725 | NCBI |  | GQ373043 | NCBI |  | EF155301 | NCBI |  | DQ064377 | NCBI |
| AF523476 | NCBI |  | EF070739 | NCBI |  | CY024559 | NCBI |  | AF384557 | NCBI |
| AF523473 | NCBI |  | CY023662 | NCBI |  | AF508678 | NCBI |  | JF916713 | NCBI |
| CY024605 | NCBI |  | CY023486 | NCBI |  | HQ117885 | fludb |  | GQ477293 | NCBI |
| AF523477 | NCBI |  | KF260619 | fludb |  | EF155283 | NCBI |  | FJ190150 | NCBI |
| CY023309 | NCBI |  | DQ465398 | NCBI |  | KF972068 | fludb |  | FJ190132 | NCBI |
| EU086244 | NCBI |  | CY024518 | fludb |  | KF972092 | NCBI |  | AF536691 | NCBI |
| CY023973 | NCBI |  | DQ064534 | NCBI |  | KF971972 | NCBI |  | DQ064374 | NCBI |
| CY024621 | NCBI |  | CY023734 | NCBI |  | KF972100 | fludb |  | FJ190117 | NCBI |
| CY024381 | NCBI |  | CY023638 | NCBI |  | KF972044 | NCBI |  | AF536692 | NCBI |
| CY024717 | NCBI |  | GQ373041 | NCBI |  | EU516319 | NCBI |  | JF795136 | NCBI |
| CY024581 | NCBI |  | KF260615 | NCBI |  | KF971996 | NCBI |  | HM008899 | NCBI |
| CY023133 | NCBI |  | DQ981560 | NCBI |  | JX437688 | NCBI |  | JF916721 | NCBI |
| CY024565 | NCBI |  | KF260575 | NCBI |  | KF972116 | fludb |  | AF461527 | NCBI |
| AF523479 | NCBI |  | AF508631 | NCBI |  | KF971956 | NCBI |  | FJ231865 | NCBI |
| CY024645 | NCBI |  | CY023350 | NCBI |  | KF971988 | NCBI |  | FJ190126 | NCBI |
| EF154838 | NCBI |  | KF260561 | NCBI |  | KF972020 | NCBI |  | FJ793284 | NCBI |
| CY024573 | NCBI |  | KF260596 | NCBI |  | KF972108 | fludb |  | JQ639778 | NCBI |
| CY023925 | NCBI |  | AF222818 | NCBI |  | KF972036 | NCBI |  | DQ064360 | NCBI |
| GQ477276 | NCBI |  | AF508636 | NCBI |  | KF972084 | fludb |  | FJ190124 | NCBI |
| CY023933 | NCBI |  | CY024326 | NCBI |  | KF972028 | fludb |  | AY594194 | NCBI |
| CY023941 | NCBI |  | AY180873 | fludb |  | KF972012 | NCBI |  | FJ190111 | NCBI |
| CY023413 | fludb |  | EU502907 | NCBI |  | KF971948 | NCBI |  | EU753322 | NCBI |
| JF795107 | NCBI |  | DQ064519 | NCBI |  | KF971980 | fludb |  | KF746810 | fludb |
| EF154861 | NCBI |  | CY024342 | NCBI |  | KF972052 | NCBI |  | DQ997465 | NCBI |
| CY024053 | NCBI |  | CY024414 | fludb |  | KF972076 | NCBI |  | DQ997419 | NCBI |
| CY023197 | NCBI |  | KF746775 | NCBI |  | KF972004 | NCBI |  | DQ064355 | NCBI |
| CY023333 | NCBI |  | AY664784 | NCBI |  | KF972060 | fludb |  | FJ793372 | NCBI |
| EF154837 | NCBI |  | DQ064520 | NCBI |  | KF971964 | NCBI |  | FJ793436 | NCBI |
| CY023165 | NCBI |  | KF260601 | NCBI |  | AF523452 | NCBI |  | DQ681216 | NCBI |
| GQ477275 | NCBI |  | CY023462 | NCBI |  | CY024733 | NCBI |  | FJ384751 | NCBI |
| CY024677 | NCBI |  | KF260578 | NCBI |  | JF795109 | NCBI |  | DQ997474 | NCBI |
| CY023173 | NCBI |  | CY023678 | NCBI |  | CY023455 | NCBI |  | KF142481 | fludb |
| CY023125 | NCBI |  | GQ373035 | NCBI |  | CY005528 | NCBI |  | AF461517 | NCBI |
| CY023109 | NCBI |  | KF260572 | NCBI |  | CY024487 | fludb |  | DQ997437 | NCBI |
| EF154871 | NCBI |  | AF222819 | fludb |  | CY023327 | NCBI |  | AF508571 | NCBI |
| CY024613 | NCBI |  | KF260581 | NCBI |  | EF155280 | NCBI |  | DQ064379 | NCBI |
| CY024589 | NCBI |  | EF155354 | NCBI |  | CY023415 | NCBI |  | DQ681203 | NCBI |
| CY023453 | NCBI |  | KF746760 | NCBI |  | CY005504 | NCBI |  | EU365371 | NCBI |
| CY023445 | NCBI |  | DQ064521 | NCBI |  | CY023151 | NCBI |  | AY364228 | NCBI |
| CY024709 | NCBI |  | CY023430 | NCBI |  | CY023303 | NCBI |  | DQ997460 | NCBI |
| CY024485 | fludb |  | KF260568 | NCBI |  | CY023311 | fludb |  | FJ793404 | fludb |
| CY024597 | NCBI |  | CY024430 | fludb |  | EF155284 | NCBI |  | AF508568 | NCBI |
| CY023949 | NCBI |  | DQ064538 | NCBI |  | CY023335 | NCBI |  | DQ681207 | NCBI |
| CY024477 | NCBI |  | KF260571 | NCBI |  | CY024295 | NCBI |  | KF835984 | NCBI |
| CY023205 | NCBI |  | AF523428 | NCBI |  | AF523453 | NCBI |  | AF461509 | NCBI |
| AF523474 | NCBI |  | CY023582 | NCBI |  | CY024095 | NCBI |  | AF461532 | NCBI |
| CY023181 | NCBI |  | KF260565 | NCBI |  | CY024479 | fludb |  | DQ681221 | NCBI |
| CY023149 | NCBI |  | CY023342 | NCBI |  | AF523451 | NCBI |  | DQ997448 | NCBI |
| CY023117 | NCBI |  | AF508639 | NCBI |  | EF155281 | NCBI |  | FJ793292 | NCBI |
| CY024701 | NCBI |  | CY023494 | NCBI |  | EU081869 | NCBI |  | DQ997505 | NCBI |
| CY024069 | NCBI |  | JF795116 | NCBI |  | CY023407 | NCBI |  | FJ190122 | NCBI |
| CY005506 | NCBI |  | KF260584 | NCBI |  | GQ477285 | NCBI |  | AF461515 | NCBI |
| CY005524 | fludb |  | AF508632 | NCBI |  | AF523456 | NCBI |  | AF461522 | NCBI |
| CY023325 | NCBI |  | KF260559 | NCBI |  | EF155285 | NCBI |  | AF461511 | NCBI |
| CY023405 | NCBI |  | CY023670 | NCBI |  | AF523449 | NCBI |  | DQ997451 | NCBI |
| CY023917 | NCBI |  | CY024350 | fludb |  | CY024143 | NCBI |  | DQ997428 | NCBI |
| CY023101 | NCBI |  | DQ064530 | NCBI |  | CY024567 | NCBI |  | DQ997497 | NCBI |
| AF523475 | NCBI |  | EF155406 | NCBI |  | CY024623 | NCBI |  | EF154943 | NCBI |
| EF154842 | NCBI |  | DQ064537 | NCBI |  | CY023135 | NCBI |  | CY023360 | NCBI |
| CY024173 | NCBI |  | CY023550 | NCBI |  | GQ477284 | NCBI |  | AY043019 | NCBI |
| CY005530 | NCBI |  | HQ425332 | NCBI |  | AF523455 | NCBI |  | EF154940 | NCBI |
| CY024685 | NCBI |  | DQ226167 | NCBI |  | CY023167 | NCBI |  | EF154920 | NCBI |
| CY023141 | NCBI |  | DQ064527 | NCBI |  | CY024655 | NCBI |  | AF523380 | NCBI |
| EF154843 | NCBI |  | KF260604 | NCBI |  | CY023103 | NCBI |  | CY023272 | NCBI |
| CY024141 | NCBI |  | AF156419 | NCBI |  | AY180665 | NCBI |  | CY023712 | NCBI |
| CY023157 | NCBI |  | CY024038 | NCBI |  | CY024071 | NCBI |  | CY024120 | NCBI |
| CY023909 | NCBI |  | CY024510 | NCBI |  | EF155290 | NCBI |  | AF461531 | NCBI |
| CY024469 | NCBI |  | DQ064517 | NCBI |  | EU086242 | NCBI |  | CY024312 | NCBI |
| CY024653 | NCBI |  | CY023190 | NCBI |  | CY023967 | NCBI |  | EF154932 | NCBI |
| CY024661 | NCBI |  | KF260574 | NCBI |  | CY024663 | NCBI |  | EF154919 | NCBI |
| CY023965 | NCBI |  | KF260605 | fludb |  | CY024383 | NCBI |  | EF154927 | NCBI |
| CY023301 | NCBI |  | EF155404 | NCBI |  | CY023143 | NCBI |  | CY024296 | NCBI |
| CY024093 | NCBI |  | AF508633 | NCBI |  | EF155299 | NCBI |  | GQ477291 | NCBI |
| KF260848 | NCBI |  | JF916703 | NCBI |  | CY023951 | NCBI |  | KF313568 | NCBI |
| KF260836 | NCBI |  | KF260606 | NCBI |  | CY024727 | NCBI |  | EF154945 | fludb |
| KF367729 | NCBI |  | DQ064535 | NCBI |  | CY023919 | NCBI |  | CY024072 | NCBI |
| KC821029 | NCBI |  | AF523427 | NCBI |  | CY023927 | NCBI |  | AF156380 | NCBI |
| GQ373017 | NCBI |  | CY023542 | NCBI |  | EF155275 | NCBI |  | EF154933 | fludb |
| CY024341 | NCBI |  | DQ226165 | NCBI |  | CY024615 | NCBI |  | EF154910 | NCBI |
| JF519813 | NCBI |  | CY024406 | fludb |  | CY023447 | NCBI |  | EF154931 | NCBI |
| KF260840 | NCBI |  | JQ639776 | NCBI |  | EF155276 | NCBI |  | DQ226108 | NCBI |
| EU086262 | NCBI |  | DQ064531 | NCBI |  | CY023159 | NCBI |  | EF154944 | NCBI |
| KF746788 | NCBI |  | DQ064532 | NCBI |  | CY023183 | NCBI |  | CY023536 | NCBI |
| CY024437 | NCBI |  | CY023830 | NCBI |  | CY024591 | NCBI |  | CY023091 | NCBI |
| KF746840 | fludb |  | JN869515 | NCBI |  | CY024671 | NCBI |  | CY024080 | NCBI |
| CY023237 | NCBI |  | CY023646 | NCBI |  | CY023199 | NCBI |  | CY024208 | NCBI |
| EU086332 | NCBI |  | JQ639784 | NCBI |  | CY023127 | NCBI |  | CY023800 | NCBI |
| EU086333 | NCBI |  | CY024662 | NCBI |  | CY023111 | NCBI |  | CY023624 | NCBI |
| KF260803 | NCBI |  | KF260582 | NCBI |  | CY024679 | NCBI |  | CY023888 | NCBI |
| KF260846 | fludb |  | DQ997449 | NCBI |  | CY023975 | NCBI |  | KF313566 | NCBI |
| CY023685 | NCBI |  | AF508630 | NCBI |  | CY024647 | NCBI |  | EF154907 | NCBI |
| EF154895 | NCBI |  | GQ373046* | fludb |  | AF523450 | NCBI |  | AY664674 | NCBI |
| KF260864 | NCBI |  | CY024390 | NCBI |  | CY023119 | NCBI |  | EF154935 | NCBI |
| KF260857 | NCBI |  | KF746825 | NCBI |  | CY024575 | NCBI |  | EF154925 | NCBI |
| KC767264 | NCBI |  | KF260577 | NCBI |  | CY023175 | NCBI |  | CY024064 | NCBI |
| GU474563 | NCBI |  | KF260599 | NCBI |  | CY023207 | NCBI |  | CY023984 | NCBI |
| GQ373019 | NCBI |  | KF260598 | NCBI |  | CY024607 | NCBI |  | CY024152 | NCBI |
| CY023437 | NCBI |  | DQ485206 | NCBI |  | CY024583 | NCBI |  | EF154976 | NCBI |
| GU474547 | NCBI |  | DQ064539 | NCBI |  | CY024719 | NCBI |  | CY024504 | NCBI |
| CY023565 | NCBI |  | AY664777 | NCBI |  | CY024471 | NCBI |  | EF154951 | NCBI |
| KF260815 | NCBI |  | DQ981536 | NCBI |  | CY024711 | NCBI |  | CY024536 | NCBI |
| AY253750 | NCBI |  | KF746808 | NCBI |  | CY023943 | NCBI |  | CY024376 | NCBI |
| KF260828 | NCBI |  | CY023566 | NCBI |  | CY024687 | NCBI |  | CY024184 | NCBI |
| FJ384748 | NCBI |  | CY075044 | NCBI |  | CY024599 | NCBI |  | EF154978 | NCBI |
| AY664798 | NCBI |  | DQ981568 | NCBI |  | AF523448 | NCBI |  | CY024272 | NCBI |
| KF746864 | NCBI |  | KF260562 | NCBI |  | CY024703 | NCBI |  | CY024088 | NCBI |
| KF260847 | NCBI |  | AY664780 | NCBI |  | KC162236 | NCBI |  | CY024464 | NCBI |
| CY023629 | NCBI |  | EU882862 | NCBI |  | CY024015 | NCBI |  | DQ226107 | NCBI |
| GQ373027 | NCBI |  | KF260564 | NCBI |  | CY023271 | NCBI |  | CY024528 | NCBI |
| KF260841 | NCBI |  | KC768057 | NCBI |  | EF155300 | NCBI |  | CY024544 | NCBI |
| GQ335476 | NCBI |  | EF155402 | NCBI |  | EF155327 | NCBI |  | CY024136 | NCBI |
| CY023365 | NCBI |  | KF260566 | NCBI |  | CY024527 | NCBI |  | CY024456 | NCBI |
| JF519812 | NCBI |  | CY024638 | NCBI |  | EF155320 | NCBI |  | CY024496 | NCBI |
| CY023749 | NCBI |  | DQ064533 | NCBI |  | EF155326 | NCBI |  | CY024288 | NCBI |
| AF523472 | NCBI |  | CY023558 | NCBI |  | KF260335 | NCBI |  | EF154957 | NCBI |
| FJ793289 | NCBI |  | KF260602 | NCBI |  | EF155314 | NCBI |  | CY024192 | NCBI |
| KF746848 | NCBI |  | CY023686 | NCBI |  | EF155298 | NCBI |  | CY024400 | NCBI |
| GQ477278 | NCBI |  | KF367730 | NCBI |  | CY023799 | NCBI |  | CY024424 | fludb |
| CY023821 | NCBI |  | GQ373045 | NCBI |  | EF155294 | fludb |  | CY023808 | NCBI |
| AY664802 | NCBI |  | CY024398 | NCBI |  | EF155297 | NCBI |  | CY023384 | NCBI |
| HM590767 | fludb |  | EF155368 | NCBI |  | CY023711 | NCBI |  | CY024048 | NCBI |
| KF260863 | fludb |  | CY023958 | NCBI |  | EF155340 | NCBI |  | CY023280 | NCBI |
| GQ373023 | NCBI |  | EF155391 | NCBI |  | EF155321 | NCBI |  | CY024216 | NCBI |
| CY023789 | fludb |  | EF155409 | NCBI |  | EF155313 | NCBI |  | CY024160 | NCBI |
| CY023669 | NCBI |  | CY023894 | NCBI |  | AF156448 | NCBI |  | CY024224 | NCBI |
| CY023477 | NCBI |  | AF508634 | NCBI |  | EF155306 | NCBI |  | CY024368 | NCBI |
| KF260817 | NCBI |  | CY024302 | NCBI |  | EF155303 | NCBI |  | CY024112 | NCBI |
| CY023701 | NCBI |  | CY024230 | NCBI |  | CY023983 | NCBI |  | CY024000 | NCBI |
| EF154893 | NCBI |  | EF155417 | NCBI |  | EF155305 | NCBI |  | DQ226106 | NCBI |
| KF260809 | NCBI |  | AF156421 | NCBI |  | CY023623 | fludb |  | EF154946 | NCBI |
| CY023773 | fludb |  | EF155379 | NCBI |  | EF155302 | NCBI |  | CY024248 | NCBI |
| KF260832 | fludb |  | DQ485214 | NCBI |  | AF523454 | NCBI |  | CY024264 | NCBI |
| KF260833 | NCBI |  | DQ064516 | NCBI |  | EF155317 | NCBI |  | CY024256 | NCBI |
| KF260822 | NCBI |  | EF155380 | NCBI |  | EF155323 | NCBI |  | CY024448 | NCBI |
| CY023245 | NCBI |  | CY024270 | NCBI |  | CY023887 | NCBI |  | CY024280 | NCBI |
| CY024509 | fludb |  | CY023806 | NCBI |  | CY023535 | fludb |  | CY024304 | NCBI |
| KF260804 | NCBI |  | CY024134 | NCBI |  | EF155325 | NCBI |  | CY024320 | NCBI |
| CY023341 | NCBI |  | EF155347 | NCBI |  | EF155296 | NCBI |  | CY024232 | NCBI |
| CY024037 | NCBI |  | CY023846 | NCBI |  | EF155304 | NCBI |  | CY023288 | NCBI |
| CY023829 | NCBI |  | EF155360 | NCBI |  | EF155307 | NCBI |  | AB432938 | NCBI |
| DQ997418 | NCBI |  | CY023710 | NCBI |  | CY024551 | NCBI |  | AB080227 | NCBI |
| CY024325 | NCBI |  | EF155377 | fludb |  | CY023359 | NCBI |  | AF222810 | NCBI |
| DQ681211 | NCBI |  | EF155413 | NCBI |  | EF155342 | NCBI |  | FJ190148 | NCBI |
| GU474587 | NCBI |  | AY043029 | NCBI |  | EF155293 | NCBI |  | AF156373 | NCBI |
| KF260819 | NCBI |  | EF155356 | NCBI |  | EF155324 | NCBI |  | AF156374 | NCBI |
| GU474595 | NCBI |  | EF155359 | NCBI |  | CY024191 | fludb |  | DQ064380 | NCBI |
| CY055153 | NCBI |  | EF155370 | NCBI |  | DQ226151 | NCBI |  | AF156375 | NCBI |
| KF260813 | NCBI |  | JQ901621 | NCBI |  | CY023287 | NCBI |  | DQ064362 | NCBI |
| EU086282 | NCBI |  | CY024254 | fludb |  | EF155341 | NCBI |  | CY098089 | NCBI |
| CY023581 | NCBI |  | EF155393 | NCBI |  | EF155330 | NCBI |  | FJ793364 | NCBI |
| EF154890 | NCBI |  | EF155369 | NCBI |  | CY024111 | NCBI |  | KF188389 | NCBI |
| AF523471 | NCBI |  | CY023090 | NCBI |  | CY024263 | NCBI |  | HM773435 | NCBI |
| JF795121 | NCBI |  | EF155396 | NCBI |  | EF155316 | NCBI |  | EU753298 | NCBI |
| KF260849 | NCBI |  | CY023358 | NCBI |  | EF155334 | fludb |  | CY023752 | NCBI |
| CY024349 | NCBI |  | EF155346 | NCBI |  | CY023319 | NCBI |  | GQ477290 | NCBI |
| CY023485 | NCBI |  | CY024374 | NCBI |  | CY023807 | NCBI |  | CY024608 | NCBI |
| CY024165 | fludb |  | JQ901705 | NCBI |  | CY024535 | NCBI |  | AB634251 | NCBI |
| CY023989 | NCBI |  | EF155367 | NCBI |  | KF260379 | NCBI |  | JN381628 | NCBI |
| CY024333 | NCBI |  | CY023862 | NCBI |  | CY055147 | NCBI |  | AF461525 | NCBI |
| GQ202047 | NCBI |  | EF155363 | NCBI |  | CY024215 | NCBI |  | CY006018 | NCBI |
| CY023261 | NCBI |  | CY024446 | NCBI |  | CY024159 | NCBI |  | KF746785 | NCBI |
| DQ681220 | NCBI |  | EF155384 | NCBI |  | JQ901646 | fludb |  | EU753314 | NCBI |
| GQ335504 | NCBI |  | CY024062 | NCBI |  | CY024231 | NCBI |  | EU753290 | fludb |
| CY023493 | NCBI |  | CY024182 | NCBI |  | JQ901647 | NCBI |  | CY024592 | NCBI |
| CY023517 | NCBI |  | CY024286 | NCBI |  | DQ226150 | NCBI |  | FJ793396 | NCBI |
| CY023389 | NCBI |  | CY023622 | fludb |  | EF155332 | NCBI |  | AB634252 | NCBI |
| GQ373024 | NCBI |  | CY023534 | NCBI |  | CY023999 | NCBI |  | AY083841 | NCBI |
| DQ997467 | NCBI |  | KF260623 | NCBI |  | JQ901637 | fludb |  | KF188282 | fludb |
| CY023573 | fludb |  | AF523430 | NCBI |  | EF155344 | NCBI |  | AF461523 | NCBI |
| GQ335496 | NCBI |  | AF523443 | NCBI |  | CY024543 | fludb |  | JQ710462 | NCBI |
| KC821030 | NCBI |  | EF155350 | NCBI |  | EF155339 | NCBI |  | HQ326722 | NCBI |
| AY664792 | NCBI |  | EF155388 | NCBI |  | JQ901640 | fludb |  | HM773436 | NCBI |
| CY023693 | NCBI |  | EF155365 | NCBI |  | CY023279 | NCBI |  | HQ398361 | NCBI |
| KC821031 | NCBI |  | EF155390 | NCBI |  | JQ901643 | fludb |  | AF508569 | NCBI |
| CY023501 | NCBI |  | CY024542 | NCBI |  | JQ901638 | fludb |  | EU753330 | NCBI |
| GU474579 | NCBI |  | DQ064525 | NCBI |  | CY024271 | NCBI |  | FJ793380 | NCBI |
| CY023213 | NCBI |  | AF523431 | NCBI |  | JQ901641 | fludb |  | AY083840 | NCBI |
| KF260811 | NCBI |  | DQ226161 | NCBI |  | JQ901645 | fludb |  | CY024568 | NCBI |
| FJ793281 | fludb |  | EF155397 | NCBI |  | CY024151 | NCBI |  | AF461524 | NCBI |
| JF795115 | NCBI |  | EF155352 | NCBI |  | EF155343 | NCBI |  | FJ793316 | NCBI |
| KF260859 | NCBI |  | EF155366 | NCBI |  | JQ901639 | fludb |  | GQ477294 | NCBI |
| HM751183 | NCBI |  | EF155392 | NCBI |  | JQ901642 | NCBI |  | AF536698 | NCBI |
| KF260825 | NCBI |  | CY023286 | NCBI |  | JQ901644 | NCBI |  | AF461510 | NCBI |
| DQ064567 | NCBI |  | CY024190 | NCBI |  | CY024135 | NCBI |  | DQ064378 | NCBI |
| KF260834 | NCBI |  | EF155394 | fludb |  | DQ226152 | NCBI |  | EU753306 | NCBI |
| FJ547481 | NCBI |  | CY023998 | NCBI |  | CY024047 | NCBI |  | CY023768 | NCBI |
| CY024197 | fludb |  | CY024158 | NCBI |  | CY024287 | NCBI |  | DQ064361 | NCBI |
| CY023509 | NCBI |  | DQ064524 | NCBI |  | CY024447 | NCBI |  | CY023848 | NCBI |
| KF260806 | NCBI |  | DQ226163 | NCBI |  | CY024375 | fludb |  | DQ981610 | NCBI |
| EU516309 | NCBI |  | EF155389 | NCBI |  | CY024423 | NCBI |  | AF461528 | NCBI |
| GQ335486* | fludb |  | EF155375 | NCBI |  | EF155312 | NCBI |  | CY023864 | NCBI |
| CY023733 | NCBI |  | CY023318 | NCBI |  | CY024367 | NCBI |  | DQ981578 | NCBI |
| JN653570 | NCBI |  | EF155410 | fludb |  | CY024127 | NCBI |  | CY023856 | NCBI |
| DQ981615 | NCBI |  | CY023982 | NCBI |  | CY024311 | NCBI |  | FJ793340 | NCBI |
| GQ202061 | NCBI |  | CY024366 | NCBI |  | EF155315 | NCBI |  | CY023880 | NCBI |
| CY023765 | NCBI |  | CY023422 | NCBI |  | CY024255 | fludb |  | JN381627 | NCBI |
| KF260839 | NCBI |  | AF258816 | NCBI |  | EF155311 | NCBI |  | DQ981626 | NCBI |
| FJ547486 | NCBI |  | EF155416 | NCBI |  | DQ226153 | NCBI |  | CY023896 | NCBI |
| GU474571 | NCBI |  | EF155361 | NCBI |  | CY024399 | NCBI |  | EU516304 | NCBI |
| EU365368 | NCBI |  | CY023270 | NCBI |  | CY024319 | fludb |  | HQ117886 | NCBI |
| KF715225 | fludb |  | EF155383 | NCBI |  | CY024463 | NCBI |  | DQ064363 | NCBI |
| GQ373020 | NCBI |  | CY024454 | NCBI |  | CY024455 | NCBI |  | DQ981602 | NCBI |
| KF715233 | NCBI |  | EF155378 | NCBI |  | CY024239 | NCBI |  | FJ793324 | NCBI |
| KF260850 | NCBI |  | EU086314 | NCBI |  | CY023295 | NCBI |  | AF461529 | NCBI |
| KF260852 | fludb |  | CY024150 | NCBI |  | EF155310 | NCBI |  | FJ190125 | NCBI |
| CY023373 | NCBI |  | CY024502 | NCBI |  | CY023215 | NCBI |  | FJ793356 | NCBI |
| DQ226180 | NCBI |  | JQ901623 | fludb |  | CY023959 | NCBI |  | AY294658 | NCBI |
| HM998911 | NCBI |  | CY023094 | NCBI |  | CY024279 | fludb |  | DQ064364 | NCBI |
| KF260843 | NCBI |  | CY024526 | NCBI |  | CY024063 | NCBI |  | CY023872 | NCBI |
| KF260824 | NCBI |  | EF155355 | NCBI |  | CY023223 | NCBI |  | DQ227352 | NCBI |
| DQ997504 | NCBI |  | KF260579 | NCBI |  | CY024087 | NCBI |  | GQ477295 | NCBI |
| JF795043 | NCBI |  | CY024118 | NCBI |  | CY024247 | NCBI |  | FJ231864 | NCBI |
| GU121379 | NCBI |  | CY023382 | NCBI |  | CY024503 | NCBI |  | GQ477289 | NCBI |
| DQ064556 | NCBI |  | DQ064528 | NCBI |  | CY024183 | NCBI |  | FJ793348 | NCBI |
| GQ202053 | NCBI |  | CY024110 | NCBI |  | EF155308 | NCBI |  | CY023904 | NCBI |
| JF916702 | NCBI |  | EF155414 | NCBI |  | EF155318 | fludb |  | DQ981618 | NCBI |
| KF260805 | NCBI |  | AF156417 | NCBI |  | EF155309 | NCBI |  | AY036880 | NCBI |
| KF746816 | NCBI |  | CY055146 | NCBI |  | CY024079 | NCBI |  | EF612742 | NCBI |
| CY023429 | NCBI |  | EF155357 | NCBI |  | CY024223 | NCBI |  | HM590770 | NCBI |
| DQ997476 | NCBI |  | EF155376 | NCBI |  | CY024495 | NCBI |  | EU086303 | NCBI |
| KF260845 | NCBI |  | CY024278 | NCBI |  | CY024303 | NCBI |  | GU722364 | NCBI |
| CY023837 | NCBI |  | AF523429 | NCBI |  | CY023831 | NCBI |  | CY023728 | NCBI |
| FJ534543 | NCBI |  | CY023854 | NCBI |  | GQ373058 | NCBI |  | GU722359 | NCBI |
| AY664794 | fludb |  | AF156418 | NCBI |  | CY023231 | NCBI |  | GU722368 | NCBI |
| CY023781 | fludb |  | CY024558 | NCBI |  | CY023239 | NCBI |  | KC817011 | NCBI |
| KF260858 | NCBI |  | DQ226164 | NCBI |  | EF155329 | NCBI |  | EU086283 | NCBI |
| FJ793369 | NCBI |  | CY023902 | NCBI |  | CY024007 | NCBI |  | KC817008 | NCBI |
| HM590759 | fludb |  | CY024126 | NCBI |  | CY024023 | NCBI |  | CY023696 | NCBI |
| GQ373026 | NCBI |  | CY024310 | NCBI |  | CY024103 | NCBI |  | GU722369 | NCBI |
| KF746774 | NCBI |  | JQ901624 | fludb |  | EF155319 | NCBI |  | HM590762 | NCBI |
| CY023549 | fludb |  | EF155398 | NCBI |  | GQ373067 | NCBI |  | KC817013 | NCBI |
| GQ373033 | NCBI |  | EF155415 | NCBI |  | GQ373062 | NCBI |  | GU722361 | NCBI |
| GQ202041 | fludb |  | CY024462 | NCBI |  | GQ373066 | NCBI |  | KC817012 | NCBI |
| CY024101 | NCBI |  | HQ117884 | fludb |  | CY023263 | NCBI |  | GU722363 | NCBI |
| JF519816 | NCBI |  | JQ901620 | fludb |  | CY023991 | NCBI |  | EU086266 | NCBI |
| EU086281 | NCBI |  | JQ901703 | NCBI |  | GQ373061 | NCBI |  | EU086319 | NCBI |
| CY023589 | NCBI |  | GU053184 | NCBI |  | EU086258 | NCBI |  | KC817009 | NCBI |
| JF519811 | NCBI |  | KF188314 | NCBI |  | CY023783 | NCBI |  | JN381629 | NCBI |
| KF260831 | NCBI |  | CY023878 | NCBI |  | CY024415 | NCBI |  | CY023704 | NCBI |
| KF260810 | NCBI |  | CY023870 | NCBI |  | CY024407 | fludb |  | EU086302 | NCBI |
| GQ373032 | NCBI |  | JF795128 | NCBI |  | CY023775 | NCBI |  | GU121382 | NCBI |
| KF746807 | NCBI |  | CY024206 | NCBI |  | GQ373051 | NCBI |  | EU086234 | NCBI |
| KF260821 | NCBI |  | CY024494 | NCBI |  | CY024167 | fludb |  | CY023720 | NCBI |
| FJ492970 | NCBI |  | CY024078 | NCBI |  | GQ373052 | NCBI |  | KC817010 | NCBI |
| JN653571 | NCBI |  | JQ901622 | fludb |  | CY023743 | NCBI |  | EU086318 | NCBI |
| EU086316 | NCBI |  | EF155386 | NCBI |  | CY023391 | NCBI |  | EU086226 | NCBI |
| DQ226179 | NCBI |  | CY023798 | fludb |  | CY023791 | fludb |  | EU086265 | NCBI |
| CY023757 | NCBI |  | DQ485222 | NCBI |  | CY024199 | fludb |  | EU086264 | NCBI |
| CY023605 | fludb |  | EF155362 | NCBI |  | CY024391 | fludb |  | EU086246 | NCBI |
| CY024005 | NCBI |  | EF155358 | NCBI |  | CY024175 | fludb |  | EU304455 | NCBI |
| KF260844 | NCBI |  | EF155399 | NCBI |  | CY023247 | NCBI |  | AY043015 | NCBI |
| CY023813 | NCBI |  | EF155374 | NCBI |  | GQ373065 | NCBI |  | AF156376 | NCBI |
| CY024405 | fludb |  | EF155381 | NCBI |  | CY023255 | NCBI |  | CY024576 | NCBI |
| CY023677 | NCBI |  | AF156416 | NCBI |  | CY024351 | NCBI |  | EU573940 | NCBI |
| DQ997427 | NCBI |  | CY024238 | NCBI |  | GQ373063* | fludb |  | HM008896 | NCBI |
| GU474603 | NCBI |  | CY024086 | NCBI |  | CY023383 | NCBI |  | AF536690 | NCBI |
| EU086300 | NCBI |  | JQ901706 | fludb |  | GQ373060 | NCBI |  | AF461520 | NCBI |
| GQ477277 | NCBI |  | CY024262 | NCBI |  | CY024335 | NCBI |  | KF746756 | NCBI |
| KF260807 | NCBI |  | JQ901625 | NCBI |  | CY023367 | fludb |  | JF795110 | fludb |
| DQ997450 | NCBI |  | CY024046 | NCBI |  | CY023583 | NCBI |  | EF154915 | NCBI |
| EU086317 | NCBI |  | EF155411 | NCBI |  | CY024327 | NCBI |  | AF461530 | NCBI |
| KF367736 | NCBI |  | CY024422 | NCBI |  | CY024511 | NCBI |  | AF523374 | NCBI |
| CY023525 | NCBI |  | JQ901619 | fludb |  | EF155335 | NCBI |  | EU926627 | fludb |
| DQ981575 | NCBI |  | EF155364 | NCBI |  | CY023343 | NCBI |  | CY024616 | NCBI |
| KF746799 | NCBI |  | JQ901704 | fludb |  | CY024439 | NCBI |  | KF746876 | NCBI |
| EU086233 | NCBI |  | CY024214 | fludb |  | CY023479 | NCBI |  | AF523372 | NCBI |
| KF260837 | NCBI |  | EF155373 | NCBI |  | CY023607 | fludb |  | CY024472 | NCBI |
| KF260818 | NCBI |  | CY024534 | NCBI |  | CY023575 | NCBI |  | KF746835 | NCBI |
| GQ335463 | NCBI |  | EU086298 | NCBI |  | CY023543 | NCBI |  | EU573941 | NCBI |
| CY024517 | fludb |  | DQ064526 | NCBI |  | CY023679 | NCBI |  | DQ064354 | NCBI |
| CY023653 | NCBI |  | EF155385 | NCBI |  | CY023591 | NCBI |  | DQ064370 | NCBI |
| CY023541 | fludb |  | CY024222 | NCBI |  | CY024359 | fludb |  | EF154911 | NCBI |
| KF260851 | NCBI |  | EF155371 | NCBI |  | CY023399 | NCBI |  | AF523375 | NCBI |
| CY024389 | fludb |  | EF155412 | NCBI |  | CY023687 | NCBI |  | HM773441 | NCBI |
| CY023469 | fludb |  | CY023278 | NCBI |  | EF155336 | NCBI |  | EU926625 | fludb |
| CY023597 | NCBI |  | EF155345 | NCBI |  | CY023567 | NCBI |  | CY024672 | NCBI |
| CY023637 | NCBI |  | CY023294 | NCBI |  | CY023471 | fludb |  | CY023976 | NCBI |
| CY023349 | NCBI |  | EU086243 | NCBI |  | CY024431 | NCBI |  | CY024624 | NCBI |
| CY023717 | NCBI |  | EF155387 | fludb |  | CY023615 | NCBI |  | KF746762 | fludb |
| FJ499468 | NCBI |  | EF155395 | NCBI |  | CY023431 | NCBI |  | FJ190115 | NCBI |
| GQ373018 | NCBI |  | EF155400 | NCBI |  | EF155338 | fludb |  | HM773433 | NCBI |
| CY023253 | NCBI |  | CY024246 | NCBI |  | CY023423 | NCBI |  | AF523373 | NCBI |
| KC821032 | NCBI |  | CY024294 | NCBI |  | CY023463 | NCBI |  | AF536695 | NCBI |
| GQ373022 | NCBI |  | AJ404635 | NCBI |  | CY023511 | NCBI |  | CY077084 | NCBI |
| GQ373031 | NCBI |  | EF155351 | NCBI |  | CY023655 | fludb |  | KF313565 | NCBI |
| DQ226178 | NCBI |  | CY024318 | NCBI |  | EF155333 | NCBI |  | EU081864 | NCBI |
| KF260820 | NCBI |  | CY023334 | NCBI |  | CY023351 | NCBI |  | KF313562 | NCBI |
| KF260808 | NCBI |  | CY023118 | NCBI |  | CY023631 | fludb |  | KF746827 | NCBI |
| AY664793 | NCBI |  | CY023110 | NCBI |  | EF155337 | NCBI |  | HM008897 | NCBI |
| DQ997496 | NCBI |  | KF972035 | NCBI |  | CY023551 | NCBI |  | AF461513 | NCBI |
| KF260812 | NCBI |  | CY024686 | NCBI |  | CY023375 | NCBI |  | KF313560 | NCBI |
| EU935070 | NCBI |  | KF972051 | NCBI |  | CY023599 | NCBI |  | EU086284 | NCBI |
| CY023661 | NCBI |  | KF972083 | NCBI |  | EF155328 | NCBI |  | AY594195 | NCBI |
| KF260829 | NCBI |  | CY023926 | NCBI |  | EF155331 | NCBI |  | EU573938 | NCBI |
| EU086299 | NCBI |  | CY005529 | NCBI |  | CY024343 | NCBI |  | DQ485216 | NCBI |
| EF154891 | NCBI |  | CY024670 | NCBI |  | CY023559 | NCBI |  | EU086237 | NCBI |
| KF746792 | NCBI |  | CY023310 | NCBI |  | CY024519 | NCBI |  | AF461516 | NCBI |
| EF154892 | fludb |  | CY024702 | NCBI |  | CY023439 | NCBI |  | FJ190128 | NCBI |
| CY023645 | NCBI |  | CY024566 | NCBI |  | KF260351 | NCBI |  | AF508574 | NCBI |
| CY023397 | NCBI |  | KF971971 | NCBI |  | CY023527 | NCBI |  | HM773438 | NCBI |
| CY023221 | NCBI |  | CY023158 | NCBI |  | KF260323 | NCBI |  | HM773439 | NCBI |
| KF260853 | NCBI |  | CY023974 | NCBI |  | KF260362 | NCBI |  | KF313563 | NCBI |
| KF260835 | NCBI |  | CY023182 | NCBI |  | KF260325 | NCBI |  | KF313564 | fludb |
| DQ681225 | NCBI |  | CY024710 | NCBI |  | KF260327 | NCBI |  | DQ064358 | NCBI |
| CY024357 | NCBI |  | KF971987 | NCBI |  | CY023495 | NCBI |  | HM773434 | NCBI |
| KF746855 | NCBI |  | CY024014 | NCBI |  | CY023487 | NCBI |  | AF536697 | NCBI |
| CY023557 | fludb |  | KF972019 | NCBI |  | CY023647 | NCBI |  | DQ465400 | NCBI |
| CY024429 | NCBI |  | KF972011 | NCBI |  | KF260322 | NCBI |  | FJ793412 | NCBI |
| DQ997447 | NCBI |  | KF972107 | NCBI |  | KF260337 | NCBI |  | DQ064365 | NCBI |
| KF260816 | NCBI |  | CY024678 | NCBI |  | KF260316 | NCBI |  | AF508565 | NCBI |
| EF154897 | NCBI |  | CY023198 | NCBI |  | KF260346 | NCBI |  | DQ064375 | NCBI |
| KF260838 | NCBI |  | CY024590 | NCBI |  | KF260365 | NCBI |  | AF536694 | NCBI |
| GQ373028 | NCBI |  | EF155353 | NCBI |  | KF260370 | fludb |  | EU926628 | fludb |
| CY023189 | NCBI |  | CY024486 | fludb |  | KF260330 | NCBI |  | JN381626 | NCBI |
| KF746745 | NCBI |  | CY024726 | NCBI |  | CY023519 | NCBI |  | AF536696 | NCBI |
| CY024413 | fludb |  | CY023414 | NCBI |  | KF260319 | NCBI |  | EF070733 | NCBI |
| KF260830 | NCBI |  | CY023150 | NCBI |  | KF260329 | NCBI |  | KF313559 | NCBI |
| KF260826 | NCBI |  | AF523436 | NCBI |  | KF260352 | NCBI |  | DQ226114 | NCBI |
| CY024021 | NCBI |  | KF972099 | NCBI |  | KF260317 | NCBI |  | DQ226109 | NCBI |
| DQ226181 | NCBI |  | EF155349 | NCBI |  | KF746776 | NCBI |  | DQ064369 | NCBI |
| DQ064550 | NCBI |  | KF971947 | NCBI |  | CY023639 | NCBI |  | GU471797 | NCBI |
| DQ997459 | NCBI |  | JX437686 | NCBI |  | CY023663 | fludb |  | EF154968 | NCBI |
| DQ981623 | NCBI |  | AF156420 | NCBI |  | KF260328 | NCBI |  | CY024440 | NCBI |
| DQ997193 | NCBI |  | KF972075 | NCBI |  | KF260320 | NCBI |  | CY024632 | NCBI |
| EF154896 | NCBI |  | KF971979 | NCBI |  | JF916704 | NCBI |  | EU516312 | NCBI |
| DQ681215 | NCBI |  | CY024094 | NCBI |  | JN869516 | NCBI |  | AY664666 | NCBI |
| GQ335512 | NCBI |  | KF972043 | NCBI |  | CY023671 | fludb |  | CY023376 | NCBI |
| GQ373030 | NCBI |  | AF523433 | NCBI |  | KF260324 | NCBI |  | GU471798 | NCBI |
| CY023741 | fludb |  | CY023934 | NCBI |  | KF260326 | NCBI |  | EU502893 | NCBI |
| KF260842 | NCBI |  | KF972091 | fludb |  | JF795123 | NCBI |  | DQ226110 | NCBI |
| KF260827 | NCBI |  | KF971963 | NCBI |  | KF746866 | NCBI |  | DQ485208 | NCBI |
| EF154894 | NCBI |  | CY023446 | NCBI |  | KC768054 | NCBI |  | DQ226115 | NCBI |
| KF260814 | NCBI |  | KF972003 | NCBI |  | KF260318 | NCBI |  | CY023400 | NCBI |
| FJ547482 | NCBI |  | CY024606 | NCBI |  | KF260369 | NCBI |  | CY024640 | NCBI |
| CY023613 | NCBI |  | CY024574 | NCBI |  | KF260315 | NCBI |  | AY664663 | NCBI |
| CY023461 | NCBI |  | CY023174 | NCBI |  | KF260321 | NCBI |  | DQ064367 | NCBI |
| JF795099 | NCBI |  | AF523435 | NCBI |  | JF795117 | NCBI |  | AY664661 | NCBI |
| CY023229 | NCBI |  | EF155382 | NCBI |  | KF260331 | NCBI |  | EU502901 | NCBI |
| JF795075 | NCBI |  | CY023326 | fludb |  | FJ492967 | NCBI |  | DQ064371 | NCBI |
| EU914199 | NCBI |  | CY024070 | NCBI |  | CY023503 | NCBI |  | AY664665 | NCBI |
| KF746744 | NCBI |  | KF971955 | NCBI |  | KF260359 | NCBI |  | CY023960 | NCBI |
| CY023269 | NCBI |  | CY023942 | NCBI |  | KF260340 | NCBI |  | AY664669 | NCBI |
| DQ226175 | NCBI |  | EF155372 | NCBI |  | KF367731 | NCBI |  | AY664662 | NCBI |
| EF154860 | fludb |  | KC162237 | NCBI |  | KF260371 | NCBI |  | JQ639786 | NCBI |
| CY024277 | fludb |  | CY023142 | NCBI |  | KF260344 | NCBI |  | CY023192 | NCBI |
| AY664806 | NCBI |  | CY023206 | NCBI |  | KF260350 | NCBI |  | AY664673 | NCBI |
| CY024365 | fludb |  | KF972027 | fludb |  | KF260353 | NCBI |  | AY664664 | NCBI |
| JQ901630 | NCBI |  | AY180874 | NCBI |  | KF260336 | NCBI |  | AY664660 | NCBI |
| EF154889 | NCBI |  | KF972115 | NCBI |  | KF260339 | NCBI |  | DQ226113 | NCBI |
| EF154835 | NCBI |  | CY024582 | NCBI |  | KF260376 | NCBI |  | AY664672 | NCBI |
| CY024317 | NCBI |  | CY023302 | NCBI |  | KF260357 | fludb |  | AY664668 | NCBI |
| EF154872 | NCBI |  | CY024622 | NCBI |  | CY055155 | NCBI |  | CY024432 | NCBI |
| JF795127 | NCBI |  | CY024598 | fludb |  | KF260360 | fludb |  | GU471884 | NCBI |
| AF508656 | NCBI |  | CY023950 | NCBI |  | KF260361 | fludb |  | CY023432 | NCBI |
| EF154848 | fludb |  | EF155348 | NCBI |  | KF260338 | NCBI |  | EF154963 | NCBI |
| CY024301 | NCBI |  | GQ477281 | NCBI |  | KF260333 | NCBI |  | EF154973 | NCBI |
| HQ117883 | NCBI |  | CY024614 | NCBI |  | KF260347 | NCBI |  | CY024336 | NCBI |
| CY024109 | NCBI |  | CY024478 | NCBI |  | KF260363 | NCBI |  | CY024360 | NCBI |
| CY024285 | fludb |  | CY023454 | NCBI |  | KF260356 | fludb |  | EF154967 | NCBI |
| JQ901633 | NCBI |  | CY023918 | NCBI |  | KF260375 | NCBI |  | CY023504 | NCBI |
| CY023093 | NCBI |  | KF714773 | NCBI |  | KF260343 | NCBI |  | FJ190143 | NCBI |
| CY024245 | fludb |  | KF971995 | NCBI |  | KF260349 | NCBI |  | FJ190147 | NCBI |
| CY023381 | NCBI |  | CY023166 | NCBI |  | KF260342 | NCBI |  | FJ190136 | NCBI |
| AF156430 | NCBI |  | CY023102 | NCBI |  | KF260348 | NCBI |  | FJ190149 | NCBI |
| CY024117 | NCBI |  | CY023502 | NCBI |  | KF260355 | NCBI |  | FJ190121 | NCBI |
| EF154887 | NCBI |  | CY023910 | NCBI |  | KF260345 | NCBI |  | CY023440 | NCBI |
| CY024061 | NCBI |  | AF523434 | NCBI |  | KF260354 | NCBI |  | CY024408 | fludb |
| CY024445 | fludb |  | KF972067 | NCBI |  | KF260358 | NCBI |  | CY023344 | NCBI |
| CY023893 | NCBI |  | KF972059 | NCBI |  | KF260341 | NCBI |  | CY024352 | NCBI |
| CY023901 | NCBI |  | CY023406 | NCBI |  | KF260334 | NCBI |  | FJ190145 | NCBI |
| CY024133 | NCBI |  | CY024142 | NCBI |  | KF260364 | fludb |  | FJ190135 | NCBI |
| DQ064555 | NCBI |  | AF523432 | NCBI |  | DQ681223 | NCBI |  | HQ425334 | NCBI |
| EF154846 | NCBI |  | CY023966 | NCBI |  | GQ373059 | NCBI |  | EF154966 | NCBI |
| EF154879 | fludb |  | CY023134 | NCBI |  | DQ226156 | NCBI |  | EF154964 | NCBI |
| CY024453 | fludb |  | CY024054 | NCBI |  | CY023767 | NCBI |  | CY023352 | NCBI |
| CY024373 | NCBI |  | CY024718 | NCBI |  | DQ997480 | NCBI |  | CY023424 | NCBI |
| CY024549 | NCBI |  | GQ477280 | NCBI |  | FJ793283 | NCBI |  | CY024392 | NCBI |
| EF154852 | NCBI |  | CY024030 | NCBI |  | KF835983 | NCBI |  | EF154971 | NCBI |
| EF154880 | NCBI |  | EF612748 | NCBI |  | DQ226157 | NCBI |  | EF154969 | NCBI |
| DQ485221 | NCBI |  | EU086280 | NCBI |  | EU532064 | NCBI |  | EF154972 | NCBI |
| CY024525 | NCBI |  | CY023694 | NCBI |  | CY024031 | NCBI |  | EF154970 | NCBI |
| DQ064552 | NCBI |  | HM590768 | NCBI |  | DQ997502 | NCBI |  | CY023480 | NCBI |
| CY024261 | fludb |  | EU086279 | NCBI |  | KF142480 | NCBI |  | CY023368 | NCBI |
| AF523463 | NCBI |  | EU086297 | NCBI |  | FJ793291 | fludb |  | CY024344 | NCBI |
| EF154870 | NCBI |  | EU086330 | NCBI |  | DQ681218 | NCBI |  | CY024328 | NCBI |
| CY024149 | NCBI |  | CY023726 | NCBI |  | EU365370 | NCBI |  | CY023392 | NCBI |
| DQ226174 | NCBI |  | EU086296 | NCBI |  | DQ997464 | NCBI |  | CY024416 | NCBI |
| KF260823 | NCBI |  | EU086331 | NCBI |  | DQ064502 | NCBI |  | EF154965 | NCBI |
| EU086301 | NCBI |  | CY023702 | NCBI |  | DQ997494 | NCBI |  | EU216081 | NCBI |
| AF222625 | fludb |  | CY023718 | NCBI |  | AY253752 | NCBI |  | EU216079 | fludb |
| JQ901629 | NCBI |  | HM590760 | fludb |  | DQ681206 | NCBI |  | GU471889 | NCBI |
| JQ901631 | NCBI |  | EU086315 | NCBI |  | DQ064513 | NCBI |  | CY023464 | NCBI |
| EF154845 | NCBI |  | EU086260 | NCBI |  | FJ581430 | NCBI |  | KF259069 | NCBI |
| EF154875 | NCBI |  | EU086232 | NCBI |  | EU935066 | NCBI |  | KF259061 | NCBI |
| EF154836 | NCBI |  | GU121380 | NCBI |  | DQ997444 | NCBI |  | EU216088 | NCBI |
| EF154844 | NCBI |  | GU474572 | NCBI |  | CY023191 | NCBI |  | KF259066 | NCBI |
| EF154851 | NCBI |  | FJ793282 | fludb |  | FJ793371 | NCBI |  | EU216082 | NCBI |
| CY023357 | NCBI |  | JX312548 | NCBI |  | DQ226158 | NCBI |  | CY023560 | NCBI |
| EF154855 | NCBI |  | KC417066 | NCBI |  | KC779057 | NCBI |  | CY023592 | NCBI |
| GU053185 | NCBI |  | DQ997417 | NCBI |  | EU516311 | NCBI |  | CY023576 | NCBI |
| CY024493 | NCBI |  | KC821070 | NCBI |  | DQ064496 | NCBI |  | EU216086 | NCBI |
| CY023845 | NCBI |  | CY023222 | NCBI |  | DQ997487 | NCBI |  | CY023608 | NCBI |
| EF154900 | NCBI |  | KF260645 | fludb |  | DQ997455 | NCBI |  | KF259074 | NCBI |
| DQ064557 | NCBI |  | KC821067 | NCBI |  | DQ681210 | NCBI |  | EU926626 | NCBI |
| EF154863 | NCBI |  | JN869536 | NCBI |  | DQ997432 | NCBI |  | KF259065 | NCBI |
| DQ226172 | NCBI |  | DQ997446 | NCBI |  | AY664761 | NCBI |  | EU216083 | NCBI |
| CY024736 | NCBI |  | CY023238 | NCBI |  | CY023751 | NCBI |  | CY023552 | NCBI |
| CY023709 | NCBI |  | CY023838 | NCBI |  | AY664765 | NCBI |  | EU216085 | NCBI |
| CY023981 | NCBI |  | GQ373036 | NCBI |  | FJ384750 | NCBI |  | KF259067 | NCBI |
| EF154884 | NCBI |  | KC821068 | NCBI |  | JQ904461 | NCBI |  | EU216084 | NCBI |
| CY024253 | NCBI |  | KC821069 | NCBI |  | DQ997191 | NCBI |  | CY024512 | fludb |
| CY024181 | NCBI |  | GQ373039 | NCBI |  | KC779058 | NCBI |  | EU216080 | NCBI |
| AF523465 | NCBI |  | KC821066 | NCBI |  | EF612747 | NCBI |  | CY023584 | NCBI |
| EF154899 | fludb |  | KF260703 | NCBI |  | EU086294 | NCBI |  | KF259064 | NCBI |
| EF154839 | NCBI |  | JN653587 | NCBI |  | EU086293 | NCBI |  | CY023600 | NCBI |
| KF188313 | NCBI |  | KC821071 | NCBI |  | CY023823 | fludb |  | EU157934 | NCBI |
| CY023621 | NCBI |  | KC821072 | NCBI |  | HM590761 | fludb |  | KF259062 | NCBI |
| DQ226173 | NCBI |  | KC821073 | NCBI |  | JF795045 | NCBI |  | CY023544 | NCBI |
| AF508654 | NCBI |  | GU474596 | NCBI |  | GQ477288 | NCBI |  | CY023472 | NCBI |
| CY024157 | NCBI |  | AY253751 | NCBI |  | CY023727 | NCBI |  | KF259079 | NCBI |
| JQ901636 | NCBI |  | KF178668 | fludb |  | EU086257 | NCBI |  | KF259070 | NCBI |
| CY023533 | fludb |  | KF260704 | NCBI |  | EU086278 | NCBI |  | KF188361 | NCBI |
| AF156432 | NCBI |  | KF746841 | NCBI |  | KC821103 | NCBI |  | KF259089 | NCBI |
| EF154903 | NCBI |  | GQ202046 | NCBI |  | GU121381 | NCBI |  | JF715046 | NCBI |
| EF154847 | NCBI |  | KF260642 | NCBI |  | GU474597 | NCBI |  | KF259085 | NCBI |
| CY024077 | NCBI |  | KC821074 | NCBI |  | CY023815 | NCBI |  | KF259095 | NCBI |
| EF154883 | NCBI |  | KF260617 | fludb |  | EU086329 | NCBI |  | JX122437 | NCBI |
| EF154840 | NCBI |  | KF178684 | NCBI |  | KC767258 | NCBI |  | KF259082 | NCBI |
| CY023421 | fludb |  | KC821082 | NCBI |  | GQ373054 | NCBI |  | KF259117 | NCBI |
| EF154857 | NCBI |  | KF715242 | NCBI |  | EU935067 | NCBI |  | KF259094 | NCBI |
| EF154834 | NCBI |  | KC821083 | NCBI |  | GQ477286 | NCBI |  | KF259091 | NCBI |
| CY023861 | NCBI |  | DQ681224 | NCBI |  | CY023695 | NCBI |  | CY023528 | NCBI |
| CY023853 | NCBI |  | CY023814 | NCBI |  | GQ477287 | fludb |  | KF259087 | NCBI |
| EF154882 | NCBI |  | KC821078 | NCBI |  | HM590769 | fludb |  | KF259080 | NCBI |
| EF154904 | NCBI |  | JF795092 | NCBI |  | GQ373053 | NCBI |  | KF259124 | NCBI |
| CY024501 | fludb |  | KF746793 | NCBI |  | EU935065 | NCBI |  | KF259106 | NCBI |
| EF154866 | fludb |  | KF260634 | NCBI |  | EU086313 | NCBI |  | KF259063 | NCBI |
| CY024461 | NCBI |  | JN653585 | NCBI |  | GU474573 | NCBI |  | JF715015 | NCBI |
| JQ901634 | NCBI |  | FJ013034 | NCBI |  | EU532059 | NCBI |  | KF259104 | NCBI |
| CY023805 | NCBI |  | KF260611 | NCBI |  | GU474581 | NCBI |  | KF259073 | NCBI |
| AF523464 | NCBI |  | GU474580 | NCBI |  | EU086328 | NCBI |  | KC820992 | NCBI |
| KF260867 | NCBI |  | KC821079 | NCBI |  | GU474605 | NCBI |  | KF259107 | NCBI |
| AY043030 | NCBI |  | GU474588 | NCBI |  | KF746842 | NCBI |  | KF259096 | fludb |
| EF154886 | NCBI |  | KF260643 | NCBI |  | CY023839 | NCBI |  | KF259093 | NCBI |
| EF154877 | NCBI |  | CY024550 | NCBI |  | KF746794 | NCBI |  | KF259090 | NCBI |
| JQ901628 | NCBI |  | KF260697 | NCBI |  | EU086231 | NCBI |  | KF259075 | NCBI |
| EF154869 | NCBI |  | KF367737 | NCBI |  | CY023719 | NCBI |  | JF715052 | NCBI |
| CY024125 | NCBI |  | CY023214 | NCBI |  | GU474565 | NCBI |  | JN986883 | NCBI |
| CY024205 | fludb |  | CY087169 | NCBI |  | CY023703 | NCBI |  | KF259092 | NCBI |
| CY024269 | NCBI |  | KC821075 | NCBI |  | EU086259 | NCBI |  | KF259126 | NCBI |
| AJ404631 | NCBI |  | KC821076 | NCBI |  | KF746858 | NCBI |  | KF259121 | NCBI |
| EF154856 | fludb |  | DQ997192 | NCBI |  | GU474549 | NCBI |  | KF259105 | NCBI |
| EF154867 | NCBI |  | JN653583 | NCBI |  | EU532062 | NCBI |  | KF259122 | NCBI |
| EF154868 | NCBI |  | KF260647 | NCBI |  | GU474589 | NCBI |  | KF259084 | NCBI |
| JQ901626 | NCBI |  | KF260635 | NCBI |  | EU086312 | NCBI |  | KF259100 | NCBI |
| CY024557 | fludb |  | KC821080 | NCBI |  | KF715235 | NCBI |  | JF715049 | NCBI |
| CY023797 | fludb |  | KF178692 | NCBI |  | FJ492966 | NCBI |  | JF715025 | NCBI |
| EF154858 | NCBI |  | KC779060 | NCBI |  | GQ373064 | NCBI |  | KF259113 | NCBI |
| EF154906 | NCBI |  | GQ202060 | NCBI |  | GQ202063 | NCBI |  | JF715016 | NCBI |
| CY024221 | fludb |  | KC821084 | NCBI |  | JN653604 | NCBI |  | KF259138 | NCBI |
| CY024085 | NCBI |  | DQ064540 | NCBI |  | JN653590 | NCBI |  | JX448762 | NCBI |
| CY023885 | NCBI |  | JF795068 | NCBI |  | GQ202049 | NCBI |  | JN804527 | NCBI |
| CY023869 | fludb |  | KC417064 | NCBI |  | KF715227 | NCBI |  | JN804201 | fludb |
| CY024213 | fludb |  | JN653577 | NCBI |  | KC768055 | NCBI |  | JN986892 | NCBI |
| EF154849 | NCBI |  | KC779059 | NCBI |  | JN653594 | NCBI |  | JF715027 | NCBI |
| CY023877 | NCBI |  | GQ373040 | NCBI |  | KC821105 | NCBI |  | JN804207 | NCBI |
| EF154859 | NCBI |  | CY024102 | NCBI |  | GQ373056 | NCBI |  | JN804206 | NCBI |
| EF154876 | NCBI |  | JF795052 | NCBI |  | JN653596 | NCBI |  | JN986893 | NCBI |
| EF154841 | fludb |  | KF260640 | fludb |  | GQ202043 | NCBI |  | JN804528 | NCBI |
| EF154850 | NCBI |  | CY146658 | NCBI |  | JN653601 | NCBI |  | JF715044 | NCBI |
| AF156435 | NCBI |  | KF260648 | NCBI |  | JN653598 | NCBI |  | JN804205 | NCBI |
| DQ064551 | NCBI |  | KF260688 | NCBI |  | KC821106 | NCBI |  | KF259142 | fludb |
| EF154901 | NCBI |  | KC821077 | NCBI |  | JF906205 | NCBI |  | JN804204 | NCBI |
| JQ901627 | NCBI |  | CY087185 | NCBI |  | KF746818 | NCBI |  | JN804509 | NCBI |
| EF154874 | NCBI |  | KF746849 | fludb |  | HM751153 | NCBI |  | JN804202 | NCBI |
| CY024045 | NCBI |  | JQ356889 | NCBI |  | KC821107 | NCBI |  | JN804457 | NCBI |
| CY024309 | NCBI |  | FJ581435 | NCBI |  | HM998921 | NCBI |  | JN804203 | NCBI |
| AF222622 | NCBI |  | KC821081 | NCBI |  | KF746747 | NCBI |  | JN804214 | NCBI |
| CY023277 | NCBI |  | KF260637 | NCBI |  | JQ356881 | NCBI |  | JN804215 | NCBI |
| EF154905 | NCBI |  | AF523442 | NCBI |  | FJ499470 | NCBI |  | JN804512 | NCBI |
| CY023957 | NCBI |  | KF260633 | NCBI |  | KF746786 | NCBI |  | JF715051 | NCBI |
| EF154885 | NCBI |  | GQ202052 | fludb |  | KF714774 | NCBI |  | JF715047 | NCBI |
| CY024541 | NCBI |  | DQ997495 | NCBI |  | JN653593 | NCBI |  | KF259141 | NCBI |
| CY024397 | fludb |  | CY023990 | NCBI |  | KF746768 | NCBI |  | JN804526 | NCBI |
| CY023997 | NCBI |  | KC821085 | NCBI |  | KF835985 | NCBI |  | JF715042 | NCBI |
| AF222623 | NCBI |  | KC821086 | NCBI |  | FJ534545 | NCBI |  | JN804216 | NCBI |
| CY024229 | NCBI |  | KC821092 | NCBI |  | KC821104 | NCBI |  | JN804556 | NCBI |
| AF222626 | fludb |  | KC821097 | NCBI |  | GQ202055 | NCBI |  | JN869546 | NCBI |
| DQ064569 | NCBI |  | KC821098 | NCBI |  | KC768056 | NCBI |  | KF259134 | NCBI |
| EF154873 | NCBI |  | JF795076 | fludb |  | KC821108 | NCBI |  | JX448761 | NCBI |
| EF154898 | NCBI |  | KC821099 | NCBI |  | HM751185 | NCBI |  | KF259139 | NCBI |
| JQ901632 | NCBI |  | KC821087 | NCBI |  | KC821109 | NCBI |  | JX122428 | NCBI |
| CY024189 | NCBI |  | CY023766 | NCBI |  | KC821110 | NCBI |  | JN804557 | NCBI |
| AF222624 | fludb |  | KC821090 | NCBI |  | JN653595 | NCBI |  | JF714998 | NCBI |
| EU081871 | NCBI |  | CY023230 | NCBI |  | JN653592 | NCBI |  | KF259148 | NCBI |
| AF523466 | NCBI |  | KC821089 | NCBI |  | HM751193 | NCBI |  | KF259145 | NCBI |
| EF154854 | NCBI |  | JN653575 | NCBI |  | KC821111 | NCBI |  | KC820995 | NCBI |
| AF258835 | NCBI |  | JQ228395 | NCBI |  | KC821117 | NCBI |  | JN804555 | NCBI |
| EF154888 | NCBI |  | DQ064529 | NCBI |  | KC821118 | NCBI |  | JN986895 | NCBI |
| JQ901635 | NCBI |  | JQ356887 | NCBI |  | KC821126 | NCBI |  | JQ770132 | NCBI |
| CY024533 | NCBI |  | KC821095 | NCBI |  | KC821122 | NCBI |  | JF715028 | NCBI |
| DQ064553 | NCBI |  | KF500983 | NCBI |  | CY087178 | NCBI |  | KF259127 | NCBI |
| EF154864 | NCBI |  | CY023822 | NCBI |  | KF367738 | NCBI |  | JN986896 | fludb |
| CY023317 | NCBI |  | KF746817 | NCBI |  | JF795037 | NCBI |  | KF259140 | fludb |
| AF156431 | NCBI |  | DQ997503 | NCBI |  | KC821116 | NCBI |  | KF259149 | NCBI |
| EF154881 | NCBI |  | JF906204 | NCBI |  | JN653602 | NCBI |  | KF259157 | NCBI |
| CY024421 | fludb |  | KF260630 | NCBI |  | KC821127 | NCBI |  | JN986894 | NCBI |
| CY023293 | NCBI |  | CY024166 | fludb |  | CY087170 | NCBI |  | JX122433 | NCBI |
| EF154865 | NCBI |  | JF795142 | NCBI |  | JN653599 | NCBI |  | JF715021 | NCBI |
| CY023285 | NCBI |  | KF260706 | NCBI |  | JN653605 | NCBI |  | JF715031 | NCBI |
| CY024293 | NCBI |  | KC821088 | NCBI |  | JF795101 | NCBI |  | KF259143 | NCBI |
| EF154853 | NCBI |  | JF795100 | NCBI |  | CY087186 | NCBI |  | JF715020 | NCBI |
| EF154878 | NCBI |  | KC821091 | NCBI |  | JN653591 | NCBI |  | JX122426 | NCBI |
| KC821033 | NCBI |  | KC417065 | NCBI |  | JF795077 | NCBI |  | JN986891 | NCBI |
| KC821034 | NCBI |  | DQ064523 | NCBI |  | KC821124 | NCBI |  | JF714999 | NCBI |
| KC832035 | NCBI |  | KC821091 | NCBI |  | JF795053 | NCBI |  | JQ770137 | NCBI |
| KC821036 | NCBI |  | GQ477282 | NCBI |  | GQ373055 | NCBI |  | JF715019 | NCBI |
| KF260879 | NCBI |  | KC768058 | NCBI |  | KC821123 | NCBI |  | JX122438 | NCBI |
| CY087176 | NCBI |  | KF260639 | NCBI |  | JF795143 | NCBI |  | JF715048 | NCBI |
| CY146657 | NCBI |  | GQ477279 | NCBI |  | KF746850 | NCBI |  | JX122432 | fludb |
| KF260951 | NCBI |  | KF260622 | NCBI |  | KC821121 | NCBI |  | JF715024 | NCBI |
| JF795059 | NCBI |  | CY087177 | NCBI |  | KC821120 | NCBI |  | JF715050 | NCBI |
| KF260881 | NCBI |  | KC821093 | NCBI |  | GQ373057 | NCBI |  | JQ770130 | NCBI |
| KC821037 | NCBI |  | KC821094 | NCBI |  | JN653603 | NCBI |  | KF259137 | NCBI |
| KC821038 | NCBI |  | JN653579 | NCBI |  | HM998913 | NCBI |  | JQ770141 | NCBI |
| KC821039 | NCBI |  | KF746767 | NCBI |  | KC821119 | NCBI |  | KF259158 | NCBI |
| KF260941 | NCBI |  | KC464596 | NCBI |  | KC821125 | NCBI |  | JF715023 | NCBI |
| CY087184 | NCBI |  | KF260700 | fludb |  | JF795061 | NCBI |  | KF259153 | NCBI |
| JQ356886 | NCBI |  | KC821096 | NCBI |  | JF795093 | NCBI |  | KF259144 | NCBI |
| KC821040 | NCBI |  | JN653589 | NCBI |  | KC821129 | NCBI |  | KF259152 | NCBI |
| KC821041 | NCBI |  | GU474564 | NCBI |  | KF260456 | NCBI |  | KF259151 | NCBI |
| KF260877 | NCBI |  | CY024006 | NCBI |  | KC821136 | NCBI |  | JF715022 | NCBI |
| FJ581429 | NCBI |  | FJ793290 | NCBI |  | JN653597 | NCBI |  | KF259147 | NCBI |
| CY024029 | NCBI |  | KF260641 | NCBI |  | CY146659 | NCBI |  | JQ770135 | NCBI |
| JF795035 | NCBI |  | KF715234 | NCBI |  | KF260368 | NCBI |  | JF715038 | NCBI |
| KC821042 | NCBI |  | KC821100 | NCBI |  | KF260458 | NCBI |  | KF259154 | NCBI |
| KF500984 | NCBI |  | KF059334 | NCBI |  | KF835981 | NCBI |  | JQ770140 | NCBI |
| JN653562 | NCBI |  | KF059335 | NCBI |  | JF795085 | NCBI |  | JF715034 | NCBI |
| KC821043 | NCBI |  | CY023254 | NCBI |  | KC821133 | NCBI |  | JQ770131 | NCBI |
| KC821044 | NCBI |  | KF746857 | NCBI |  | KF260467 | NCBI |  | KF259150 | NCBI |
| JN869528 | NCBI |  | KF059336 | NCBI |  | JN653600 | NCBI |  | JX122436 | NCBI |
| KC821045 | NCBI |  | KF260691 | NCBI |  | KC821134 | NCBI |  | KF259155 | NCBI |
| KC821046 | NCBI |  | JN653581 | NCBI |  | KC821113 | NCBI |  | JQ770133 | NCBI |
| KF260868 | NCBI |  | CY023246 | NCBI |  | KF260452 | NCBI |  | JF715035 | NCBI |
| JF519815 | NCBI |  | JN653586 | NCBI |  | KF714782 | NCBI |  | JN869538 | NCBI |
| KC821047 | NCBI |  | KF059337 | NCBI |  | KF260457 | NCBI |  | JQ770136 | NCBI |
| KC768060 | NCBI |  | KF059338 | NCBI |  | JN869530 | NCBI |  | KF259146 | NCBI |
| KC821048 | NCBI |  | KF142479 | fludb |  | KC821112 | NCBI |  | JQ770134 | NCBI |
| JN653572 | NCBI |  | KF059339 | NCBI |  | KC821114 | NCBI |  | KF259156 | NCBI |
| JF906203 | NCBI |  | CY024174 | fludb |  | KF260454 | fludb |  | DQ981538 | NCBI |
| KC821049 | NCBI |  | KF059340 | NCBI |  | JQ356883 | NCBI |  | KF188276 | fludb |
| JX312549 | NCBI |  | KF260627 | fludb |  | KF260461 | NCBI |  | CY075030 | NCBI |
| JN869514 | NCBI |  | JN653582 | NCBI |  | JX312547 | NCBI |  | DQ981546 | NCBI |
| KF260893 | NCBI |  | JN653580 | NCBI |  | KF260367 | NCBI |  | KF188272 | fludb |
| GQ373021 | NCBI |  | KF059341 | NCBI |  | JN869523 | NCBI |  | CY075038 | NCBI |
| KF260888 | NCBI |  | KC767257 | NCBI |  | KF715243 | NCBI |  | CY075046 | NCBI |
| KC821050 | NCBI |  | DQ997434 | NCBI |  | KF260455 | NCBI |  | AY623810 | NCBI |
| JQ228396 | NCBI |  | JN869522 | NCBI |  | JQ228397 | NCBI |  | CY075054 | NCBI |
| KC821051 | NCBI |  | KF746787 | NCBI |  | JN222380 | NCBI |  | CY006021 | NCBI |
| KC821052 | NCBI |  | KF260612 | NCBI |  | KF260393 | NCBI |  | DQ981594 | NCBI |
| KF260899 | NCBI |  | KF059342 | NCBI |  | KF260396 | NCBI |  | DQ981562 | NCBI |
| KF260870 | NCBI |  | KF059343 | NCBI |  | KF260385 | fludb |  | CY014613 | NCBI |
| KC821053 | NCBI |  | JN869529 | NCBI |  | KF260409 | NCBI |  | AY206677 | NCBI |
| KC821054 | NCBI |  | DQ681214 | NCBI |  | JN869545 | NCBI |  | DQ981586 | NCBI |
| KC821055 | NCBI |  | GU474604 | NCBI |  | KF260391 | NCBI |  | DQ981570 | NCBI |
| KF260934 | NCBI |  | KF260644 | fludb |  | JN869537 | NCBI |  | DQ981554 | NCBI |
| JN653567 | NCBI |  | GQ477283 | NCBI |  | KF260398 | NCBI |  | GQ373070 | NCBI |
| KF260947 | NCBI |  | KF260689 | NCBI |  | KF260388 | NCBI |  | FJ190118 | NCBI |
| KC821056 | NCBI |  | KM609800 | NCBI |  | KC821135 | NCBI |  | KC820994 | NCBI |
| KF260882 | NCBI |  | KM609801 | NCBI |  | KF260405 | NCBI |  | FJ793444 | NCBI |
| KC821057 | NCBI |  | KF260621 | NCBI |  | KF260389 | NCBI |  | FJ190134 | NCBI |
| KF260946 | NCBI |  | KF746746 | fludb |  | KF260397 | NCBI |  | FJ190141 | NCBI |
| KC821058 | NCBI |  | CY023262 | NCBI |  | KF260392 | NCBI |  | FJ190151 | NCBI |
| KF714772 | NCBI |  | KM609802 | NCBI |  | KF260408 | NCBI |  | FJ190114 | NCBI |
| KF260861 | fludb |  | EU365369 | NCBI |  | KF260399 | NCBI |  | FJ190139 | NCBI |
| CY087168 | NCBI |  | KF260646 | NCBI |  | KF260390 | fludb |  | KF715236 | NCBI |
| KC417069 | NCBI |  | KF260650 | NCBI |  | KF260384 | NCBI |  | CY006025 | NCBI |
| KC821059 | NCBI |  | GQ373038 | NCBI |  | KF260402 | NCBI |  | KF746843 | NCBI |
| KF260889 | fludb |  | KM609803 | NCBI |  | KF260400 | fludb |  | AF461519 | NCBI |
| KF260943 | fludb |  | KF260707 | fludb |  | KF260381 | NCBI |  | KF746867 | NCBI |
| KF260935 | NCBI |  | KF260638 | NCBI |  | KF260377 | NCBI |  | EU753346 | NCBI |
| KC821060 | NCBI |  | JQ356888 | NCBI |  | KF260407 | NCBI |  | AF508567 | NCBI |
| JN869535 | NCBI |  | KM609804 | NCBI |  | KF746801 | NCBI |  | AY043017 | NCBI |
| KJ128369 | NCBI |  | KM609805 | NCBI |  | KF260380 | NCBI |  | FJ793420 | NCBI |
| JF519814 | NCBI |  | KF260699 | NCBI |  | KF260406 | NCBI |  | CY006023 | NCBI |
| JQ356884 | NCBI |  | KJ128368 | NCBI |  | KF260401 | fludb |  | KF746859 | NCBI |
| KC821061 | NCBI |  | KF609806 | NCBI |  | KF260387 | NCBI |  | FJ793300 | NCBI |
| JN869521 | NCBI |  | KF609807 | NCBI |  | KF260412 | NCBI |  | KC817007 | NCBI |
| KC821062 | NCBI |  | KF609808 | NCBI |  | KF260378 | fludb |  | FJ190140 | NCBI |
| JN653566 | NCBI |  | KF260632 | NCBI |  | KF260403 | NCBI |  | AY043018 | NCBI |
| KC821063 | NCBI |  | KF260628 | NCBI |  | KF260386 | NCBI |  | AF461518 | NCBI |
| KF059344 | NCBI |  | JF795060 | NCBI |  | KF260410 | NCBI |  | GQ477292 | NCBI |
| KF059345 | NCBI |  | CY024198 | fludb |  | KF260404 | NCBI |  | DQ064368 | NCBI |
| KF715241 | NCBI |  | KM609809 | NCBI |  | KF260394 | fludb |  | FJ793332 | NCBI |
| KF260894 | NCBI |  | KF260629 | fludb |  | KF260395 | NCBI |  | KF746795 | NCBI |
| KF260860 | NCBI |  | GQ202040 | NCBI |  | KF260374 | NCBI |  | FJ190119 | NCBI |
| KF260933 | NCBI |  | KM609810 | NCBI |  | KC821115 | NCBI |  | FJ190144 | NCBI |
| KF059346 | NCBI |  | JF795084 | NCBI |  | KC821128 | NCBI |  | KF746777 | NCBI |
| KF260876 | NCBI |  | JN653584 | NCBI |  | KF260372 | NCBI |  | DQ485224 | NCBI |
| KF059347 | NCBI |  | KM609811 | NCBI |  | KC821131 | NCBI |  | FJ190133 | NCBI |
| KC464595 | NCBI |  | KF260652 | NCBI |  | KC821132 | NCBI |  | FJ807713 | NCBI |
| KF059348 | NCBI |  | GQ373042 | NCBI |  | KC821130 | NCBI |  | CY063662 | NCBI |
| JN653559 | NCBI |  | KF260655 | NCBI |  | KF260382 | NCBI |  | FJ499463 | NCBI |
| KF059349 | NCBI |  | FJ534542 | NCBI |  | KF178696 | NCBI |  | JF795062 | NCBI |
| JN653565 | NCBI |  | KF715226 | NCBI |  | KC821137 | NCBI |  | KJ419941 | NCBI |
| KF260886 | NCBI |  | KM609812 | NCBI |  | KF260366 | NCBI |  | GQ202036 | NCBI |
| KF059350 | NCBI |  | HM998920 | NCBI |  | KF178688 | NCBI |  | FJ807718 | NCBI |
| JF519817 | NCBI |  | KF260626 | NCBI |  | KF059324 | NCBI |  | FJ534548 | fludb |
| KC779061 | NCBI |  | KM609813 | NCBI |  | KF059325 | NCBI |  | FJ807715 | fludb |
| KF260862 | fludb |  | KF260696 | NCBI |  | KF059326 | NCBI |  | FJ807716 | NCBI |
| KF260880 | NCBI |  | KF260656 | NCBI |  | KF059327 | NCBI |  | JF795078 | NCBI |
| KF059351 | NCBI |  | JN653578 | NCBI |  | KC417061 | NCBI |  | FJ534550 | fludb |
| KF059352 | NCBI |  | KM609814 | NCBI |  | KF059328 | NCBI |  | FJ807714 | NCBI |
| KC768061 | NCBI |  | KF260690 | NCBI |  | JF795069 | NCBI |  | FJ534538 | NCBI |
| KF260898 | NCBI |  | CY023750 | NCBI |  | KF260383 | fludb |  | KC820993 | NCBI |
| KF059353 | NCBI |  | GQ373047 | NCBI |  | KF059329 | NCBI |  | FJ534546 | NCBI |
| KM609840 | NCBI |  | HM751184 | NCBI |  | KF059330 | NCBI |  | KC820996 | NCBI |
| KF260878 | NCBI |  | KM609815 | NCBI |  | KF059331 | NCBI |  | GQ202056 | NCBI |
| KM609841 | NCBI |  | EU516310 | NCBI |  | KF260411 | NCBI |  | KJ419954 | NCBI |
| KF260884 | fludb |  | KF260692 | NCBI |  | KF260447 | NCBI |  | FJ807711 | NCBI |
| KF260900 | NCBI |  | KF260687 | NCBI |  | KF260443 | NCBI |  | KC820997 | NCBI |
| KC417067 | NCBI |  | KM609816 | NCBI |  | KF059332 | NCBI |  | CY024144 | NCBI |
| JN653569 | NCBI |  | KM609817 | NCBI |  | KF059333 | NCBI |  | CY024656 | NCBI |
| KF260948 | NCBI |  | HM998912 | NCBI |  | KF260444 | NCBI |  | EF154928 | NCBI |
| KF260865 | NCBI |  | JN653574 | NCBI |  | JQ356882 | NCBI |  | JQ710463 | NCBI |
| KF260949 | fludb |  | DQ681202 | NCBI |  | KF260373 | NCBI |  | CY024600 | NCBI |
| KF260872 | NCBI |  | KM609818 | NCBI |  | KC464597 | NCBI |  | CY023104 | NCBI |
| KF260944 | NCBI |  | KF260653 | NCBI |  | KM609760 | NCBI |  | FJ190127 | NCBI |
| KM609842 | NCBI |  | KF260651 | NCBI |  | KF260462 | NCBI |  | CY023296 | NCBI |
| KF260942 | NCBI |  | KC768059 | NCBI |  | KC417063 | NCBI |  | CY023312 | NCBI |
| JN653563 | NCBI |  | KF260702 | NCBI |  | KM609761 | NCBI |  | DQ064372 | NCBI |
| JN653560 | NCBI |  | JN222386 | NCBI |  | KM609762 | NCBI |  | FJ231868 | NCBI |
| KF260936 | NCBI |  | KF260625 | NCBI |  | KM609763 | NCBI |  | CY023152 | NCBI |
| KM609843 | NCBI |  | KM609819 | NCBI |  | KF178664 | fludb |  | CY024176 | NCBI |
| JN653568 | NCBI |  | KM609820 | NCBI |  | KM609764 | NCBI |  | CY023328 | NCBI |
| JN653573 | NCBI |  | KF260624 | NCBI |  | KM609765 | NCBI |  | CY023232 | NCBI |
| KF260883 | NCBI |  | KF260708 | NCBI |  | KM609766 | NCBI |  | CY024056 | NCBI |
| KM609844 | NCBI |  | KM609821 | NCBI |  | KM609767 | NCBI |  | CY023184 | NCBI |
| KF260869 | NCBI |  | KF260631 | NCBI |  | KM609768 | NCBI |  | EF154916 | NCBI |
| JF795051 | NCBI |  | EU882863 | NCBI |  | KF260464 | fludb |  | DQ226111 | NCBI |
| JN653564 | NCBI |  | JF795036 | NCBI |  | KM609769 | NCBI |  | CY024680 | NCBI |
| KM609845 | NCBI |  | KM609822 | NCBI |  | KC417062 | NCBI |  | CY023168 | NCBI |
| KM609846 | NCBI |  | KM609823 | NCBI |  | KM609770 | NCBI |  | DQ064359 | NCBI |
| KF260895 | NCBI |  | JF795044 | NCBI |  | KF260459 | NCBI |  | CY023336 | NCBI |
| KF260940 | NCBI |  | KF260616 | NCBI |  | KM609771 | NCBI |  | CY023304 | NCBI |
| KM609847 | NCBI |  | KF260705 | NCBI |  | KM609772 | NCBI |  | CY023912 | NCBI |
| KM609848 | NCBI |  | GU474548 | NCBI |  | KC951121 | NCBI |  | AY664675 | NCBI |
| KF260871 | NCBI |  | JN869544 | NCBI |  | KM609773 | NCBI |  | EF154929 | fludb |
| KM609849 | NCBI |  | FJ499467 | NCBI |  | KM609774 | NCBI |  | CY024728 | NCBI |
| JF519809 | NCBI |  | KC951120 | NCBI |  | KM609775 | NCBI |  | CY024648 | NCBI |
| KF260874 | NCBI |  | KM609824 | NCBI |  | KF260460 | fludb |  | CY023136 | NCBI |
| KM609850 | NCBI |  | KF714781 | NCBI |  | KM609776 | NCBI |  | DQ064356 | NCBI |
| JF795141 | NCBI |  | JN653588 | NCBI |  | KM609777 | NCBI |  | CY023320 | NCBI |
| KM609851 | NCBI |  | KM609825 | NCBI |  | KF260463 | fludb |  | CY024704 | NCBI |
| KC768062 | NCBI |  | KF260636 | NCBI |  | KM609778 | NCBI |  | CY023928 | NCBI |
| KF260854 | NCBI |  | KM609826 | NCBI |  | KM609779 | NCBI |  | CY024240 | NCBI |
| HM998919 | NCBI |  | KM609827 | NCBI |  | KJ128367 | NCBI |  | CY023120 | NCBI |
| JN653561 | NCBI |  | KM609828 | NCBI |  | KM609780 | NCBI |  | CY023968 | NCBI |
| KM609852 | NCBI |  | KF260649 | NCBI |  | KF260453 | NCBI |  | CY023944 | NCBI |
| KM609853 | NCBI |  | FJ384749 | NCBI |  | KF260446 | NCBI |  | CY024712 | NCBI |
| JN869543 | NCBI |  | CY024022 | NCBI |  | KM609781 | NCBI |  | CY023952 | NCBI |
| KF260885 | NCBI |  | KM609829 | NCBI |  | KM609782 | NCBI |  | CY023208 | NCBI |
| KF260887 | NCBI |  | KF260701 | fludb |  | KM609783 | NCBI |  | CY023448 | NCBI |
| KM609854 | NCBI |  | HM751192 | NCBI |  | KF500982 | NCBI |  | CY024720 | NCBI |
| KF260931 | NCBI |  | KF260698 | NCBI |  | KM609784 | NCBI |  | EF154934 | NCBI |
| KF260891 | NCBI |  | DQ681219 | NCBI |  | KM609785 | NCBI |  | CY023936 | NCBI |
| JF795091 | NCBI |  | JN653576 | NCBI |  | KM609786 | NCBI |  | EF154947 | NCBI |
| KF260950 | NCBI |  | KM609830 | NCBI |  | KF178680 | NCBI |  | CY023216 | NCBI |
| KM609855 | NCBI |  | KF260610 | NCBI |  | KF260445 | NCBI |  | CY024384 | NCBI |
| KC951119 | NCBI |  | KM609831 | NCBI |  | KM609787 | NCBI |  | CY024688 | NCBI |
| HM751191 | NCBI |  | KF746800 | NCBI |  | KM609788 | NCBI |  | CY024096 | NCBI |
| KM609856 | NCBI |  | GQ373037 | NCBI |  | KM609789 | NCBI |  | CY023456 | NCBI |
| KM609857 | NCBI |  | KM609832 | NCBI |  | KF260448 | NCBI |  | CY023408 | NCBI |
| GQ373025 | NCBI |  | KM609833 | NCBI |  | KM609790 | NCBI |  | CY023144 | NCBI |
| KM609858 | NCBI |  | KF260618 | fludb |  | KM609791 | NCBI |  | CY023200 | NCBI |
| KM609859 | NCBI |  | KM609834 | NCBI |  | KM609792 | NCBI |  | EF154953 | NCBI |
| KF260873 | fludb |  | KF260654 | NCBI |  | KM609793 | NCBI |  | CY024480 | NCBI |
| KF260856 | NCBI |  | KM609835 | NCBI |  | KM609794 | NCBI |  | CY023176 | NCBI |
| KM609860 | NCBI |  | KM609836 | NCBI |  | KM609795 | NCBI |  | CY024488 | NCBI |
| KF714780 | NCBI |  | KM609837 | NCBI |  | KM609796 | NCBI |  | EF154950 | NCBI |
| KF260932 | NCBI |  | KM609838 | NCBI |  | KM609797 | NCBI |  | CY023160 | NCBI |
| JF795083 | NCBI |  | KM609839 | NCBI |  | KM609798 | NCBI |  | CY023128 | NCBI |
| KM609861 | NCBI |  |  |  |  | KM609799 | NCBI |  | CY024128 | NCBI |
| KM609862 | NCBI |  |  |  |  |  |  |  | CY023112 | NCBI |
| KM609863 | NCBI |  |  |  |  |  |  |  | CY023920 | NCBI |
| KF260945 | fludb |  |  |  |  |  |  |  | CY023416 | NCBI |
| JF519818 | NCBI |  |  |  |  |  |  |  | CY023632 | fludb |
| KF260892 | NCBI |  |  |  |  |  |  |  | EF154954 | NCBI |
| KM609864 | NCBI |  |  |  |  |  |  |  | CY024040 | NCBI |
| KF260896 | NCBI |  |  |  |  |  |  |  | CY024104 | NCBI |
| KM609865 | NCBI |  |  |  |  |  |  |  | GU471803 | NCBI |
| GQ335487 | NCBI |  |  |  |  |  |  |  | CY023224 | NCBI |
| KF260897 | NCBI |  |  |  |  |  |  |  | GU471892 | NCBI |
| KM609866 | NCBI |  |  |  |  |  |  |  | CY023648 | NCBI |
| JF519810 | NCBI |  |  |  |  |  |  |  | CY023240 | NCBI |
| KC417068 | NCBI |  |  |  |  |  |  |  | CY023688 | NCBI |
| JQ356885 | NCBI |  |  |  |  |  |  |  | CY023264 | NCBI |
| KF260875 | NCBI |  |  |  |  |  |  |  | CY023640 | NCBI |
| KF260855 | NCBI |  |  |  |  |  |  |  | CY023616 | NCBI |
| CY024013 | NCBI |  |  |  |  |  |  |  | GU471897 | NCBI |
| KF260890 | NCBI |  |  |  |  |  |  |  | CY023248 | NCBI |
| KM609867 | NCBI |  |  |  |  |  |  |  | CY024024 | NCBI |
| KF260952 | NCBI |  |  |  |  |  |  |  | CY024168 | fludb |
| JF519819 | NCBI |  |  |  |  |  |  |  | GU471801 | NCBI |
| KM609868 | NCBI |  |  |  |  |  |  |  | CY023496 | NCBI |
| JF795067 | NCBI |  |  |  |  |  |  |  | CY024008 | NCBI |
| KC779062 | NCBI |  |  |  |  |  |  |  | CY024520 | fludb |
| JN653558 | NCBI |  |  |  |  |  |  |  | DQ226112 | NCBI |
| KM609869 | NCBI |  |  |  |  |  |  |  | CY023512 | NCBI |
| KF260866 | fludb |  |  |  |  |  |  |  | GU471885 | NCBI |
| KM609870 | NCBI |  |  |  |  |  |  |  | CY023256 | NCBI |
| KM609871 | NCBI |  |  |  |  |  |  |  | CY024696 | NCBI |
| KM609872 | NCBI |  |  |  |  |  |  |  | CY023520 | NCBI |
| KM609873 | NCBI |  |  |  |  |  |  |  | FJ190123 | NCBI |
| KM609874 | NCBI |  |  |  |  |  |  |  | CY023992 | NCBI |
| KM609875 | NCBI |  |  |  |  |  |  |  | CY024200 | NCBI |
| KM609876 | NCBI |  |  |  |  |  |  |  | DQ064357 | NCBI |
| KM609877 | NCBI |  |  |  |  |  |  |  | AY664670 | NCBI |
| KM609878 | NCBI |  |  |  |  |  |  |  | CY023488 | NCBI |
| KM609879 | NCBI |  |  |  |  |  |  |  | CY023680 | NCBI |
|  |  |  |  |  |  |  |  |  | JN986889 | NCBI |
|  |  |  |  |  |  |  |  |  | JX122430 | NCBI |
|  |  |  |  |  |  |  |  |  | JX122431 | NCBI |
|  |  |  |  |  |  |  |  |  | FJ434574 | NCBI |
|  |  |  |  |  |  |  |  |  | GU474598 | NCBI |
|  |  |  |  |  |  |  |  |  | GU471794 | NCBI |
|  |  |  |  |  |  |  |  |  | JN571286 | NCBI |
|  |  |  |  |  |  |  |  |  | CY023664 | NCBI |
|  |  |  |  |  |  |  |  |  | HM773440 | NCBI |
|  |  |  |  |  |  |  |  |  | JQ228390 | NCBI |
|  |  |  |  |  |  |  |  |  | FJ434587 | NCBI |
|  |  |  |  |  |  |  |  |  | FJ434569 | NCBI |
|  |  |  |  |  |  |  |  |  | FJ434585 | NCBI |
|  |  |  |  |  |  |  |  |  | JF715008 | NCBI |
|  |  |  |  |  |  |  |  |  | JX448747 | NCBI |
|  |  |  |  |  |  |  |  |  | JN222381 | NCBI |
|  |  |  |  |  |  |  |  |  | FJ434575 | NCBI |
|  |  |  |  |  |  |  |  |  | FJ434582 | NCBI |
|  |  |  |  |  |  |  |  |  | FJ434572 | NCBI |
|  |  |  |  |  |  |  |  |  | JN571285 | NCBI |
|  |  |  |  |  |  |  |  |  | JX122429 | NCBI |
|  |  |  |  |  |  |  |  |  | FJ434570 | NCBI |
|  |  |  |  |  |  |  |  |  | GU474606 | NCBI |
|  |  |  |  |  |  |  |  |  | FJ434573 | NCBI |
|  |  |  |  |  |  |  |  |  | CY023672 | NCBI |
|  |  |  |  |  |  |  |  |  | FJ434571 | NCBI |
|  |  |  |  |  |  |  |  |  | HM008887 | NCBI |
|  |  |  |  |  |  |  |  |  | FJ434580 | NCBI |
|  |  |  |  |  |  |  |  |  | GQ463225 | NCBI |
|  |  |  |  |  |  |  |  |  | KF259098 | NCBI |
|  |  |  |  |  |  |  |  |  | JN802578 | NCBI |
|  |  |  |  |  |  |  |  |  | GQ463215 | NCBI |
|  |  |  |  |  |  |  |  |  | HQ398348 | NCBI |
|  |  |  |  |  |  |  |  |  | KF259086 | NCBI |
|  |  |  |  |  |  |  |  |  | KF259088 | NCBI |
|  |  |  |  |  |  |  |  |  | JX273563 | NCBI |
|  |  |  |  |  |  |  |  |  | HQ398337 | NCBI |
|  |  |  |  |  |  |  |  |  | KF259076 | NCBI |
|  |  |  |  |  |  |  |  |  | GU471881 | NCBI |
|  |  |  |  |  |  |  |  |  | JF715004 | NCBI |
|  |  |  |  |  |  |  |  |  | JN802574 | NCBI |
|  |  |  |  |  |  |  |  |  | GU471886 | NCBI |
|  |  |  |  |  |  |  |  |  | JN802579 | NCBI |
|  |  |  |  |  |  |  |  |  | GU471883 | NCBI |
|  |  |  |  |  |  |  |  |  | KF259077 | NCBI |
|  |  |  |  |  |  |  |  |  | KF259083 | NCBI |
|  |  |  |  |  |  |  |  |  | KF259071 | NCBI |
|  |  |  |  |  |  |  |  |  | KF259101 | NCBI |
|  |  |  |  |  |  |  |  |  | GU471890 | NCBI |
|  |  |  |  |  |  |  |  |  | FJ231863 | NCBI |
|  |  |  |  |  |  |  |  |  | GQ463216 | NCBI |
|  |  |  |  |  |  |  |  |  | GU471891 | NCBI |
|  |  |  |  |  |  |  |  |  | GU471870 | NCBI |
|  |  |  |  |  |  |  |  |  | CY055156 | NCBI |
|  |  |  |  |  |  |  |  |  | KF259078 | NCBI |
|  |  |  |  |  |  |  |  |  | KF188397 | NCBI |
|  |  |  |  |  |  |  |  |  | KF259097 | NCBI |
|  |  |  |  |  |  |  |  |  | JN802577 | NCBI |
|  |  |  |  |  |  |  |  |  | JF916705 | NCBI |
|  |  |  |  |  |  |  |  |  | GU471879 | NCBI |
|  |  |  |  |  |  |  |  |  | JN802576 | NCBI |
|  |  |  |  |  |  |  |  |  | GU471878 | NCBI |
|  |  |  |  |  |  |  |  |  | KF188289 | NCBI |
|  |  |  |  |  |  |  |  |  | KF259102 | fludb |
|  |  |  |  |  |  |  |  |  | KF259103 | NCBI |
|  |  |  |  |  |  |  |  |  | GQ373075 | NCBI |
|  |  |  |  |  |  |  |  |  | FJ434578 | NCBI |
|  |  |  |  |  |  |  |  |  | GQ373068 | NCBI |
|  |  |  |  |  |  |  |  |  | GU722367 | NCBI |
|  |  |  |  |  |  |  |  |  | CY023832 | NCBI |
|  |  |  |  |  |  |  |  |  | GU722365 | NCBI |
|  |  |  |  |  |  |  |  |  | EU216090 | NCBI |
|  |  |  |  |  |  |  |  |  | GQ373069 | NCBI |
|  |  |  |  |  |  |  |  |  | CY023784 | NCBI |
|  |  |  |  |  |  |  |  |  | FJ231867 | NCBI |
|  |  |  |  |  |  |  |  |  | CY023736 | NCBI |
|  |  |  |  |  |  |  |  |  | KF259068 | NCBI |
|  |  |  |  |  |  |  |  |  | GQ373082 | NCBI |
|  |  |  |  |  |  |  |  |  | GQ373084 | fludb |
|  |  |  |  |  |  |  |  |  | GU722366 | NCBI |
|  |  |  |  |  |  |  |  |  | FJ190146 | NCBI |
|  |  |  |  |  |  |  |  |  | GQ373071 | NCBI |
|  |  |  |  |  |  |  |  |  | FJ190116 | NCBI |
|  |  |  |  |  |  |  |  |  | FJ581431 | NCBI |
|  |  |  |  |  |  |  |  |  | CY023816 | NCBI |
|  |  |  |  |  |  |  |  |  | FJ434576 | NCBI |
|  |  |  |  |  |  |  |  |  | CY023744 | NCBI |
|  |  |  |  |  |  |  |  |  | GQ373073 | NCBI |
|  |  |  |  |  |  |  |  |  | JN802658 | NCBI |
|  |  |  |  |  |  |  |  |  | FJ434562 | NCBI |
|  |  |  |  |  |  |  |  |  | KF259072 | NCBI |
|  |  |  |  |  |  |  |  |  | HQ398332 | NCBI |
|  |  |  |  |  |  |  |  |  | FJ231866 | NCBI |
|  |  |  |  |  |  |  |  |  | GU722362 | NCBI |
|  |  |  |  |  |  |  |  |  | FJ434565 | NCBI |
|  |  |  |  |  |  |  |  |  | GQ373081 | NCBI |
|  |  |  |  |  |  |  |  |  | EU086245 | NCBI |
|  |  |  |  |  |  |  |  |  | GU722360 | NCBI |
|  |  |  |  |  |  |  |  |  | CY023792 | NCBI |
|  |  |  |  |  |  |  |  |  | GQ373083 | NCBI |
|  |  |  |  |  |  |  |  |  | CY023824 | NCBI |
|  |  |  |  |  |  |  |  |  | EU644487 | fludb |
|  |  |  |  |  |  |  |  |  | JN804222 | NCBI |
|  |  |  |  |  |  |  |  |  | EU216092 | NCBI |
|  |  |  |  |  |  |  |  |  | JN804219 | fludb |
|  |  |  |  |  |  |  |  |  | KF259115 | NCBI |
|  |  |  |  |  |  |  |  |  | KF259108 | NCBI |
|  |  |  |  |  |  |  |  |  | HQ398333 | NCBI |
|  |  |  |  |  |  |  |  |  | EU216087 | NCBI |
|  |  |  |  |  |  |  |  |  | JN804208 | NCBI |
|  |  |  |  |  |  |  |  |  | FJ434577 | NCBI |
|  |  |  |  |  |  |  |  |  | JN804226 | NCBI |
|  |  |  |  |  |  |  |  |  | JN804209 | NCBI |
|  |  |  |  |  |  |  |  |  | EU216091 | NCBI |
|  |  |  |  |  |  |  |  |  | HQ398336 | NCBI |
|  |  |  |  |  |  |  |  |  | GU471888 | NCBI |
|  |  |  |  |  |  |  |  |  | EU216092 | fludb |
|  |  |  |  |  |  |  |  |  | HQ398338 | NCBI |
|  |  |  |  |  |  |  |  |  | GU471887 | NCBI |
|  |  |  |  |  |  |  |  |  | JN804210 | NCBI |
|  |  |  |  |  |  |  |  |  | HQ398335 | NCBI |
|  |  |  |  |  |  |  |  |  | GU471877 | NCBI |
|  |  |  |  |  |  |  |  |  | JN804225 | NCBI |
|  |  |  |  |  |  |  |  |  | JN804218 | fludb |
|  |  |  |  |  |  |  |  |  | GU471880 | NCBI |
|  |  |  |  |  |  |  |  |  | HQ398331 | NCBI |
|  |  |  |  |  |  |  |  |  | EU644484 | fludb |
|  |  |  |  |  |  |  |  |  | KF259116 | NCBI |
|  |  |  |  |  |  |  |  |  | GU471876 | NCBI |
|  |  |  |  |  |  |  |  |  | CY023840 | NCBI |
|  |  |  |  |  |  |  |  |  | EU644482 | NCBI |
|  |  |  |  |  |  |  |  |  | HQ398349 | NCBI |
|  |  |  |  |  |  |  |  |  | FJ434561 | NCBI |
|  |  |  |  |  |  |  |  |  | HQ398334 | NCBI |
|  |  |  |  |  |  |  |  |  | EU216089 | NCBI |
|  |  |  |  |  |  |  |  |  | JN802654 | NCBI |
|  |  |  |  |  |  |  |  |  | JX448748 | NCBI |
|  |  |  |  |  |  |  |  |  | FJ434564 | NCBI |
|  |  |  |  |  |  |  |  |  | FJ807712 | NCBI |
|  |  |  |  |  |  |  |  |  | JN802655 | NCBI |
|  |  |  |  |  |  |  |  |  | JN802618 | NCBI |
|  |  |  |  |  |  |  |  |  | JN802656 | NCBI |
|  |  |  |  |  |  |  |  |  | JN802661 | NCBI |
|  |  |  |  |  |  |  |  |  | GQ463213 | NCBI |
|  |  |  |  |  |  |  |  |  | JN802659 | NCBI |
|  |  |  |  |  |  |  |  |  | JN802607 | NCBI |
|  |  |  |  |  |  |  |  |  | JN802637 | NCBI |
|  |  |  |  |  |  |  |  |  | JN802643 | NCBI |
|  |  |  |  |  |  |  |  |  | JN802634 | NCBI |
|  |  |  |  |  |  |  |  |  | GQ463214 | NCBI |
|  |  |  |  |  |  |  |  |  | FJ434563 | NCBI |
|  |  |  |  |  |  |  |  |  | GU471882 | NCBI |
|  |  |  |  |  |  |  |  |  | JN802641 | NCBI |
|  |  |  |  |  |  |  |  |  | GQ463227 | NCBI |
|  |  |  |  |  |  |  |  |  | GQ463226 | NCBI |
|  |  |  |  |  |  |  |  |  | JN802651 | fludb |
|  |  |  |  |  |  |  |  |  | JN802645 | fludb |
|  |  |  |  |  |  |  |  |  | JN802632 | fludb |
|  |  |  |  |  |  |  |  |  | JN802652 | NCBI |
|  |  |  |  |  |  |  |  |  | JN802633 | NCBI |
|  |  |  |  |  |  |  |  |  | JN802612 | fludb |
|  |  |  |  |  |  |  |  |  | JN802653 | NCBI |
|  |  |  |  |  |  |  |  |  | JN802648 | NCBI |
|  |  |  |  |  |  |  |  |  | GQ463212 | NCBI |
|  |  |  |  |  |  |  |  |  | JN653615 | NCBI |
|  |  |  |  |  |  |  |  |  | JN802639 | NCBI |
|  |  |  |  |  |  |  |  |  | JN683646 | NCBI |
|  |  |  |  |  |  |  |  |  | JN802635 | NCBI |
|  |  |  |  |  |  |  |  |  | JN986884 | NCBI |
|  |  |  |  |  |  |  |  |  | JN653614 | NCBI |
|  |  |  |  |  |  |  |  |  | JX448754 | NCBI |
|  |  |  |  |  |  |  |  |  | JN802620 | fludb |
|  |  |  |  |  |  |  |  |  | JN804524 | fludb |
|  |  |  |  |  |  |  |  |  | JN804525 | NCBI |
|  |  |  |  |  |  |  |  |  | JN803971 | NCBI |
|  |  |  |  |  |  |  |  |  | KC817004 | NCBI |
|  |  |  |  |  |  |  |  |  | JN803974 | NCBI |
|  |  |  |  |  |  |  |  |  | JN802617 | NCBI |
|  |  |  |  |  |  |  |  |  | JN803970 | NCBI |
|  |  |  |  |  |  |  |  |  | JN803975 | NCBI |
|  |  |  |  |  |  |  |  |  | JN803976 | NCBI |
|  |  |  |  |  |  |  |  |  | JN804523 | NCBI |
|  |  |  |  |  |  |  |  |  | JN802619 | fludb |
|  |  |  |  |  |  |  |  |  | JN802605 | fludb |
|  |  |  |  |  |  |  |  |  | JX448755 | NCBI |
|  |  |  |  |  |  |  |  |  | JN804522 | NCBI |
|  |  |  |  |  |  |  |  |  | JN804519 | NCBI |
|  |  |  |  |  |  |  |  |  | JN804020 | NCBI |
|  |  |  |  |  |  |  |  |  | JX448753 | NCBI |
|  |  |  |  |  |  |  |  |  | JN804518 | NCBI |
|  |  |  |  |  |  |  |  |  | JN804520 | NCBI |
|  |  |  |  |  |  |  |  |  | JN803973 | NCBI |
|  |  |  |  |  |  |  |  |  | JN803977 | NCBI |
|  |  |  |  |  |  |  |  |  | JN802595 | NCBI |
|  |  |  |  |  |  |  |  |  | KC820998 | NCBI |
|  |  |  |  |  |  |  |  |  | JX448756 | NCBI |
|  |  |  |  |  |  |  |  |  | KC817006 | NCBI |
|  |  |  |  |  |  |  |  |  | JF714997 | NCBI |
|  |  |  |  |  |  |  |  |  | JN804515 | NCBI |
|  |  |  |  |  |  |  |  |  | JN804087 | fludb |
|  |  |  |  |  |  |  |  |  | JN804079 | NCBI |
|  |  |  |  |  |  |  |  |  | FJ434579 | NCBI |
|  |  |  |  |  |  |  |  |  | KF259111 | NCBI |
|  |  |  |  |  |  |  |  |  | JN804058 | NCBI |
|  |  |  |  |  |  |  |  |  | JN804366 | NCBI |
|  |  |  |  |  |  |  |  |  | JN804418 | NCBI |
|  |  |  |  |  |  |  |  |  | JN804368 | NCBI |
|  |  |  |  |  |  |  |  |  | JN804061 | fludb |
|  |  |  |  |  |  |  |  |  | JN804375 | fludb |
|  |  |  |  |  |  |  |  |  | JN804064 | NCBI |
|  |  |  |  |  |  |  |  |  | JN804085 | NCBI |
|  |  |  |  |  |  |  |  |  | JN804071 | NCBI |
|  |  |  |  |  |  |  |  |  | JF715026 | NCBI |
|  |  |  |  |  |  |  |  |  | FJ434583 | NCBI |
|  |  |  |  |  |  |  |  |  | KC767259 | NCBI |
|  |  |  |  |  |  |  |  |  | JN804065 | fludb |
|  |  |  |  |  |  |  |  |  | FJ434584 | NCBI |
|  |  |  |  |  |  |  |  |  | FJ434567 | NCBI |
|  |  |  |  |  |  |  |  |  | JN804067 | NCBI |
|  |  |  |  |  |  |  |  |  | JN802580 | NCBI |
|  |  |  |  |  |  |  |  |  | FJ434566 | NCBI |
|  |  |  |  |  |  |  |  |  | JN804086 | NCBI |
|  |  |  |  |  |  |  |  |  | KF259109 | NCBI |
|  |  |  |  |  |  |  |  |  | JN804073 | NCBI |
|  |  |  |  |  |  |  |  |  | KF259110 | NCBI |
|  |  |  |  |  |  |  |  |  | JN804369 | NCBI |
|  |  |  |  |  |  |  |  |  | JN804069 | NCBI |
|  |  |  |  |  |  |  |  |  | JN804516 | NCBI |
|  |  |  |  |  |  |  |  |  | JN804070 | NCBI |
|  |  |  |  |  |  |  |  |  | JN802582 | NCBI |
|  |  |  |  |  |  |  |  |  | JN804080 | NCBI |
|  |  |  |  |  |  |  |  |  | FJ434568 | NCBI |
|  |  |  |  |  |  |  |  |  | FJ434581 | NCBI |
|  |  |  |  |  |  |  |  |  | JN804051 | NCBI |
|  |  |  |  |  |  |  |  |  | JN804059 | NCBI |
|  |  |  |  |  |  |  |  |  | JN804057 | NCBI |
|  |  |  |  |  |  |  |  |  | JN804050 | NCBI |
|  |  |  |  |  |  |  |  |  | KC821000 | NCBI |
|  |  |  |  |  |  |  |  |  | JN804533 | NCBI |
|  |  |  |  |  |  |  |  |  | JN804077 | NCBI |
|  |  |  |  |  |  |  |  |  | JF715036 | NCBI |
|  |  |  |  |  |  |  |  |  | KC820999 | NCBI |
|  |  |  |  |  |  |  |  |  | JN804534 | NCBI |
|  |  |  |  |  |  |  |  |  | KC821010 | NCBI |
|  |  |  |  |  |  |  |  |  | JN804082 | NCBI |
|  |  |  |  |  |  |  |  |  | JX448746 | NCBI |
|  |  |  |  |  |  |  |  |  | JN804055 | NCBI |
|  |  |  |  |  |  |  |  |  | JX448765 | NCBI |
|  |  |  |  |  |  |  |  |  | KC920695 | NCBI |
|  |  |  |  |  |  |  |  |  | JF715032 | NCBI |
|  |  |  |  |  |  |  |  |  | JN804053 | NCBI |
|  |  |  |  |  |  |  |  |  | JN804083 | NCBI |
|  |  |  |  |  |  |  |  |  | JN804081 | NCBI |
|  |  |  |  |  |  |  |  |  | JN804054 | NCBI |
|  |  |  |  |  |  |  |  |  | JF715033 | NCBI |
|  |  |  |  |  |  |  |  |  | KC821009 | NCBI |
|  |  |  |  |  |  |  |  |  | JN804532 | NCBI |
|  |  |  |  |  |  |  |  |  | KC821004 | NCBI |
|  |  |  |  |  |  |  |  |  | JN986886 | NCBI |
|  |  |  |  |  |  |  |  |  | JN804052 | NCBI |
|  |  |  |  |  |  |  |  |  | KC821003 | NCBI |
|  |  |  |  |  |  |  |  |  | JN804535 | NCBI |
|  |  |  |  |  |  |  |  |  | JN804078 | NCBI |
|  |  |  |  |  |  |  |  |  | GQ373080 | NCBI |
|  |  |  |  |  |  |  |  |  | FJ190137 | NCBI |
|  |  |  |  |  |  |  |  |  | JN802533 | fludb |
|  |  |  |  |  |  |  |  |  | GU474590 | NCBI |
|  |  |  |  |  |  |  |  |  | GU474550 | NCBI |
|  |  |  |  |  |  |  |  |  | GU474582 | NCBI |
|  |  |  |  |  |  |  |  |  | GQ373078 | NCBI |
|  |  |  |  |  |  |  |  |  | JN802543 | fludb |
|  |  |  |  |  |  |  |  |  | JN802529 | NCBI |
|  |  |  |  |  |  |  |  |  | GQ463220 | NCBI |
|  |  |  |  |  |  |  |  |  | GU474574 | NCBI |
|  |  |  |  |  |  |  |  |  | JN802534 | NCBI |
|  |  |  |  |  |  |  |  |  | GQ373079 | NCBI |
|  |  |  |  |  |  |  |  |  | JN802536 | NCBI |
|  |  |  |  |  |  |  |  |  | JN802547 | NCBI |
|  |  |  |  |  |  |  |  |  | GQ463219 | NCBI |
|  |  |  |  |  |  |  |  |  | GU474566 | NCBI |
|  |  |  |  |  |  |  |  |  | GQ373077 | NCBI |
|  |  |  |  |  |  |  |  |  | JN802530 | NCBI |
|  |  |  |  |  |  |  |  |  | JN802548 | NCBI |
|  |  |  |  |  |  |  |  |  | KC768039 | NCBI |
|  |  |  |  |  |  |  |  |  | GQ373076 | NCBI |
|  |  |  |  |  |  |  |  |  | GU471895 | NCBI |
|  |  |  |  |  |  |  |  |  | JN683644 | NCBI |
|  |  |  |  |  |  |  |  |  | KF746769 | NCBI |
|  |  |  |  |  |  |  |  |  | KC821001 | NCBI |
|  |  |  |  |  |  |  |  |  | JF519769 | NCBI |
|  |  |  |  |  |  |  |  |  | HM751194 | NCBI |
|  |  |  |  |  |  |  |  |  | KF746748 | NCBI |
|  |  |  |  |  |  |  |  |  | HM751186 | NCBI |
|  |  |  |  |  |  |  |  |  | KF715228 | NCBI |
|  |  |  |  |  |  |  |  |  | KF746819 | NCBI |
|  |  |  |  |  |  |  |  |  | GU471805 | NCBI |
|  |  |  |  |  |  |  |  |  | HM751154 | NCBI |
|  |  |  |  |  |  |  |  |  | JN683645 | NCBI |
|  |  |  |  |  |  |  |  |  | GU471799 | NCBI |
|  |  |  |  |  |  |  |  |  | HM773437 | NCBI |
|  |  |  |  |  |  |  |  |  | GU471796 | NCBI |
|  |  |  |  |  |  |  |  |  | GU471894 | NCBI |
|  |  |  |  |  |  |  |  |  | GU471800 | NCBI |
|  |  |  |  |  |  |  |  |  | GQ373072 | NCBI |
|  |  |  |  |  |  |  |  |  | GU471795 | NCBI |
|  |  |  |  |  |  |  |  |  | JN683647 | NCBI |
|  |  |  |  |  |  |  |  |  | GU471896 | NCBI |
|  |  |  |  |  |  |  |  |  | GU471893 | NCBI |
|  |  |  |  |  |  |  |  |  | FJ190138 | NCBI |
|  |  |  |  |  |  |  |  |  | GU471802 | NCBI |
|  |  |  |  |  |  |  |  |  | JF795054 | NCBI |
|  |  |  |  |  |  |  |  |  | JF795038 | NCBI |
|  |  |  |  |  |  |  |  |  | KJ419942 | NCBI |
|  |  |  |  |  |  |  |  |  | GQ463222 | NCBI |
|  |  |  |  |  |  |  |  |  | KJ419948 | NCBI |
|  |  |  |  |  |  |  |  |  | KJ419946 | NCBI |
|  |  |  |  |  |  |  |  |  | JF795046 | NCBI |
|  |  |  |  |  |  |  |  |  | JF795144 | NCBI |
|  |  |  |  |  |  |  |  |  | JN804542 | NCBI |
|  |  |  |  |  |  |  |  |  | GQ373074 | NCBI |
|  |  |  |  |  |  |  |  |  | CY087187 | NCBI |
|  |  |  |  |  |  |  |  |  | KJ419939 | NCBI |
|  |  |  |  |  |  |  |  |  | KC821007 | NCBI |
|  |  |  |  |  |  |  |  |  | CY087171 | NCBI |
|  |  |  |  |  |  |  |  |  | GQ463223 | NCBI |
|  |  |  |  |  |  |  |  |  | GQ463221 | NCBI |
|  |  |  |  |  |  |  |  |  | KC779047 | NCBI |
|  |  |  |  |  |  |  |  |  | GQ463217 | NCBI |
|  |  |  |  |  |  |  |  |  | KJ419953 | NCBI |
|  |  |  |  |  |  |  |  |  | KJ419945 | NCBI |
|  |  |  |  |  |  |  |  |  | KC821005 | NCBI |
|  |  |  |  |  |  |  |  |  | KJ419943 | NCBI |
|  |  |  |  |  |  |  |  |  | KJ419944 | NCBI |
|  |  |  |  |  |  |  |  |  | JN804001 | NCBI |
|  |  |  |  |  |  |  |  |  | GQ463218 | NCBI |
|  |  |  |  |  |  |  |  |  | KC768040 | NCBI |
|  |  |  |  |  |  |  |  |  | GQ463224 | NCBI |
|  |  |  |  |  |  |  |  |  | KC768041 | NCBI |
|  |  |  |  |  |  |  |  |  | CY087179 | NCBI |
|  |  |  |  |  |  |  |  |  | HM998914 | NCBI |
|  |  |  |  |  |  |  |  |  | JN986885 | NCBI |
|  |  |  |  |  |  |  |  |  | JN802660 | NCBI |
|  |  |  |  |  |  |  |  |  | JN804548 | fludb |
|  |  |  |  |  |  |  |  |  | JN804550 | fludb |
|  |  |  |  |  |  |  |  |  | JN802557 | NCBI |
|  |  |  |  |  |  |  |  |  | JN653613 | NCBI |
|  |  |  |  |  |  |  |  |  | JN802556 | fludb |
|  |  |  |  |  |  |  |  |  | JF715003 | NCBI |
|  |  |  |  |  |  |  |  |  | JN653619 | NCBI |
|  |  |  |  |  |  |  |  |  | JN804348 | NCBI |
|  |  |  |  |  |  |  |  |  | JN804551 | NCBI |
|  |  |  |  |  |  |  |  |  | JN802551 | NCBI |
|  |  |  |  |  |  |  |  |  | JN804349 | NCBI |
|  |  |  |  |  |  |  |  |  | JN804547 | NCBI |
|  |  |  |  |  |  |  |  |  | HM998922 | NCBI |
|  |  |  |  |  |  |  |  |  | JF715043 | NCBI |
|  |  |  |  |  |  |  |  |  | JN804334 | NCBI |
|  |  |  |  |  |  |  |  |  | JN804185 | NCBI |
|  |  |  |  |  |  |  |  |  | JN804363 | NCBI |
|  |  |  |  |  |  |  |  |  | JN804544 | NCBI |
|  |  |  |  |  |  |  |  |  | JN804358 | fludb |
|  |  |  |  |  |  |  |  |  | JF715006 | NCBI |
|  |  |  |  |  |  |  |  |  | JN804354 | NCBI |
|  |  |  |  |  |  |  |  |  | JN802562 | NCBI |
|  |  |  |  |  |  |  |  |  | JN804364 | fludb |
|  |  |  |  |  |  |  |  |  | JN804552 | NCBI |
|  |  |  |  |  |  |  |  |  | JN804353 | NCBI |
|  |  |  |  |  |  |  |  |  | JN802550 | NCBI |
|  |  |  |  |  |  |  |  |  | JN804546 | NCBI |
|  |  |  |  |  |  |  |  |  | JF715000 | NCBI |
|  |  |  |  |  |  |  |  |  | JN804337 | NCBI |
|  |  |  |  |  |  |  |  |  | JN653618 | NCBI |
|  |  |  |  |  |  |  |  |  | JN802662 | fludb |
|  |  |  |  |  |  |  |  |  | JN804037 | NCBI |
|  |  |  |  |  |  |  |  |  | JN803988 | NCBI |
|  |  |  |  |  |  |  |  |  | JN986890 | NCBI |
|  |  |  |  |  |  |  |  |  | KC821011 | NCBI |
|  |  |  |  |  |  |  |  |  | JN804276 | fludb |
|  |  |  |  |  |  |  |  |  | JN804381 | fludb |
|  |  |  |  |  |  |  |  |  | JX448751 | NCBI |
|  |  |  |  |  |  |  |  |  | KC821008 | NCBI |
|  |  |  |  |  |  |  |  |  | JN804234 | NCBI |
|  |  |  |  |  |  |  |  |  | JN653608 | NCBI |
|  |  |  |  |  |  |  |  |  | KC821008 | NCBI |
|  |  |  |  |  |  |  |  |  | JN804041 | fludb |
|  |  |  |  |  |  |  |  |  | JN804038 | NCBI |
|  |  |  |  |  |  |  |  |  | KF638574 | NCBI |
|  |  |  |  |  |  |  |  |  | JN804233 | NCBI |
|  |  |  |  |  |  |  |  |  | JN804272 | NCBI |
|  |  |  |  |  |  |  |  |  | KC821002 | NCBI |
|  |  |  |  |  |  |  |  |  | JN804231 | NCBI |
|  |  |  |  |  |  |  |  |  | JN804039 | NCBI |
|  |  |  |  |  |  |  |  |  | JN803992 | fludb |
|  |  |  |  |  |  |  |  |  | JN804280 | NCBI |
|  |  |  |  |  |  |  |  |  | KC871564 | NCBI |
|  |  |  |  |  |  |  |  |  | JX448763 | NCBI |
|  |  |  |  |  |  |  |  |  | KC821006 | NCBI |
|  |  |  |  |  |  |  |  |  | JN804003 | NCBI |
|  |  |  |  |  |  |  |  |  | JN804316 | NCBI |
|  |  |  |  |  |  |  |  |  | JN804046 | NCBI |
|  |  |  |  |  |  |  |  |  | JN804352 | NCBI |
|  |  |  |  |  |  |  |  |  | JN804047 | fludb |
|  |  |  |  |  |  |  |  |  | JX448752 | NCBI |
|  |  |  |  |  |  |  |  |  | JN804035 | NCBI |
|  |  |  |  |  |  |  |  |  | JN804313 | NCBI |
|  |  |  |  |  |  |  |  |  | JN804360 | fludb |
|  |  |  |  |  |  |  |  |  | JQ356874 | NCBI |
|  |  |  |  |  |  |  |  |  | JN804095 | NCBI |
|  |  |  |  |  |  |  |  |  | JX312542 | NCBI |
|  |  |  |  |  |  |  |  |  | JN804116 | NCBI |
|  |  |  |  |  |  |  |  |  | JN804008 | NCBI |
|  |  |  |  |  |  |  |  |  | JN804187 | NCBI |
|  |  |  |  |  |  |  |  |  | JN804192 | fludb |
|  |  |  |  |  |  |  |  |  | JN804451 | NCBI |
|  |  |  |  |  |  |  |  |  | JN804096 | NCBI |
|  |  |  |  |  |  |  |  |  | JN804145 | NCBI |
|  |  |  |  |  |  |  |  |  | JN804133 | NCBI |
|  |  |  |  |  |  |  |  |  | JN804454 | NCBI |
|  |  |  |  |  |  |  |  |  | JN804182 | NCBI |
|  |  |  |  |  |  |  |  |  | JN804093 | NCBI |
|  |  |  |  |  |  |  |  |  | JN803961 | NCBI |
|  |  |  |  |  |  |  |  |  | JN804043 | NCBI |
|  |  |  |  |  |  |  |  |  | JN804094 | NCBI |
|  |  |  |  |  |  |  |  |  | JN804092 | NCBI |
|  |  |  |  |  |  |  |  |  | JN653620 | NCBI |
|  |  |  |  |  |  |  |  |  | JN804090 | NCBI |
|  |  |  |  |  |  |  |  |  | JN803947 | NCBI |
|  |  |  |  |  |  |  |  |  | JN804017 | fludb |
|  |  |  |  |  |  |  |  |  | JN804183 | NCBI |
|  |  |  |  |  |  |  |  |  | JN804511 | NCBI |
|  |  |  |  |  |  |  |  |  | JN804184 | fludb |
|  |  |  |  |  |  |  |  |  | JN653617 | NCBI |
|  |  |  |  |  |  |  |  |  | JF906206 | NCBI |
|  |  |  |  |  |  |  |  |  | JN804513 | NCBI |
|  |  |  |  |  |  |  |  |  | JN804196 | fludb |
|  |  |  |  |  |  |  |  |  | JN803964 | NCBI |
|  |  |  |  |  |  |  |  |  | JN803957 | NCBI |
|  |  |  |  |  |  |  |  |  | JN804132 | fludb |
|  |  |  |  |  |  |  |  |  | JN804131 | fludb |
|  |  |  |  |  |  |  |  |  | JN804091 | NCBI |
|  |  |  |  |  |  |  |  |  | JN804252 | NCBI |
|  |  |  |  |  |  |  |  |  | JN804152 | NCBI |
|  |  |  |  |  |  |  |  |  | JN653610 | NCBI |
|  |  |  |  |  |  |  |  |  | JN804150 | NCBI |
|  |  |  |  |  |  |  |  |  | JN653606 | NCBI |
|  |  |  |  |  |  |  |  |  | JF795070 | NCBI |
|  |  |  |  |  |  |  |  |  | JN653609 | NCBI |
|  |  |  |  |  |  |  |  |  | KF835986 | NCBI |
|  |  |  |  |  |  |  |  |  | KJ419940 | NCBI |
|  |  |  |  |  |  |  |  |  | JF795102 | NCBI |
|  |  |  |  |  |  |  |  |  | JN804347 | NCBI |
|  |  |  |  |  |  |  |  |  | JN804343 | NCBI |
|  |  |  |  |  |  |  |  |  | JN804514 | NCBI |
|  |  |  |  |  |  |  |  |  | JN804158 | NCBI |
|  |  |  |  |  |  |  |  |  | JF715018 | NCBI |
|  |  |  |  |  |  |  |  |  | JN804256 | NCBI |
|  |  |  |  |  |  |  |  |  | JN804121 | NCBI |
|  |  |  |  |  |  |  |  |  | JN804355 | NCBI |
|  |  |  |  |  |  |  |  |  | JN804356 | NCBI |
|  |  |  |  |  |  |  |  |  | JN804179 | fludb |
|  |  |  |  |  |  |  |  |  | JN653612 | NCBI |
|  |  |  |  |  |  |  |  |  | KC821012 | NCBI |
|  |  |  |  |  |  |  |  |  | JN804530 | NCBI |
|  |  |  |  |  |  |  |  |  | KC821013 | NCBI |
|  |  |  |  |  |  |  |  |  | JN804367 | NCBI |
|  |  |  |  |  |  |  |  |  | JN804119 | NCBI |
|  |  |  |  |  |  |  |  |  | KC821024 | NCBI |
|  |  |  |  |  |  |  |  |  | JN804259 | NCBI |
|  |  |  |  |  |  |  |  |  | JN804117 | NCBI |
|  |  |  |  |  |  |  |  |  | JN804357 | NCBI |
|  |  |  |  |  |  |  |  |  | JN802573 | NCBI |
|  |  |  |  |  |  |  |  |  | JN804253 | fludb |
|  |  |  |  |  |  |  |  |  | JN804267 | NCBI |
|  |  |  |  |  |  |  |  |  | JN804391 | NCBI |
|  |  |  |  |  |  |  |  |  | JN804309 | NCBI |
|  |  |  |  |  |  |  |  |  | JN804242 | NCBI |
|  |  |  |  |  |  |  |  |  | JN804416 | NCBI |
|  |  |  |  |  |  |  |  |  | JN804415 | NCBI |
|  |  |  |  |  |  |  |  |  | JN804420 | NCBI |
|  |  |  |  |  |  |  |  |  | JN804344 | NCBI |
|  |  |  |  |  |  |  |  |  | KF514116 | NCBI |
|  |  |  |  |  |  |  |  |  | JN804447 | NCBI |
|  |  |  |  |  |  |  |  |  | JN804389 | fludb |
|  |  |  |  |  |  |  |  |  | JN804237 | fludb |
|  |  |  |  |  |  |  |  |  | JN804421 | NCBI |
|  |  |  |  |  |  |  |  |  | JN804361 | NCBI |
|  |  |  |  |  |  |  |  |  | JN804446 | NCBI |
|  |  |  |  |  |  |  |  |  | JN804431 | fludb |
|  |  |  |  |  |  |  |  |  | JN804244 | NCBI |
|  |  |  |  |  |  |  |  |  | JN804430 | NCBI |
|  |  |  |  |  |  |  |  |  | JN804422 | NCBI |
|  |  |  |  |  |  |  |  |  | JN804435 | fludb |
|  |  |  |  |  |  |  |  |  | JN804434 | fludb |
|  |  |  |  |  |  |  |  |  | JN804362 | NCBI |
|  |  |  |  |  |  |  |  |  | JN804385 | fludb |
|  |  |  |  |  |  |  |  |  | KC821021 | NCBI |
|  |  |  |  |  |  |  |  |  | JN804243 | NCBI |
|  |  |  |  |  |  |  |  |  | JN804419 | NCBI |
|  |  |  |  |  |  |  |  |  | JN804241 | NCBI |
|  |  |  |  |  |  |  |  |  | JN804401 | NCBI |
|  |  |  |  |  |  |  |  |  | JN804307 | NCBI |
|  |  |  |  |  |  |  |  |  | JN804445 | NCBI |
|  |  |  |  |  |  |  |  |  | JN804380 | NCBI |
|  |  |  |  |  |  |  |  |  | JN804236 | NCBI |
|  |  |  |  |  |  |  |  |  | KC821022 | NCBI |
|  |  |  |  |  |  |  |  |  | JN802563 | NCBI |
|  |  |  |  |  |  |  |  |  | JN802564 | NCBI |
|  |  |  |  |  |  |  |  |  | JN804331 | fludb |
|  |  |  |  |  |  |  |  |  | JN804024 | NCBI |
|  |  |  |  |  |  |  |  |  | JN804324 | NCBI |
|  |  |  |  |  |  |  |  |  | JN804294 | NCBI |
|  |  |  |  |  |  |  |  |  | JN804539 | NCBI |
|  |  |  |  |  |  |  |  |  | JN986887 | NCBI |
|  |  |  |  |  |  |  |  |  | JN804089 | NCBI |
|  |  |  |  |  |  |  |  |  | JN804290 | NCBI |
|  |  |  |  |  |  |  |  |  | JN802569 | NCBI |
|  |  |  |  |  |  |  |  |  | JQ770129 | NCBI |
|  |  |  |  |  |  |  |  |  | JN804305 | NCBI |
|  |  |  |  |  |  |  |  |  | JF715045 | NCBI |
|  |  |  |  |  |  |  |  |  | JN804230 | NCBI |
|  |  |  |  |  |  |  |  |  | JN804088 | NCBI |
|  |  |  |  |  |  |  |  |  | JN804026 | NCBI |
|  |  |  |  |  |  |  |  |  | JF715005 | NCBI |
|  |  |  |  |  |  |  |  |  | JN804232 | fludb |
|  |  |  |  |  |  |  |  |  | JN986888 | NCBI |
|  |  |  |  |  |  |  |  |  | JN804536 | NCBI |
|  |  |  |  |  |  |  |  |  | JN804304 | NCBI |
|  |  |  |  |  |  |  |  |  | JN804291 | NCBI |
|  |  |  |  |  |  |  |  |  | JN804030 | NCBI |
|  |  |  |  |  |  |  |  |  | JN802567 | NCBI |
|  |  |  |  |  |  |  |  |  | JN804297 | NCBI |
|  |  |  |  |  |  |  |  |  | JN804032 | NCBI |
|  |  |  |  |  |  |  |  |  | JN802565 | NCBI |
|  |  |  |  |  |  |  |  |  | JN804292 | NCBI |
|  |  |  |  |  |  |  |  |  | JN804302 | NCBI |
|  |  |  |  |  |  |  |  |  | JN804540 | NCBI |
|  |  |  |  |  |  |  |  |  | JN804028 | NCBI |
|  |  |  |  |  |  |  |  |  | JN804031 | NCBI |
|  |  |  |  |  |  |  |  |  | JN802566 | NCBI |
|  |  |  |  |  |  |  |  |  | JN804295 | NCBI |
|  |  |  |  |  |  |  |  |  | JN804299 | NCBI |
|  |  |  |  |  |  |  |  |  | JN802571 | NCBI |
|  |  |  |  |  |  |  |  |  | JN803969 | NCBI |
|  |  |  |  |  |  |  |  |  | JN803979 | NCBI |
|  |  |  |  |  |  |  |  |  | JN803958 | fludb |
|  |  |  |  |  |  |  |  |  | JN802572 | NCBI |
|  |  |  |  |  |  |  |  |  | JN803985 | fludb |
|  |  |  |  |  |  |  |  |  | JN804075 | NCBI |
|  |  |  |  |  |  |  |  |  | GU471869 | NCBI |
|  |  |  |  |  |  |  |  |  | KF259128 | NCBI |
|  |  |  |  |  |  |  |  |  | JN803986 | NCBI |
|  |  |  |  |  |  |  |  |  | JN803951 | NCBI |
|  |  |  |  |  |  |  |  |  | KC821018 | NCBI |
|  |  |  |  |  |  |  |  |  | JN803955 | NCBI |
|  |  |  |  |  |  |  |  |  | JN803949 | NCBI |
|  |  |  |  |  |  |  |  |  | JN803956 | fludb |
|  |  |  |  |  |  |  |  |  | KF259129 | fludb |
|  |  |  |  |  |  |  |  |  | JN803984 | NCBI |
|  |  |  |  |  |  |  |  |  | KF259112 | NCBI |
|  |  |  |  |  |  |  |  |  | KF188401 | NCBI |
|  |  |  |  |  |  |  |  |  | JN803959 | NCBI |
|  |  |  |  |  |  |  |  |  | JN803960 | NCBI |
|  |  |  |  |  |  |  |  |  | JN803968 | NCBI |
|  |  |  |  |  |  |  |  |  | JF715007 | NCBI |
|  |  |  |  |  |  |  |  |  | JN804002 | NCBI |
|  |  |  |  |  |  |  |  |  | HM036348 | NCBI |
|  |  |  |  |  |  |  |  |  | GU471868 | NCBI |
|  |  |  |  |  |  |  |  |  | KC779048 | NCBI |
|  |  |  |  |  |  |  |  |  | JN804027 | fludb |
|  |  |  |  |  |  |  |  |  | GU471871 | NCBI |
|  |  |  |  |  |  |  |  |  | JN804021 | NCBI |
|  |  |  |  |  |  |  |  |  | JN803953 | NCBI |
|  |  |  |  |  |  |  |  |  | JN803978 | NCBI |
|  |  |  |  |  |  |  |  |  | GU471865 | NCBI |
|  |  |  |  |  |  |  |  |  | JN804013 | NCBI |
|  |  |  |  |  |  |  |  |  | GU471804 | NCBI |
|  |  |  |  |  |  |  |  |  | KF259114 | NCBI |
|  |  |  |  |  |  |  |  |  | JN804049 | fludb |
|  |  |  |  |  |  |  |  |  | JN803999 | NCBI |
|  |  |  |  |  |  |  |  |  | JN804019 | NCBI |
|  |  |  |  |  |  |  |  |  | KF259120 | fludb |
|  |  |  |  |  |  |  |  |  | GU471864 | fludb |
|  |  |  |  |  |  |  |  |  | KC817005 | NCBI |
|  |  |  |  |  |  |  |  |  | KF259119 | NCBI |
|  |  |  |  |  |  |  |  |  | JX448757 | NCBI |
|  |  |  |  |  |  |  |  |  | JN803998 | NCBI |
|  |  |  |  |  |  |  |  |  | GU471867 | NCBI |
|  |  |  |  |  |  |  |  |  | JN804365 | NCBI |
|  |  |  |  |  |  |  |  |  | JF715001 | NCBI |
|  |  |  |  |  |  |  |  |  | JF795086 | NCBI |
|  |  |  |  |  |  |  |  |  | GU471874 | NCBI |
|  |  |  |  |  |  |  |  |  | GU471872 | NCBI |
|  |  |  |  |  |  |  |  |  | KC821016 | NCBI |
|  |  |  |  |  |  |  |  |  | KC879302 | NCBI |
|  |  |  |  |  |  |  |  |  | JX448760 | NCBI |
|  |  |  |  |  |  |  |  |  | JN653607 | NCBI |
|  |  |  |  |  |  |  |  |  | JN803997 | fludb |
|  |  |  |  |  |  |  |  |  | JN804009 | NCBI |
|  |  |  |  |  |  |  |  |  | KF746851 | NCBI |
|  |  |  |  |  |  |  |  |  | KC821017 | NCBI |
|  |  |  |  |  |  |  |  |  | JF715009 | NCBI |
|  |  |  |  |  |  |  |  |  | JN803996 | fludb |
|  |  |  |  |  |  |  |  |  | KF259118 | NCBI |
|  |  |  |  |  |  |  |  |  | KC821023 | NCBI |
|  |  |  |  |  |  |  |  |  | JN804011 | NCBI |
|  |  |  |  |  |  |  |  |  | JN804010 | NCBI |
|  |  |  |  |  |  |  |  |  | GU471866 | NCBI |
|  |  |  |  |  |  |  |  |  | JN653611 | NCBI |
|  |  |  |  |  |  |  |  |  | JN804467 | NCBI |
|  |  |  |  |  |  |  |  |  | JN804463 | NCBI |
|  |  |  |  |  |  |  |  |  | JN804494 | NCBI |
|  |  |  |  |  |  |  |  |  | JN804460 | NCBI |
|  |  |  |  |  |  |  |  |  | JN804504 | NCBI |
|  |  |  |  |  |  |  |  |  | JN804473 | NCBI |
|  |  |  |  |  |  |  |  |  | KC821019 | NCBI |
|  |  |  |  |  |  |  |  |  | JN683642 | NCBI |
|  |  |  |  |  |  |  |  |  | JN804488 | NCBI |
|  |  |  |  |  |  |  |  |  | JN804469 | NCBI |
|  |  |  |  |  |  |  |  |  | HQ225839 | NCBI |
|  |  |  |  |  |  |  |  |  | JN804508 | fludb |
|  |  |  |  |  |  |  |  |  | JN804507 | fludb |
|  |  |  |  |  |  |  |  |  | JN683643 | NCBI |
|  |  |  |  |  |  |  |  |  | JN804034 | NCBI |
|  |  |  |  |  |  |  |  |  | JF715030 | NCBI |
|  |  |  |  |  |  |  |  |  | JN804471 | NCBI |
|  |  |  |  |  |  |  |  |  | JN653621 | NCBI |
|  |  |  |  |  |  |  |  |  | JN804505 | NCBI |
|  |  |  |  |  |  |  |  |  | JF715002 | NCBI |
|  |  |  |  |  |  |  |  |  | JN804042 | NCBI |
|  |  |  |  |  |  |  |  |  | JN804489 | NCBI |
|  |  |  |  |  |  |  |  |  | HQ378727 | NCBI |
|  |  |  |  |  |  |  |  |  | JN653616 | NCBI |
|  |  |  |  |  |  |  |  |  | JF795094 | NCBI |
|  |  |  |  |  |  |  |  |  | JN804499 | NCBI |
|  |  |  |  |  |  |  |  |  | JN804464 | NCBI |
|  |  |  |  |  |  |  |  |  | JF715037 | NCBI |
|  |  |  |  |  |  |  |  |  | KC821025 | NCBI |
|  |  |  |  |  |  |  |  |  | JN804506 | NCBI |
|  |  |  |  |  |  |  |  |  | JN804501 | NCBI |
|  |  |  |  |  |  |  |  |  | JN804497 | NCBI |
|  |  |  |  |  |  |  |  |  | JN804458 | NCBI |
|  |  |  |  |  |  |  |  |  | KC821014 | NCBI |
|  |  |  |  |  |  |  |  |  | JN804456 | NCBI |
|  |  |  |  |  |  |  |  |  | JQ356873 | NCBI |
|  |  |  |  |  |  |  |  |  | JQ770126 | NCBI |
|  |  |  |  |  |  |  |  |  | JQ770125 | NCBI |
|  |  |  |  |  |  |  |  |  | JN804479 | fludb |
|  |  |  |  |  |  |  |  |  | KC821015 | NCBI |
|  |  |  |  |  |  |  |  |  | KC920698 | NCBI |
|  |  |  |  |  |  |  |  |  | JX448766 | NCBI |
|  |  |  |  |  |  |  |  |  | KC821026 | NCBI |
|  |  |  |  |  |  |  |  |  | JN804480 | NCBI |
|  |  |  |  |  |  |  |  |  | JQ770124 | NCBI |
|  |  |  |  |  |  |  |  |  | KC464598 | NCBI |
|  |  |  |  |  |  |  |  |  | JQ770127 | NCBI |
|  |  |  |  |  |  |  |  |  | KC821020 | NCBI |
|  |  |  |  |  |  |  |  |  | JQ770139 | NCBI |
|  |  |  |  |  |  |  |  |  | JQ770142 | NCBI |
|  |  |  |  |  |  |  |  |  | JQ770128 | NCBI |
|  |  |  |  |  |  |  |  |  | KF059274 | NCBI |
|  |  |  |  |  |  |  |  |  | KF059275 | NCBI |
|  |  |  |  |  |  |  |  |  | JN804477 | NCBI |
|  |  |  |  |  |  |  |  |  | KF059276 | NCBI |
|  |  |  |  |  |  |  |  |  | KF059277 | NCBI |
|  |  |  |  |  |  |  |  |  | KJ419950 | NCBI |
|  |  |  |  |  |  |  |  |  | KF059278 | NCBI |
|  |  |  |  |  |  |  |  |  | JQ770143 | NCBI |
|  |  |  |  |  |  |  |  |  | KF059279 | NCBI |
|  |  |  |  |  |  |  |  |  | JQ770138 | NCBI |
|  |  |  |  |  |  |  |  |  | KF059280 | NCBI |
|  |  |  |  |  |  |  |  |  | KF059281 | NCBI |
|  |  |  |  |  |  |  |  |  | KC920694 | NCBI |
|  |  |  |  |  |  |  |  |  | KF835982 | NCBI |
|  |  |  |  |  |  |  |  |  | JX448767 | fludb |
|  |  |  |  |  |  |  |  |  | KC920692 | NCBI |
|  |  |  |  |  |  |  |  |  | JN804114 | NCBI |
|  |  |  |  |  |  |  |  |  | KC920696 | NCBI |
|  |  |  |  |  |  |  |  |  | KF059282 | NCBI |
|  |  |  |  |  |  |  |  |  | JF715010 | NCBI |
|  |  |  |  |  |  |  |  |  | JN804112 | fludb |
|  |  |  |  |  |  |  |  |  | JF715017 | NCBI |
|  |  |  |  |  |  |  |  |  | JN804097 | NCBI |
|  |  |  |  |  |  |  |  |  | JX846585 | NCBI |
|  |  |  |  |  |  |  |  |  | JN804450 | NCBI |
|  |  |  |  |  |  |  |  |  | JF715040 | NCBI |
|  |  |  |  |  |  |  |  |  | KF715244 | NCBI |
|  |  |  |  |  |  |  |  |  | JN804115 | NCBI |
|  |  |  |  |  |  |  |  |  | JN804402 | NCBI |
|  |  |  |  |  |  |  |  |  | JX448768 | fludb |
|  |  |  |  |  |  |  |  |  | JF715039 | NCBI |
|  |  |  |  |  |  |  |  |  | KF059283 | NCBI |
|  |  |  |  |  |  |  |  |  | JF715041 | NCBI |
|  |  |  |  |  |  |  |  |  | JX448758 | NCBI |
|  |  |  |  |  |  |  |  |  | JN804109 | NCBI |
|  |  |  |  |  |  |  |  |  | KM609560 | NCBI |
|  |  |  |  |  |  |  |  |  | JN804098 | NCBI |
|  |  |  |  |  |  |  |  |  | JX448764 | fludb |
|  |  |  |  |  |  |  |  |  | KF746802 | NCBI |
|  |  |  |  |  |  |  |  |  | JN804113 | NCBI |
|  |  |  |  |  |  |  |  |  | KJ419947 | NCBI |
|  |  |  |  |  |  |  |  |  | JF715053 | NCBI |
|  |  |  |  |  |  |  |  |  | JX448759 | NCBI |
|  |  |  |  |  |  |  |  |  | JN804100 | NCBI |
|  |  |  |  |  |  |  |  |  | KF714775 | NCBI |
|  |  |  |  |  |  |  |  |  | KM609561 | NCBI |
|  |  |  |  |  |  |  |  |  | KF259169 | NCBI |
|  |  |  |  |  |  |  |  |  | KF714783 | NCBI |
|  |  |  |  |  |  |  |  |  | KM609562 | NCBI |
|  |  |  |  |  |  |  |  |  | KM609563 | NCBI |
|  |  |  |  |  |  |  |  |  | KM609564 | NCBI |
|  |  |  |  |  |  |  |  |  | KF259165 | NCBI |
|  |  |  |  |  |  |  |  |  | KC817014 | NCBI |
|  |  |  |  |  |  |  |  |  | KF259168 | NCBI |
|  |  |  |  |  |  |  |  |  | KM609565 | NCBI |
|  |  |  |  |  |  |  |  |  | KM609566 | NCBI |
|  |  |  |  |  |  |  |  |  | KF259170 | NCBI |
|  |  |  |  |  |  |  |  |  | KM609567 | NCBI |
|  |  |  |  |  |  |  |  |  | KC879300 | NCBI |
|  |  |  |  |  |  |  |  |  | KF259167 | NCBI |
|  |  |  |  |  |  |  |  |  | KM609568 | NCBI |
|  |  |  |  |  |  |  |  |  | KM609569 | NCBI |
|  |  |  |  |  |  |  |  |  | KC879299 | NCBI |
|  |  |  |  |  |  |  |  |  | KF259189 | fludb |
|  |  |  |  |  |  |  |  |  | KJ419951 | NCBI |
|  |  |  |  |  |  |  |  |  | KF259190 | fludb |
|  |  |  |  |  |  |  |  |  | KF259172 | NCBI |
|  |  |  |  |  |  |  |  |  | KM609570 | NCBI |
|  |  |  |  |  |  |  |  |  | KM609571 | NCBI |
|  |  |  |  |  |  |  |  |  | KM609572 | NCBI |
|  |  |  |  |  |  |  |  |  | KF259171 | NCBI |
|  |  |  |  |  |  |  |  |  | KJ419949 | NCBI |
|  |  |  |  |  |  |  |  |  | KM609573 | NCBI |
|  |  |  |  |  |  |  |  |  | KC951122 | NCBI |
|  |  |  |  |  |  |  |  |  | KM609574 | NCBI |
|  |  |  |  |  |  |  |  |  | JF715029 | NCBI |
|  |  |  |  |  |  |  |  |  | KF259174 | NCBI |
|  |  |  |  |  |  |  |  |  | KF259193 | fludb |
|  |  |  |  |  |  |  |  |  | KC417047 | NCBI |
|  |  |  |  |  |  |  |  |  | CY146660 | NCBI |
|  |  |  |  |  |  |  |  |  | KM609575 | NCBI |
|  |  |  |  |  |  |  |  |  | KC417048 | NCBI |
|  |  |  |  |  |  |  |  |  | JX122435 | NCBI |
|  |  |  |  |  |  |  |  |  | KM609576 | NCBI |
|  |  |  |  |  |  |  |  |  | KC920693 | NCBI |
|  |  |  |  |  |  |  |  |  | KM609577 | NCBI |
|  |  |  |  |  |  |  |  |  | JQ356872 | NCBI |
|  |  |  |  |  |  |  |  |  | KJ419952 | NCBI |
|  |  |  |  |  |  |  |  |  | KM609578 | NCBI |
|  |  |  |  |  |  |  |  |  | KC417046 | NCBI |
|  |  |  |  |  |  |  |  |  | JF715014 | NCBI |
|  |  |  |  |  |  |  |  |  | KM609579 | NCBI |
|  |  |  |  |  |  |  |  |  | KM609580 | NCBI |
|  |  |  |  |  |  |  |  |  | KM609581 | NCBI |
|  |  |  |  |  |  |  |  |  | KC879301 | NCBI |
|  |  |  |  |  |  |  |  |  | KM609582 | NCBI |
|  |  |  |  |  |  |  |  |  | KF259164 | NCBI |
|  |  |  |  |  |  |  |  |  | KF297301 | NCBI |
|  |  |  |  |  |  |  |  |  | KM609583 | NCBI |
|  |  |  |  |  |  |  |  |  | KM609584 | NCBI |
|  |  |  |  |  |  |  |  |  | KM609585 | NCBI |
|  |  |  |  |  |  |  |  |  | KC879298 | NCBI |
|  |  |  |  |  |  |  |  |  | KM609586 | NCBI |
|  |  |  |  |  |  |  |  |  | KF259187 | NCBI |
|  |  |  |  |  |  |  |  |  | KM609587 | NCBI |
|  |  |  |  |  |  |  |  |  | KM609588 | NCBI |
|  |  |  |  |  |  |  |  |  | KM609589 | NCBI |
|  |  |  |  |  |  |  |  |  | KM609590 | NCBI |
|  |  |  |  |  |  |  |  |  | KF259184 | NCBI |
|  |  |  |  |  |  |  |  |  | KM609591 | NCBI |
|  |  |  |  |  |  |  |  |  | KF178670 | NCBI |
|  |  |  |  |  |  |  |  |  | KF500977 | NCBI |
|  |  |  |  |  |  |  |  |  | KF178662 | NCBI |
|  |  |  |  |  |  |  |  |  | KF178694 | NCBI |
|  |  |  |  |  |  |  |  |  | KM609592 | NCBI |
|  |  |  |  |  |  |  |  |  | KF297299 | NCBI |
|  |  |  |  |  |  |  |  |  | KF259182 | fludb |
|  |  |  |  |  |  |  |  |  | KM609593 | NCBI |
|  |  |  |  |  |  |  |  |  | KF259162 | NCBI |
|  |  |  |  |  |  |  |  |  | KF259173 | NCBI |
|  |  |  |  |  |  |  |  |  | KF178678 | NCBI |
|  |  |  |  |  |  |  |  |  | KF297298 | NCBI |
|  |  |  |  |  |  |  |  |  | KF259192 | fludb |
|  |  |  |  |  |  |  |  |  | KJ128362 | NCBI |
|  |  |  |  |  |  |  |  |  | KF259175 | NCBI |
|  |  |  |  |  |  |  |  |  | KF259177 | fludb |
|  |  |  |  |  |  |  |  |  | KF259191 | fludb |
|  |  |  |  |  |  |  |  |  | KF297295 | NCBI |
|  |  |  |  |  |  |  |  |  | KF297300 | NCBI |
|  |  |  |  |  |  |  |  |  | KF259178 | NCBI |
|  |  |  |  |  |  |  |  |  | KF259188 | NCBI |
|  |  |  |  |  |  |  |  |  | KF259176 | fludb |
|  |  |  |  |  |  |  |  |  | KF259180 | fludb |
|  |  |  |  |  |  |  |  |  | KF259166 | NCBI |
|  |  |  |  |  |  |  |  |  | KF178686 | NCBI |
|  |  |  |  |  |  |  |  |  | KF259181 | NCBI |
|  |  |  |  |  |  |  |  |  | KF259185 | NCBI |
|  |  |  |  |  |  |  |  |  | KF259161 | NCBI |
|  |  |  |  |  |  |  |  |  | KF259186 | NCBI |
|  |  |  |  |  |  |  |  |  | KF259160 | NCBI |
|  |  |  |  |  |  |  |  |  | KF259159 | NCBI |
|  |  |  |  |  |  |  |  |  | KF259183 | fludb |
|  |  |  |  |  |  |  |  |  | KF297302 | NCBI |
|  |  |  |  |  |  |  |  |  | KF297296 | NCBI |
|  |  |  |  |  |  |  |  |  | KF259163 | fludb |
|  |  |  |  |  |  |  |  |  | KF297297 | NCBI |
|  |  |  |  |  |  |  |  |  | KF259179 | NCBI |
|  |  |  |  |  |  |  |  |  | KM609594 | NCBI |
|  |  |  |  |  |  |  |  |  | KM609595 | NCBI |
|  |  |  |  |  |  |  |  |  | KM609596 | NCBI |
|  |  |  |  |  |  |  |  |  | KM609597 | NCBI |
|  |  |  |  |  |  |  |  |  | KM609598 | NCBI |
|  |  |  |  |  |  |  |  |  | KM609599 | NCBI |
|  |  |  |  |  |  |  |  |  |  |  |
| NP | |  | NA | |  | M | |  | NS | |
| DQ997453 | NCBI |  | KF971991 | NCBI |  | DQ997445 | NCBI |  | FJ793392 | NCBI |
| JF916714 | NCBI |  | KF971983 | NCBI |  | DQ997425 | NCBI |  | DQ997443 | NCBI |
| EF155164 | NCBI |  | KF972063 | NCBI |  | DQ997457 | NCBI |  | CY024036 | NCBI |
| EF155162 | NCBI |  | CY024034 | NCBI |  | KF972008 | NCBI |  | HQ141884 | NCBI |
| CY024121 | NCBI |  | JX437690 | NCBI |  | KF972016 | NCBI |  | FJ793288 | NCBI |
| EU532038 | NCBI |  | KF972023 | NCBI |  | KF972024 | NCBI |  | CY024020 | NCBI |
| EF155147 | NCBI |  | KF972031 | NCBI |  | AF156462 | NCBI |  | FJ384755 | NCBI |
| EF155126 | NCBI |  | KF972111 | NCBI |  | KF971968 | NCBI |  | DQ997190 | NCBI |
| CY024249 | fludb |  | KF971999 | NCBI |  | KF972032 | NCBI |  | EU365375 | NCBI |
| DQ226139 | NCBI |  | KC162235 | NCBI |  | KC162239 | NCBI |  | AF156476 | NCBI |
| JF916722 | NCBI |  | KF972119 | NCBI |  | KF972000 | NCBI |  | KC162240 | NCBI |
| AB020778 | NCBI |  | KF972055 | NCBI |  | JX437684 | NCBI |  | HQ141885 | NCBI |
| CY024209 | fludb |  | KF971975 | NCBI |  | CY024035 | NCBI |  | DQ997501 | NCBI |
| AF523412 | NCBI |  | KF971951 | NCBI |  | GU903096 | NCBI |  | KF260091 | NCBI |
| CY023713 | NCBI |  | KF972071 | NCBI |  | KF971976 | NCBI |  | EF155231 | NCBI |
| EF155144 | NCBI |  | KF972015 | NCBI |  | KF971992 | NCBI |  | CY055152 | NCBI |
| AF508609 | NCBI |  | AF156395 | NCBI |  | KF972040 | NCBI |  | DQ064471 | NCBI |
| DQ226141 | NCBI |  | CY024018 | NCBI |  | KF972112 | NCBI |  | JQ901699 | fludb |
| EF155148 | NCBI |  | KF971967 | NCBI |  | CY024019 | NCBI |  | JQ901698 | NCBI |
| CY023273 | NCBI |  | KF972079 | fludb |  | KF971984 | NCBI |  | AF222676 | fludb |
| CY023801 | NCBI |  | KF972087 | fludb |  | KF972120 | NCBI |  | EF155240 | NCBI |
| CY024065 | NCBI |  | KF972095 | fludb |  | EU516314 | NCBI |  | JQ901695 | NCBI |
| DQ064458 | NCBI |  | KF971959 | NCBI |  | KF972048 | NCBI |  | CY024564 | NCBI |
| JF795137 | NCBI |  | KF972047 | NCBI |  | KF971960 | NCBI |  | AF222674 | NCBI |
| AY043026 | NCBI |  | KF972103 | NCBI |  | KF971952 | NCBI |  | EF155254 | NCBI |
| CY024081 | fludb |  | KF972039 | NCBI |  | KF259320 | NCBI |  | JQ901696 | fludb |
| CY023361 | NCBI |  | KF972007 | NCBI |  | KF715231 | NCBI |  | KF260135 | NCBI |
| AF156409 | NCBI |  | DQ064417 | NCBI |  | AF222668 | NCBI |  | EF155234 | NCBI |
| EF155166 | NCBI |  | CY024210 | NCBI |  | FJ793375 | NCBI |  | EF155233 | NCBI |
| AF156408 | NCBI |  | AF156393 | NCBI |  | DQ064399 | NCBI |  | AF222675 | fludb |
| DQ064454 | NCBI |  | CY023906 | NCBI |  | AF222669 | NCBI |  | EF155215 | NCBI |
| AY664731 | NCBI |  | DQ064413 | NCBI |  | CY024587 | NCBI |  | CY024556 | NCBI |
| CY023985 | NCBI |  | CY023882 | NCBI |  | FJ793335 | NCBI |  | EF155247 | NCBI |
| CY024089 | NCBI |  | JF916707 | NCBI |  | EU532036 | NCBI |  | AF156477 | NCBI |
| AF523411 | NCBI |  | AF523399 | NCBI |  | DQ064395 | NCBI |  | DQ064472 | fludb |
| AF523410 | NCBI |  | CY024626 | NCBI |  | CY024611 | NCBI |  | EF155244 | NCBI |
| EF155165 | NCBI |  | AF523398 | NCBI |  | KF715239 | NCBI |  | JQ901697 | NCBI |
| EF155139 | NCBI |  | CY023850 | NCBI |  | JQ639789 | NCBI |  | KF188320 | NCBI |
| DQ064455 | NCBI |  | CY024698 | NCBI |  | EU532032 | NCBI |  | EF155230 | NCBI |
| AF523413 | NCBI |  | DQ064420 | NCBI |  | CY023339 | NCBI |  | EF155248 | NCBI |
| CY023889 | NCBI |  | CY024618 | NCBI |  | AY664683 | NCBI |  | AF222672 | NCBI |
| DQ064457 | NCBI |  | AY043024 | NCBI |  | DQ064391 | NCBI |  | EF155266 | NCBI |
| EF155138 | NCBI |  | AF523400 | NCBI |  | FJ793367 | NCBI |  | EF155251 | NCBI |
| CY024225 | NCBI |  | AY043021 | NCBI |  | CY024347 | NCBI |  | EF155200 | NCBI |
| CY024257 | NCBI |  | AF156391 | NCBI |  | AY664694 | NCBI |  | JQ901693 | NCBI |
| CY023092 | NCBI |  | DQ064411 | NCBI |  | AY664689 | NCBI |  | EF155252 | NCBI |
| CY024113 | NCBI |  | CY024682 | NCBI |  | CY075033 | NCBI |  | EF155241 | NCBI |
| CY024377 | NCBI |  | DQ064408 | NCBI |  | FJ793295 | NCBI |  | AJ404735 | NCBI |
| CY024425 | NCBI |  | AF508590 | NCBI |  | CY023179 | NCBI |  | GU053182 | NCBI |
| CY024137 | NCBI |  | CY023898 | NCBI |  | CY023563 | NCBI |  | AJ278649 | NCBI |
| CY024185 | fludb |  | EF155053 | NCBI |  | DQ485211 | NCBI |  | EF155206 | NCBI |
| DQ226140 | NCBI |  | AF156392 | NCBI |  | CY023923 | NCBI |  | EF155201 | NCBI |
| CY024193 | fludb |  | DQ064416 | fludb |  | CY023195 | NCBI |  | EF155271 | NCBI |
| CY024313 | NCBI |  | CY024122 | NCBI |  | FJ793319 | NCBI |  | JQ901700 | NCBI |
| CY024449 | fludb |  | AF523397 | NCBI |  | DQ064387 | NCBI |  | EF155228 | NCBI |
| CY024401 | NCBI |  | EF155079 | NCBI |  | EU086227 | NCBI |  | JQ901692 | NCBI |
| CY024217 | NCBI |  | CY024730 | NCBI |  | CY023155 | NCBI |  | JQ901701 | NCBI |
| CY024233 | NCBI |  | CY023450 | NCBI |  | AF536724 | NCBI |  | CY023100 | NCBI |
| CY023321 | NCBI |  | EF155078 | NCBI |  | CY024651 | NCBI |  | JQ901694 | NCBI |
| CY024305 | fludb |  | CY024734 | NCBI |  | FJ793359 | NCBI |  | EF155250 | NCBI |
| CY024465 | fludb |  | EF155077 | NCBI |  | JF916724 | NCBI |  | EF155216 | NCBI |
| CY023281 | fludb |  | EF155062 | NCBI |  | CY024627 | NCBI |  | EF155269 | fludb |
| CY024321 | NCBI |  | AF523394 | NCBI |  | GQ373121 | NCBI |  | JQ901702 | NCBI |
| CY024001 | NCBI |  | CY023946 | NCBI |  | GQ477301 | NCBI |  | AY966002 | NCBI |
| CY024153 | NCBI |  | CY024714 | NCBI |  | CY023235 | NCBI |  | EF155204 | NCBI |
| EF155170 | NCBI |  | CY024386 | NCBI |  | CY023163 | NCBI |  | AF222673 | NCBI |
| CY024049 | NCBI |  | CY024058 | NCBI |  | DQ981629 | NCBI |  | EF155218 | NCBI |
| CY024273 | fludb |  | EF155066 | NCBI |  | AF222667 | NCBI |  | EF155229 | NCBI |
| CY024369 | NCBI |  | EF155080 | NCBI |  | DQ226103 | NCBI |  | EF155253 | NCBI |
| CY024281 | NCBI |  | CY023122 | NCBI |  | DQ226105 | NCBI |  | HQ117890 | fludb |
| CY023385 | NCBI |  | CY023866 | NCBI |  | DQ981621 | NCBI |  | EF155267 | NCBI |
| CY024161 | NCBI |  | CY023538 | NCBI |  | CY023699 | NCBI |  | EF155222 | NCBI |
| EF155176 | NCBI |  | CY023186 | NCBI |  | CY023979 | NCBI |  | AY631868 | NCBI |
| CY023289 | NCBI |  | CY023362 | NCBI |  | EF155007 | NCBI |  | AF156480 | NCBI |
| EF155159 | NCBI |  | CY023626 | NCBI |  | CY024691 | NCBI |  | AF508716 | NCBI |
| CY023425 | NCBI |  | CY023106 | NCBI |  | CY023595 | NCBI |  | AF156478 | NCBI |
| CY024505 | NCBI |  | EF155065 | NCBI |  | KF746846 | NCBI |  | AY259219 | NCBI |
| CY024497 | NCBI |  | CY023858 | NCBI |  | CY005507 | NCBI |  | AF508721 | NCBI |
| EF155172 | NCBI |  | CY023922 | NCBI |  | AF508698 | NCBI |  | AY912496 | NCBI |
| CY024265 | NCBI |  | CY023938 | NCBI |  | JF795113 | NCBI |  | DQ064468 | NCBI |
| CY024289 | fludb |  | CY024666 | NCBI |  | CY023467 | NCBI |  | DQ997484 | NCBI |
| AF508607 | NCBI |  | CY024658 | NCBI |  | DQ226099 | NCBI |  | CY023868 | fludb |
| EU516316 | NCBI |  | CY023874 | fludb |  | EF155037 | NCBI |  | AF222679 | NCBI |
| EU502897 | NCBI |  | CY023994 | fludb |  | CY077087 | NCBI |  | JQ904464 | NCBI |
| AY664732 | NCBI |  | CY024026 | NCBI |  | DQ064392 | NCBI |  | CY023756 | NCBI |
| CY024641 | NCBI |  | CY024106 | fludb |  | AY664679 | NCBI |  | CY024692 | NCBI |
| JQ639779 | NCBI |  | CY023250 | fludb |  | CY023971 | NCBI |  | EU532051 | NCBI |
| DQ485209 | NCBI |  | CY023226 | NCBI |  | AF156461 | NCBI |  | AF508719 | NCBI |
| DQ064441 | NCBI |  | CY023954 | NCBI |  | CY023203 | NCBI |  | CY023876 | NCBI |
| AF156402 | NCBI |  | CY023258 | NCBI |  | CY024435 | NCBI |  | CY024588 | NCBI |
| DQ064450 | NCBI |  | CY024098 | NCBI |  | DQ981613 | NCBI |  | AF523507 | NCBI |
| KF746811 | NCBI |  | CY024010 | fludb |  | CY023731 | NCBI |  | CY024708 | NCBI |
| AF186272 | NCBI |  | CY024170 | fludb |  | FJ793311 | NCBI |  | AF156474 | NCBI |
| AF222620 | NCBI |  | EF155099 | NCBI |  | KF259317 | NCBI |  | AF222677 | NCBI |
| AF156403 | NCBI |  | CY023234 | NCBI |  | FJ793351 | NCBI |  | EU081868 | NCBI |
| AF156404 | NCBI |  | CY024242 | NCBI |  | CY023579 | NCBI |  | KF746743 | NCBI |
| AF508612 | NCBI |  | CY023970 | NCBI |  | DQ064403 | NCBI |  | AY259226 | NCBI |
| DQ064439 | NCBI |  | CY023162 | NCBI |  | DQ064388 | NCBI |  | CY023932 | NCBI |
| CY024633 | NCBI |  | EF155100 | NCBI |  | EU086267 | NCBI |  | CY023940 | NCBI |
| JQ904462 | NCBI |  | CY024202 | fludb |  | JF916716 | NCBI |  | DQ997470 | NCBI |
| DQ064460 | NCBI |  | CY024130 | NCBI |  | EU532031 | NCBI |  | AY180593 | fludb |
| FJ793437 | fludb |  | CY023146 | NCBI |  | DQ064397 | NCBI |  | EU532053 | NCBI |
| DQ226144 | NCBI |  | CY023130 | NCBI |  | AY664681 | NCBI |  | AF523506 | NCBI |
| AF508614 | NCBI |  | CY023114 | NCBI |  | CY024635 | NCBI |  | CY023188 | NCBI |
| AF508617 | NCBI |  | CY023930 | NCBI |  | AF156460 | NCBI |  | CY006022 | fludb |
| HQ117887 | NCBI |  | EF155096 | NCBI |  | JF795119 | NCBI |  | AF156472 | NCBI |
| FJ793405 | NCBI |  | CY024178 | fludb |  | JQ639781 | NCBI |  | CY023140 | NCBI |
| DQ064445 | fludb |  | CY023242 | NCBI |  | CY023331 | NCBI |  | CY024596 | NCBI |
| DQ064437 | NCBI |  | CY023178 | NCBI |  | KF746862 | NCBI |  | CY024628 | NCBI |
| DQ064440 | NCBI |  | EF155093 | NCBI |  | AF536725 | NCBI |  | CY023132 | NCBI |
| EU502905 | NCBI |  | CY023266 | NCBI |  | DQ064385 | NCBI |  | AF222681 | NCBI |
| AF222814 | NCBI |  | EF155090 | NCBI |  | AF222822 | NCBI |  | CY024620 | fludb |
| DQ064436 | NCBI |  | EF155071 | NCBI |  | CY024643 | NCBI |  | EU532049 | NCBI |
| AY496851 | NCBI |  | CY024146 | NCBI |  | AF523486 | NCBI |  | KF746766 | NCBI |
| FJ793309 | NCBI |  | EF155089 | NCBI |  | DQ997498 | NCBI |  | AF523508 | NCBI |
| DQ064452 | NCBI |  | AF523392 | NCBI |  | EU532034 | NCBI |  | AF508720 | NCBI |
| JQ639787 | NCBI |  | CY023714 | NCBI |  | AF523493 | NCBI |  | DQ981614 | NCBI |
| EU532040 | NCBI |  | CY023802 | NCBI |  | CY024675 | NCBI |  | CY023332 | fludb |
| AF400781 | NCBI |  | CY024674 | NCBI |  | CY023259 | NCBI |  | HQ141876 | NCBI |
| AY180583 | NCBI |  | CY024050 | NCBI |  | CY023307 | NCBI |  | DQ064470 | NCBI |
| DQ064435 | NCBI |  | EF155081 | NCBI |  | KF259321 | NCBI |  | JQ639782 | fludb |
| DQ064444 | NCBI |  | CY024650 | NCBI |  | GU121385 | NCBI |  | AF156473 | NCBI |
| DQ064453 | NCBI |  | AF523391 | NCBI |  | HQ425337 | NCBI |  | EU532055 | NCBI |
| AF508611 | NCBI |  | CY023890 | fludb |  | KF746838 | NCBI |  | DQ064486 | NCBI |
| FJ793381 | NCBI |  | EF155086 | NCBI |  | CY024243 | NCBI |  | DQ997463 | NCBI |
| DQ064443 | NCBI |  | CY024722 | NCBI |  | DQ226101 | NCBI |  | CY023308 | NCBI |
| AF222815 | fludb |  | CY023274 | NCBI |  | HM590772 | NCBI |  | CY077088 | NCBI |
| CY005509 | NCBI |  | EF155075 | NCBI |  | DQ485219 | NCBI |  | KF746758 | NCBI |
| AF508610 | NCBI |  | CY023218 | NCBI |  | FJ793407 | NCBI |  | CY023916 | NCBI |
| AF156405 | NCBI |  | AF523393 | NCBI |  | FJ793415 | NCBI |  | CY023900 | NCBI |
| DQ064438 | NCBI |  | EF155074 | NCBI |  | FJ793343 | NCBI |  | CY023116 | NCBI |
| EF070735 | NCBI |  | CY023986 | NCBI |  | CY023267 | NCBI |  | CY006024 | NCBI |
| AY268949 | NCBI |  | CY023170 | NCBI |  | DQ981541 | NCBI |  | CY023460 | NCBI |
| CY075039 | NCBI |  | CY024090 | NCBI |  | FJ793447 | NCBI |  | EU753302 | NCBI |
| AY664722 | NCBI |  | CY024074 | NCBI |  | DQ064386 | NCBI |  | CY023924 | NCBI |
| DQ064456 | NCBI |  | CY024690 | NCBI |  | EU532056 | NCBI |  | DQ064462 | NCBI |
| DQ064461 | NCBI |  | AF523395 | NCBI |  | DQ064406 | NCBI |  | CY024580 | NCBI |
| AF222621 | NCBI |  | CY024706 | NCBI |  | AF536719 | NCBI |  | DQ064476 | NCBI |
| EU532044 | NCBI |  | CY023914 | NCBI |  | DQ064407 | NCBI |  | AF156475 | NCBI |
| KF746860 | NCBI |  | CY024298 | NCBI |  | EF155041 | NCBI |  | EF070736 | NCBI |
| EU532043 | NCBI |  | CY024218 | NCBI |  | KF746854 | NCBI |  | EU086241 | NCBI |
| FJ793429 | NCBI |  | CY024282 | NCBI |  | JF795139 | NCBI |  | DQ465404 | NCBI |
| CY075047 | NCBI |  | CY024322 | NCBI |  | DQ064390 | NCBI |  | EU250006 | NCBI |
| DQ064451 | NCBI |  | CY024002 | NCBI |  | DQ997512 | NCBI |  | DQ064488 | NCBI |
| KF746757 | NCBI |  | DQ226130 | NCBI |  | AF508697 | NCBI |  | AY259220 | NCBI |
| AF186271 | NCBI |  | CY024306 | NCBI |  | CY024723 | NCBI |  | EF155207 | NCBI |
| CY077085 | NCBI |  | CY024314 | NCBI |  | DQ064389 | NCBI |  | DQ485228 | NCBI |
| KF746828 | NCBI |  | CY024154 | NCBI |  | CY023187 | NCBI |  | AY180594 | fludb |
| CY005515 | fludb |  | CY023282 | NCBI |  | AF523491 | NCBI |  | KF746856 | NCBI |
| KF746763 | NCBI |  | CY024194 | NCBI |  | DQ997472 | NCBI |  | CY023340 | NCBI |
| KF746836 | NCBI |  | CY024066 | NCBI |  | CY023227 | NCBI |  | CY023108 | NCBI |
| AY664725 | NCBI |  | CY023322 | NCBI |  | DQ064398 | NCBI |  | EU753334 | NCBI |
| DQ465401 | fludb |  | CY024186 | NCBI |  | KF259316 | NCBI |  | CY023452 | NCBI |
| DQ064446 | NCBI |  | CY024290 | fludb |  | EF154989 | NCBI |  | FJ793416 | NCBI |
| DQ226143 | NCBI |  | CY023290 | NCBI |  | CY023107 | NCBI |  | GQ477303 | fludb |
| CY075055 | NCBI |  | CY024162 | NCBI |  | AY664692 | NCBI |  | CY023948 | NCBI |
| AF222619 | NCBI |  | CY024138 | NCBI |  | EU086286 | NCBI |  | AF508717 | NCBI |
| DQ064449 | NCBI |  | CY024266 | NCBI |  | CY024395 | NCBI |  | FJ793320 | NCBI |
| AF508608 | NCBI |  | CY024258 | fludb |  | EF155020 | NCBI |  | CY075050 | NCBI |
| AY788915 | NCBI |  | CY024082 | NCBI |  | DQ997473 | NCBI |  | DQ064473 | NCBI |
| KF746877 | NCBI |  | CY024234 | NCBI |  | CY023139 | NCBI |  | AY180592 | NCBI |
| FJ793413 | NCBI |  | EF155097 | NCBI |  | AF536727 | NCBI |  | CY024652 | fludb |
| CY075031 | NCBI |  | CY024250 | NCBI |  | CY023211 | NCBI |  | CY023860 | NCBI |
| EU532037 | NCBI |  | EF155103 | NCBI |  | CY023915 | NCBI |  | CY024476 | NCBI |
| CY024697 | NCBI |  | CY024114 | NCBI |  | AF156466 | NCBI |  | CY075042 | NCBI |
| DQ064447 | NCBI |  | CY024226 | NCBI |  | EU532030 | NCBI |  | DQ064483 | NCBI |
| JF795130 | NCBI |  | DQ226129 | NCBI |  | CY023131 | NCBI |  | CY024676 | NCBI |
| CY024537 | fludb |  | CY024538 | NCBI |  | CY023299 | NCBI |  | FJ793368 | NCBI |
| JQ901667 | fludb |  | KF188336 | NCBI |  | KF259322 | NCBI |  | FJ793400 | NCBI |
| CY023809 | NCBI |  | CY023426 | NCBI |  | CY023899 | NCBI |  | EU532047 | NCBI |
| EF155197 | fludb |  | CY024546 | NCBI |  | AF536728 | NCBI |  | CY075058 | NCBI |
| KF259891 | NCBI |  | KF259535 | NCBI |  | DQ064384 | NCBI |  | FJ793432 | NCBI |
| JQ901661 | fludb |  | CY024378 | fludb |  | AF536722 | NCBI |  | DQ997479 | NCBI |
| AF222614 | NCBI |  | CY023386 | NCBI |  | KF746790 | NCBI |  | EU753318 | fludb |
| CY024529 | NCBI |  | CY024466 | NCBI |  | EF154984 | NCBI |  | EF155226 | NCBI |
| AF255742 | NCBI |  | KF259483 | NCBI |  | EU414522 | NCBI |  | DQ485220 | NCBI |
| JQ901663 | NCBI |  | CY024402 | NCBI |  | CY024131 | NCBI |  | CY023908 | fludb |
| AF222616 | NCBI |  | CY024274 | NCBI |  | CY075041 | NCBI |  | EF155203 | NCBI |
| JQ901665 | fludb |  | EF155124 | NCBI |  | AF523489 | NCBI |  | AF523510 | NCBI |
| GU053181 | NCBI |  | CY055150 | NCBI |  | CY024683 | NCBI |  | AF400790 | NCBI |
| AF156407 | NCBI |  | CY024506 | NCBI |  | CY005501 | NCBI |  | CY023884 | NCBI |
| CY024545 | fludb |  | KF188318 | NCBI |  | CY024715 | NCBI |  | KF746831 | NCBI |
| AF222618 | NCBI |  | CY024370 | NCBI |  | EU086305 | NCBI |  | CY024732 | NCBI |
| JQ901666 | NCBI |  | KF259534 | NCBI |  | CY024355 | NCBI |  | CY075034 | NCBI |
| JQ901660 | fludb |  | EF155092 | NCBI |  | EU753349 | NCBI |  | CY023316 | NCBI |
| JQ901659 | fludb |  | KF259532 | NCBI |  | CY023315 | NCBI |  | CY023852 | NCBI |
| EF155128 | NCBI |  | CY024426 | NCBI |  | CY023355 | NCBI |  | AY180595 | fludb |
| EF155195 | NCBI |  | EF155122 | NCBI |  | DQ064393 | NCBI |  | DQ064463 | NCBI |
| EF155194 | fludb |  | AY664712 | NCBI |  | CY024731 | NCBI |  | CY006020 | NCBI |
| KF259847 | NCBI |  | DQ226128 | NCBI |  | AF508695 | NCBI |  | EF155202 | NCBI |
| EF155198 | fludb |  | CY024498 | NCBI |  | CY023115 | NCBI |  | CY024604 | NCBI |
| AF222617 | NCBI |  | CY024450 | fludb |  | CY024667 | NCBI |  | AY259224 | NCBI |
| AJ289872 | NCBI |  | CY023810 | NCBI |  | CY023443 | NCBI |  | EU532054 | NCBI |
| CY055149 | NCBI |  | CY024530 | NCBI |  | AY664695 | NCBI |  | FJ793384 | NCBI |
| JQ901668 | fludb |  | EF155091 | NCBI |  | KF746830 | NCBI |  | CY024724 | NCBI |
| KF188317 | NCBI |  | CY024458 | fludb |  | CY024571 | NCBI |  | CY024716 | fludb |
| CY024553 | NCBI |  | JQ901675 | fludb |  | DQ997436 | NCBI |  | CY023124 | NCBI |
| AF222615 | NCBI |  | KF259533 | NCBI |  | KF259308 | NCBI |  | EU753286 | NCBI |
| JQ901662 | fludb |  | DQ981572 | NCBI |  | DQ226104 | NCBI |  | CY024612 | fludb |
| EF155193 | NCBI |  | JQ901671 | fludb |  | EF154988 | NCBI |  | CY023980 | NCBI |
| JQ901669 | NCBI |  | KF259538 | fludb |  | AY664688 | NCBI |  | CY024684 | NCBI |
| JQ901664 | fludb |  | DQ981548 | NCBI |  | EU086320 | NCBI |  | CY024148 | NCBI |
| EF155196 | NCBI |  | JQ901670 | fludb |  | AF536721 | NCBI |  | AF523509 | NCBI |
| DQ485217 | NCBI |  | EF626674 | NCBI |  | KF746870 | NCBI |  | EU532048 | NCBI |
| EU081867 | NCBI |  | DQ981540 | NCBI |  | DQ064405 | NCBI |  | DQ064482 | NCBI |
| EF155158 | NCBI |  | JQ901678 | NCBI |  | KF746765 | NCBI |  | AF508725 | NCBI |
| EF155160 | fludb |  | JQ901679 | fludb |  | DQ997194 | NCBI |  | JF916717 | NCBI |
| CY023849 | NCBI |  | JQ901676 | fludb |  | CY024339 | NCBI |  | JF795140 | NCBI |
| EF155141 | fludb |  | DQ981588 | NCBI |  | CY023219 | NCBI |  | AY259216 | NCBI |
| EF155142 | NCBI |  | EF626675 | NCBI |  | EF154991 | NCBI |  | DQ064484 | NCBI |
| CY024561 | NCBI |  | KF259537 | NCBI |  | AF222823 | NCBI |  | DQ064485 | fludb |
| EF155168 | fludb |  | JQ901672 | fludb |  | CY023947 | NCBI |  | AY259221 | fludb |
| EF155167 | NCBI |  | DQ064426 | fludb |  | EU502903 | NCBI |  | AF508718 | fludb |
| EF155127 | NCBI |  | AY451388 | NCBI |  | CY023451 | NCBI |  | DQ997508 | NCBI |
| DQ485225 | NCBI |  | DQ981556 | NCBI |  | CY023603 | NCBI |  | HQ425338 | NCBI |
| EF155137 | NCBI |  | JQ901674 | NCBI |  | CY023347 | NCBI |  | JF916725 | NCBI |
| EF155157 | NCBI |  | DQ064410 | NCBI |  | CY023963 | NCBI |  | AY664750 | NCBI |
| CY023881 | NCBI |  | JQ901680 | NCBI |  | AF222670 | NCBI |  | EF155259 | NCBI |
| EF155171 | NCBI |  | JQ901673 | fludb |  | KF259311 | NCBI |  | CY024356 | fludb |
| CY023097 | fludb |  | DQ981564 | NCBI |  | DQ226100 | NCBI |  | AF222680 | NCBI |
| EF155173 | NCBI |  | JQ901677 | fludb |  | GQ477300 | NCBI |  | CY023292 | NCBI |
| AF156406 | NCBI |  | DQ981596 | NCBI |  | CY023771 | NCBI |  | CY023300 | NCBI |
| EF155175* | fludb |  | EU502894 | NCBI |  | EU753277 | NCBI |  | CY024228 | NCBI |
| EF155177 | NCBI |  | EU502902 | NCBI |  | EU532029 | NCBI |  | KF260107 | NCBI |
| EF155143 | NCBI |  | CY023202 | NCBI |  | AF536726 | NCBI |  | CY024468 | NCBI |
| EF155140 | NCBI |  | CY024522 | NCBI |  | DQ997488 | NCBI |  | KF260110 | NCBI |
| EF155156 | NCBI |  | CY023962 | NCBI |  | DQ981597 | NCBI |  | CY023572 | NCBI |
| EF155132 | NCBI |  | CY023570 | NCBI |  | AF156459 | NCBI |  | CY023564 | fludb |
| CY023865 | fludb |  | CY023298 | NCBI |  | FJ793439 | NCBI |  | CY023540 | fludb |
| EF155149 | NCBI |  | CY023194 | NCBI |  | EU516306 | NCBI |  | CY024508 | NCBI |
| EF155181 | fludb |  | CY023578 | NCBI |  | CY024059 | NCBI |  | EU835744 | NCBI |
| EF155155 | NCBI |  | DQ226133 | NCBI |  | KF746813 | NCBI |  | CY023636 | fludb |
| EF155133 | NCBI |  | AY664703 | NCBI |  | DQ107513 | NCBI |  | KF260120 | NCBI |
| CY023897 | NCBI |  | CY024634 | NCBI |  | AF508704 | NCBI |  | KF260076 | NCBI |
| EF155174 | NCBI |  | CY023138 | NCBI |  | CY075057 | NCBI |  | AF400791 | NCBI |
| EF155136 | NCBI |  | DQ485210 | NCBI |  | FJ384754 | NCBI |  | CY024524 | fludb |
| EF155145 | NCBI |  | DQ226132 | NCBI |  | KF259313 | NCBI |  | KF260112 | fludb |
| CY023905 | NCBI |  | DQ064423 | NCBI |  | CY023123 | NCBI |  | EU835743 | NCBI |
| EF155180 | NCBI |  | DQ226131 | NCBI |  | CY023883 | NCBI |  | KF260079 | NCBI |
| EF155178 | fludb |  | CY023586 | NCBI |  | FJ793431 | NCBI |  | CY024412 | NCBI |
| CY023873 | NCBI |  | CY023482 | NCBI |  | CY023955 | NCBI |  | CY023436 | NCBI |
| EF155131 | NCBI |  | DQ064425 | NCBI |  | AF536720 | NCBI |  | CY024452 | NCBI |
| EF155146 | NCBI |  | DQ485226 | NCBI |  | DQ064383 | NCBI |  | CY023428 | NCBI |
| EF155179 | NCBI |  | EU086288 | NCBI |  | EF155023 | NCBI |  | EF155257 | NCBI |
| CY023857 | NCBI |  | DQ485218 | NCBI |  | AF523492 | NCBI |  | CY024172 | NCBI |
| EF155161 | NCBI |  | AY664706 | NCBI |  | CY005519 | NCBI |  | KF260071 | NCBI |
| KF972030 | NCBI |  | EU081866 | NCBI |  | AY664696 | NCBI |  | AF222678 | NCBI |
| KF971990 | NCBI |  | JQ639788 | NCBI |  | EU753317 | NCBI |  | KF260097 | NCBI |
| KF971974 | NCBI |  | EU086239 | NCBI |  | CY024619 | NCBI |  | EF155263 | NCBI |
| KF971950 | NCBI |  | AY664708 | NCBI |  | AF400778 | NCBI |  | CY023268 | NCBI |
| CY024017 | NCBI |  | DQ226134 | NCBI |  | KF259318 | NCBI |  | CY024212 | NCBI |
| KF972118 | NCBI |  | AY664713 | NCBI |  | DQ064394 | NCBI |  | CY024548 | fludb |
| KF972086 | NCBI |  | CY024642 | NCBI |  | EU835747 | NCBI |  | CY023988 | fludb |
| KF972062 | NCBI |  | CY023594 | NCBI |  | DQ107509 | NCBI |  | CY023660 | fludb |
| KF972006 | NCBI |  | CY024514 | NCBI |  | CY024475 | NCBI |  | CY024404 | NCBI |
| JX437689 | NCBI |  | DQ064415 | NCBI |  | DQ226102 | NCBI |  | CY024396 | fludb |
| KF971966 | NCBI |  | AF523396 | NCBI |  | EF070737 | NCBI |  | GQ373142 | NCBI |
| KF972110 | NCBI |  | CY023474 | NCBI |  | CY024659 | NCBI |  | DQ064475 | NCBI |
| KF972078 | NCBI |  | EF155110 | NCBI |  | CY023531 | NCBI |  | CY024348 | NCBI |
| KC162241 | NCBI |  | CY024410 | fludb |  | AY664697 | NCBI |  | CY024700 | NCBI |
| KF972014 | NCBI |  | CY023466 | NCBI |  | EU753309 | NCBI |  | CY023972 | NCBI |
| KF971982 | NCBI |  | EF155113 | fludb |  | EU502895 | NCBI |  | CY024180 | fludb |
| KF972094 | NCBI |  | CY024338 | NCBI |  | AF508701 | NCBI |  | CY023260 | NCBI |
| KF972102 | NCBI |  | CY023370 | NCBI |  | EF155036 | NCBI |  | CY023612 | fludb |
| CY024033 | NCBI |  | CY023378 | NCBI |  | AF523488 | NCBI |  | KF260131 | fludb |
| KF972046 | NCBI |  | CY024442 | fludb |  | KF746871 | NCBI |  | KF260077 | NCBI |
| KF972070 | NCBI |  | CY023610 | NCBI |  | KF259312 | NCBI |  | CY024644 | NCBI |
| KF971998 | NCBI |  | CY023394 | NCBI |  | KF259309 | NCBI |  | KF260114 | NCBI |
| KF971958 | NCBI |  | EF155109 | NCBI |  | AY664680 | NCBI |  | EF155255 | NCBI |
| KF972022 | NCBI |  | EF155116 | NCBI |  | KF746798 | NCBI |  | CY023596 | fludb |
| KF972054 | NCBI |  | EF155112 | NCBI |  | FJ793303 | NCBI |  | JN869520 | NCBI |
| KF972038 | NCBI |  | CY023346 | NCBI |  | DQ064381 | NCBI |  | CY024004 | NCBI |
| JF795111 | NCBI |  | EF155119 | NCBI |  | AF400779 | NCBI |  | GQ373149 | NCBI |
| CY024617 | NCBI |  | CY023410 | NCBI |  | FJ793383 | NCBI |  | CY024068 | NCBI |
| CY023977 | fludb |  | CY024482 | NCBI |  | CY023435 | NCBI |  | CY023996 | NCBI |
| CY024145 | NCBI |  | CY023546 | NCBI |  | EU086285 | NCBI |  | KF260074 | NCBI |
| EF155134 | NCBI |  | EF155115 | fludb |  | CY023147 | NCBI |  | GQ373147* | fludb |
| CY023233 | NCBI |  | EF155111 | NCBI |  | AF156458 | NCBI |  | EF155258 | NCBI |
| CY005503 | NCBI |  | CY023402 | NCBI |  | CY023755 | NCBI |  | KF260083 | NCBI |
| CY024601 | NCBI |  | CY023562 | NCBI |  | FJ793399 | NCBI |  | EF155236 | NCBI |
| CY023105 | NCBI |  | EF155117 | NCBI |  | CY023851 | NCBI |  | KF260100 | fludb |
| CY024057 | NCBI |  | CY023354 | NCBI |  | AF156464 | NCBI |  | AY664753 | NCBI |
| AF523417 | NCBI |  | CY024362 | NCBI |  | DQ997420 | NCBI |  | CY024164 | NCBI |
| AF523419 | NCBI |  | CY024330 | NCBI |  | CY023459 | NCBI |  | EF155199 | NCBI |
| CY024569 | NCBI |  | CY023442 | NCBI |  | EU753285 | NCBI |  | CY024500 | NCBI |
| EF155129 | NCBI |  | CY024490 | NCBI |  | AY253755 | NCBI |  | CY024668 | NCBI |
| CY024585 | NCBI |  | CY024434 | NCBI |  | KF259314 | NCBI |  | JF916709 | NCBI |
| CY024577 | NCBI |  | CY023434 | NCBI |  | EU532035 | NCBI |  | CY023388 | NCBI |
| CY005521 | NCBI |  | CY023602 | NCBI |  | CY024595 | NCBI |  | CY023548 | NCBI |
| CY024665 | NCBI |  | EF155114 | NCBI |  | CY024699 | NCBI |  | CY023516 | NCBI |
| CY023329 | NCBI |  | CY023554 | NCBI |  | KF259310 | NCBI |  | AF523515 | NCBI |
| GQ477297 | NCBI |  | EF155118 | NCBI |  | EU753293 | NCBI |  | KF260099 | NCBI |
| CY023169 | NCBI |  | CY023418 | NCBI |  | AY664684 | NCBI |  | AY664741 | NCBI |
| CY023129 | NCBI |  | CY024346 | NCBI |  | DQ064382 | NCBI |  | EF155261 | NCBI |
| KF188283 | NCBI |  | CY024394 | fludb |  | DQ997456 | NCBI |  | CY023508 | NCBI |
| CY024609 | NCBI |  | KF259512 | NCBI |  | CY023587 | NCBI |  | DQ064474 | NCBI |
| AY180562 | NCBI |  | KF746779 | NCBI |  | DQ465403 | NCBI |  | CY024204 | fludb |
| CY024673 | NCBI |  | KF259463 | NCBI |  | JF795131 | NCBI |  | CY023804 | NCBI |
| CY023313 | fludb |  | KF259464 | NCBI |  | DQ226098 | NCBI |  | KF260111 | NCBI |
| CY024593 | NCBI |  | HQ398342 | NCBI |  | KF259339 | NCBI |  | JQ228394 | NCBI |
| CY023305 | NCBI |  | KF259466 | NCBI |  | DQ997489 | NCBI |  | EF155209 | NCBI |
| EF155130 | NCBI |  | EU935063 | NCBI |  | CY023411 | NCBI |  | AY664754 | NCBI |
| CY024625 | NCBI |  | KF259500 | NCBI |  | EU086304 | NCBI |  | CY024140 | NCBI |
| EF155135 | NCBI |  | KF746869 | NCBI |  | JQ904463 | NCBI |  | CY024292 | NCBI |
| CY023969 | NCBI |  | KF259477 | NCBI |  | AF523490 | NCBI |  | CY024196 | NCBI |
| CY024681 | NCBI |  | KF259465 | NCBI |  | EU753341 | NCBI |  | CY023236 | NCBI |
| AF523416 | NCBI |  | CY024354 | fludb |  | CY023171 | NCBI |  | CY024660 | NCBI |
| EU086240 | NCBI |  | KF188290 | NCBI |  | EF612743 | NCBI |  | JQ639790 | NCBI |
| CY005527 | NCBI |  | KF259473 | NCBI |  | CY075049 | NCBI |  | KF260108 | NCBI |
| CY023137 | NCBI |  | KF259491 | NCBI |  | FJ793287 | NCBI |  | KF260115 | NCBI |
| AF523420 | NCBI |  | EU216096 | NCBI |  | CY023251 | NCBI |  | CY024052 | NCBI |
| CY024473 | NCBI |  | EU935064 | NCBI |  | CY024579 | NCBI |  | AY664744 | NCBI |
| CY024657 | NCBI |  | KF259511 | NCBI |  | DQ064396 | NCBI |  | CY023476 | NCBI |
| CY024097 | NCBI |  | KF259484 | NCBI |  | EU081865 | NCBI |  | KF260101 | NCBI |
| CY023953 | NCBI |  | FJ492965 | NCBI |  | DQ064402 | NCBI |  | KF746872 | NCBI |
| CY024721 | NCBI |  | EU216105 | NCBI |  | FJ793327 | NCBI |  | EU835745 | NCBI |
| CY023937 | NCBI |  | EU216095 | NCBI |  | EU753333 | NCBI |  | GQ373151 | NCBI |
| CY023945 | fludb |  | KF259476 | NCBI |  | FJ793423 | NCBI |  | CY024300 | NCBI |
| CY023185 | NCBI |  | JN683652 | NCBI |  | CY023859 | NCBI |  | CY023812 | NCBI |
| CY023929 | NCBI |  | KF259475 | NCBI |  | FJ793391 | NCBI |  | CY024084 | NCBI |
| CY023153 | fludb |  | CY023530 | NCBI |  | EU086235 | NCBI |  | CY024372 | NCBI |
| CY024489 | fludb |  | KF259469 | NCBI |  | AY664687 | NCBI |  | DQ981566 | NCBI |
| CY023161 | NCBI |  | CY024418 | fludb |  | CY024443 | NCBI |  | CY023796 | NCBI |
| CY024385 | NCBI |  | HQ398345 | NCBI |  | KF259356 | NCBI |  | AY043027 | NCBI |
| CY023121 | NCBI |  | EU216098 | NCBI |  | AY664693 | NCBI |  | EF155205 | NCBI |
| CY024689 | fludb |  | HQ398339 | NCBI |  | CY023787 | NCBI |  | KF260095 | fludb |
| CY024649 | NCBI |  | KF259472 | NCBI |  | GQ373129 | NCBI |  | EF155220 | NCBI |
| CY023913 | NCBI |  | HQ398340 | NCBI |  | GQ202045 | NCBI |  | CY023324 | NCBI |
| CY024729 | NCBI |  | KF259467 | NCBI |  | KF259334 | NCBI |  | EF155213 | fludb |
| EF155163 | NCBI |  | KF259499 | NCBI |  | KF259384 | NCBI |  | KF260080 | NCBI |
| CY023145 | NCBI |  | KF259485 | NCBI |  | CY024371 | NCBI |  | CY023372 | NCBI |
| CY023921 | NCBI |  | EU935062 | NCBI |  | KF259379 | NCBI |  | CY023676 | NCBI |
| CY023201 | NCBI |  | KF259494 | NCBI |  | KF259371 | NCBI |  | KF260084 | NCBI |
| CY024713 | fludb |  | CY023618 | NCBI |  | KC821177 | NCBI |  | KF260106 | NCBI |
| CY023177 | NCBI |  | CY023682 | NCBI |  | KC821179 | NCBI |  | CY023204 | NCBI |
| EF155153 | NCBI |  | CY023650 | NCBI |  | KF259319 | NCBI |  | CY023380 | NCBI |
| CY023417 | NCBI |  | KF259468 | NCBI |  | EF155017 | NCBI |  | DQ226120 | NCBI |
| CY024073 | NCBI |  | CY023674 | NCBI |  | KC821180 | NCBI |  | KF260102 | NCBI |
| CY023113 | fludb |  | CY023506 | NCBI |  | KC767262 | NCBI |  | GQ373145 | NCBI |
| CY024705 | NCBI |  | KF259523 | fludb |  | KF746780 | NCBI |  | CY024268 | fludb |
| CY023337 | NCBI |  | JN869518 | NCBI |  | KC821181 | NCBI |  | CY024340 | NCBI |
| CY023449 | NCBI |  | JN627142 | NCBI |  | KC821178 | NCBI |  | KF260127 | NCBI |
| CY023457 | NCBI |  | KF259497 | NCBI |  | KF178698 | NCBI |  | KF260109 | NCBI |
| CY023409 | NCBI |  | KF259519 | NCBI |  | CY024547 | NCBI |  | CY023524 | NCBI |
| AF523418 | NCBI |  | KF259559 | NCBI |  | CY023779 | NCBI |  | EF155217 | NCBI |
| CY024481 | NCBI |  | CY023634 | NCBI |  | GQ373135 | NCBI |  | CY024108 | fludb |
| DQ997430 | NCBI |  | CY024042 | NCBI |  | CY024091 | NCBI |  | EF155239 | NCBI |
| DQ997500 | NCBI |  | KF188392 | NCBI |  | KF259364 | NCBI |  | CY023556 | fludb |
| DQ997442 | NCBI |  | JQ228391 | NCBI |  | KC821182 | NCBI |  | KF260096 | fludb |
| AY253753 | NCBI |  | CY055158 | NCBI |  | EF155003 | NCBI |  | KF260085 | NCBI |
| EU532041 | NCBI |  | CY023490 | NCBI |  | CY023651 | NCBI |  | KF260125 | NCBI |
| KF746784 | NCBI |  | KF259502 | NCBI |  | CY023283 | NCBI |  | CY023604 | NCBI |
| AF523414 | NCBI |  | CY023642 | NCBI |  | CY023739 | NCBI |  | DQ064478 | NCBI |
| DQ997483 | NCBI |  | KF259495 | NCBI |  | KC821183 | NCBI |  | EF155249 | NCBI |
| DQ064448 | NCBI |  | JN222382 | NCBI |  | EF154982 | NCBI |  | CY023740 | NCBI |
| DQ997439 | NCBI |  | CY023514 | NCBI |  | KF259362 | NCBI |  | AY180612 | fludb |
| FJ793373 | NCBI |  | CY023658 | NCBI |  | KF188319 | NCBI |  | AF523511 | NCBI |
| GQ335467 | NCBI |  | CY023690 | NCBI |  | KF259332 | NCBI |  | KF260118 | NCBI |
| DQ997462 | NCBI |  | CY023666 | fludb |  | JN653657 | NCBI |  | EU250007 | NCBI |
| DQ997478 | NCBI |  | KF259508 | fludb |  | KC821184 | NCBI |  | CY023964 | NCBI |
| FJ793293 | NCBI |  | KF259507 | NCBI |  | EF154992 | NCBI |  | AF508726 | NCBI |
| FJ793365 | NCBI |  | KF259498 | NCBI |  | AF523482 | NCBI |  | CY024028 | NCBI |
| FJ793285 | NCBI |  | JN627141 | NCBI |  | GQ373126 | NCBI |  | GQ373152 | NCBI |
| FJ793317 | NCBI |  | KF259524 | NCBI |  | KF715247 | NCBI |  | CY023692 | NCBI |
| EU753323 | NCBI |  | CY023498 | NCBI |  | CY023715 | NCBI |  | EF155224 | NCBI |
| DQ997469 | NCBI |  | KF259509 | fludb |  | CY023763 | NCBI |  | CY024260 | fludb |
| CY023753 | NCBI |  | CY023522 | NCBI |  | EF155032 | NCBI |  | CY024460 | NCBI |
| DQ997507 | NCBI |  | KF259493 | NCBI |  | KF259399 | NCBI |  | CY006019 | NCBI |
| JF519787 | NCBI |  | EU644489 | fludb |  | CY023499 | NCBI |  | KF260121 | NCBI |
| EU753315 | fludb |  | KF259474 | NCBI |  | EF155039 | NCBI |  | GQ373144 | NCBI |
| FJ793389 | NCBI |  | KF259506 | NCBI |  | KC821185 | NCBI |  | CY023668 | NCBI |
| EU753331 | NCBI |  | KF259479 | NCBI |  | KF259342 | NCBI |  | CY024636 | NCBI |
| FJ793421 | NCBI |  | EU216101 | NCBI |  | FJ581432 | NCBI |  | CY023348 | NCBI |
| DQ997422 | NCBI |  | KF188398 | NCBI |  | KF259327 | NCBI |  | CY023748 | NCBI |
| KF142482 | fludb |  | EU216102 | NCBI |  | EF155004 | NCBI |  | CY024012 | NCBI |
| DQ681212 | NCBI |  | KF259480 | NCBI |  | GU474553 | NCBI |  | KF260072 | NCBI |
| CY023225 | NCBI |  | KF259486 | NCBI |  | GQ373125 | NCBI |  | KF260092 | NCBI |
| DQ681208 | NCBI |  | KF259492 | NCBI |  | CY024387 | NCBI |  | CY024276 | fludb |
| CY024129 | NCBI |  | KF259478 | NCBI |  | KC951125 | NCBI |  | KF260073 | NCBI |
| CY024177 | NCBI |  | KF259471 | NCBI |  | KC821187 | NCBI |  | EU502896 | NCBI |
| DQ681222 | NCBI |  | EU216097 | NCBI |  | JN653669 | NCBI |  | CY023780 | fludb |
| CY023217 | NCBI |  | KF259487 | NCBI |  | KF259351 | NCBI |  | CY024076 | NCBI |
| DQ997492 | NCBI |  | KF259489 | NCBI |  | EF154994 | NCBI |  | EF155211 | NCBI |
| AY664727 | NCBI |  | KF259490 | fludb |  | KC821188 | NCBI |  | KF260086 | NCBI |
| DQ226142 | NCBI |  | KF259481 | NCBI |  | KF259388 | NCBI |  | EF155260 | fludb |
| CY023993 | fludb |  | EU216100 | NCBI |  | KF259328 | NCBI |  | CY023484 | NCBI |
| CY024201 | NCBI |  | KF259470 | NCBI |  | KF746772 | NCBI |  | DQ226118 | NCBI |
| EF155151 | NCBI |  | EU216094 | NCBI |  | KC821196 | NCBI |  | AY664748 | NCBI |
| DQ226146 | NCBI |  | KF259482 | NCBI |  | AF222664 | NCBI |  | CY024540 | NCBI |
| CY024025 | NCBI |  | KF259488 | NCBI |  | KF746782 | NCBI |  | CY023396 | NCBI |
| CY023241 | NCBI |  | EU216099 | NCBI |  | KC821192 | NCBI |  | EF155245 | NCBI |
| CY024105 | fludb |  | EU644489 | NCBI |  | KC821186 | NCBI |  | DQ981558 | NCBI |
| CY024009 | fludb |  | EU216104 | NCBI |  | KC821197 | NCBI |  | CY023148 | NCBI |
| FJ384752 | NCBI |  | EU644483 | NCBI |  | JQ901685 | NCBI |  | CY024236 | NCBI |
| CY023961 | NCBI |  | EU216103 | NCBI |  | CY023811 | NCBI |  | CY023652 | fludb |
| EF155152 | fludb |  | AF156397 | NCBI |  | HM751189 | NCBI |  | AF523514 | NCBI |
| CY023625 | NCBI |  | AF156398 | NCBI |  | AF523484 | NCBI |  | CY024044 | NCBI |
| CY023257 | fludb |  | EF155073 | NCBI |  | KC821194 | NCBI |  | CY024492 | NCBI |
| EF155169 | NCBI |  | AF156396 | NCBI |  | JQ356890 | NCBI |  | EU086256 | NCBI |
| CY023265 | NCBI |  | EF155085 | fludb |  | GU474609 | NCBI |  | EF155223 | NCBI |
| AF523415 | NCBI |  | EF155084 | NCBI |  | CY023427 | NCBI |  | KF260132 | fludb |
| DQ997189 | NCBI |  | EF155059 | NCBI |  | EU835750 | NCBI |  | DQ064467 | NCBI |
| EU365372 | NCBI |  | CY024562 | NCBI |  | KC821193 | NCBI |  | EF155246 | NCBI |
| CY023249 | NCBI |  | EF155069 | NCBI |  | CY023747 | NCBI |  | CY024220 | NCBI |
| DQ681204 | NCBI |  | KF188324 | NCBI |  | KC821191 | NCBI |  | AF222825 | NCBI |
| CY023537 | NCBI |  | EF155060 | NCBI |  | KC821190 | NCBI |  | CY023276 | NCBI |
| EU753275 | NCBI |  | EF155055 | NCBI |  | EF155034 | NCBI |  | CY024380 | fludb |
| CY024241 | NCBI |  | AF222656 | fludb |  | EU882860 | NCBI |  | CY023716 | NCBI |
| CY024169 | NCBI |  | GU053180 | NCBI |  | KC417050 | NCBI |  | AF523516 | NCBI |
| EF155150 | NCBI |  | EF155123 | NCBI |  | EF155010 | NCBI |  | CY023356 | NCBI |
| CY023345 | NCBI |  | EF155054 | NCBI |  | KF259343 | NCBI |  | KF260126 | fludb |
| EF155184 | NCBI |  | EF155087 | NCBI |  | KC821189 | NCBI |  | CY023220 | NCBI |
| EF155182 | NCBI |  | EF155072 | NCBI |  | GQ373128 | NCBI |  | CY024436 | NCBI |
| CY024441 | NCBI |  | AF222655 | fludb |  | KC821195 | NCBI |  | CY023468 | NCBI |
| CY023601 | fludb |  | EF155095 | NCBI |  | CY024467 | NCBI |  | CY023252 | NCBI |
| KF259835 | NCBI |  | EF155121 | NCBI |  | EF155016 | NCBI |  | DQ064479 | NCBI |
| CY023609 | fludb |  | EF155068 | NCBI |  | CY024083 | NCBI |  | CY023628 | fludb |
| KF259840 | NCBI |  | EF155076 | NCBI |  | KC768044 | NCBI |  | DQ064465 | NCBI |
| EF155183 | NCBI |  | EF155070 | NCBI |  | KC821201 | NCBI |  | JF795120 | NCBI |
| CY024513 | NCBI |  | AF222653 | NCBI |  | KC821200 | NCBI |  | CY024252 | NCBI |
| CY024417 | fludb |  | EF155083 | NCBI |  | KF259386 | NCBI |  | KF260098 | fludb |
| CY023593 | fludb |  | EF155058 | NCBI |  | KF259329 | NCBI |  | CY023364 | NCBI |
| CY023553 | NCBI |  | EF155063 | NCBI |  | KJ128363 | NCBI |  | CY024116 | NCBI |
| CY024337 | NCBI |  | EF155125 | NCBI |  | KF259373 | NCBI |  | CY024308 | NCBI |
| CY024409 | NCBI |  | EF155088 | NCBI |  | KF259377 | NCBI |  | CY024364 | fludb |
| CY023545 | fludb |  | AF222652 | NCBI |  | KC821199 | NCBI |  | KF260075 | NCBI |
| CY023377 | fludb |  | CY023098 | NCBI |  | EF155040 | NCBI |  | KF260082 | NCBI |
| CY024353 | NCBI |  | EF155120 | NCBI |  | KF259372 | NCBI |  | CY023580 | fludb |
| CY023369 | NCBI |  | EF155082 | NCBI |  | KF259355 | NCBI |  | EF155238 | NCBI |
| CY024329 | NCBI |  | AF222654 | NCBI |  | GQ373134 | NCBI |  | KF260093 | NCBI |
| CY023585 | fludb |  | EF155067 | NCBI |  | KC821209 | NCBI |  | EF155262 | NCBI |
| CY023393 | NCBI |  | CY024554 | NCBI |  | KF259405 | NCBI |  | CY023836 | NCBI |
| CY023441 | NCBI |  | AJ404629 | NCBI |  | EF155046 | NCBI |  | CY024092 | NCBI |
| CY023353 | NCBI |  | EF155064 | NCBI |  | JF795057 | NCBI |  | CY024516 | NCBI |
| CY023561 | NCBI |  | EF155101 | NCBI |  | EF155019 | NCBI |  | DQ064464 | NCBI |
| HQ425336 | NCBI |  | EF155108 | NCBI |  | EF155025 | NCBI |  | CY023172 | NCBI |
| CY023577 | NCBI |  | EF155106 | NCBI |  | EF155001 | NCBI |  | CY023228 | NCBI |
| CY024433 | NCBI |  | EF155104 | NCBI |  | CY023491 | NCBI |  | CY023420 | NCBI |
| EF155188 | fludb |  | EF155107 | NCBI |  | JQ901691 | NCBI |  | CY024284 | NCBI |
| CY023569 | fludb |  | EF155094 | NCBI |  | CY023363 | NCBI |  | CY023180 | NCBI |
| CY023401 | NCBI |  | EF155098 | fludb |  | KF259396 | NCBI |  | KF260089 | NCBI |
| CY024457 | NCBI |  | EF155102* | fludb |  | KF259438 | NCBI |  | KF260105 | NCBI |
| CY023433 | NCBI |  | EF155105 | NCBI |  | EF155047 | NCBI |  | CY024244 | NCBI |
| CY023465 | NCBI |  | DQ064433 | NCBI |  | GQ373131 | NCBI |  | AF222824 | NCBI |
| EF155186 | fludb |  | DQ874395 | NCBI |  | KF259440 | NCBI |  | CY023244 | NCBI |
| CY023473 | NCBI |  | DQ997491 | NCBI |  | KF500978 | NCBI |  | DQ485212 | NCBI |
| CY024393 | NCBI |  | FJ793286 | NCBI |  | KF259400 | NCBI |  | CY023644 | NCBI |
| CY024345 | NCBI |  | DQ997429 | NCBI |  | CY063665 | NCBI |  | CY024444 | NCBI |
| CY024361 | NCBI |  | JF916723 | NCBI |  | CY023275 | NCBI |  | CY024156 | NCBI |
| EF155187 | NCBI |  | JQ639780 | NCBI |  | EF155013 | NCBI |  | GQ373150 | NCBI |
| KF259838 | NCBI |  | DQ997441 | NCBI |  | KC821205 | NCBI |  | CY023444 | NCBI |
| EF155185 | NCBI |  | DQ064431 | fludb |  | KC821207 | NCBI |  | CY024188 | NCBI |
| CY024521 | NCBI |  | AF536711 | NCBI |  | KC821210 | NCBI |  | EF155268 | NCBI |
| KF259828 | NCBI |  | AF222813 | NCBI |  | JQ356891 | NCBI |  | JF795114 | NCBI |
| CY023657 | fludb |  | FJ793398 | NCBI |  | EF154985 | NCBI |  | CY023620 | NCBI |
| KF259842 | NCBI |  | AY262366 | NCBI |  | KC821211 | NCBI |  | CY023684 | NCBI |
| CY023681 | NCBI |  | DQ681205 | NCBI |  | GU474593 | NCBI |  | KF260087 | NCBI |
| CY023617 | NCBI |  | DQ064414 | NCBI |  | KF259338 | NCBI |  | DQ226121 | NCBI |
| KF259833 | NCBI |  | FJ793438 | fludb |  | EF154980 | NCBI |  | EF155264 | NCBI |
| CY023297 | NCBI |  | DQ064430 | NCBI |  | CY024043 | NCBI |  | KF260078 | NCBI |
| FJ547483 | NCBI |  | JF916715 | NCBI |  | EF155030 | NCBI |  | KF260103 | NCBI |
| KF259829 | NCBI |  | AF508587 | NCBI |  | CY023523 | NCBI |  | KF260081 | NCBI |
| KF746778 | NCBI |  | DQ681209 | NCBI |  | KF259381 | NCBI |  | CY024572 | NCBI |
| CY023505 | NCBI |  | HQ425335 | NCBI |  | EF154999 | NCBI |  | GQ373136 | NCBI |
| KF259831 | NCBI |  | DQ064427 | NCBI |  | KC821198 | NCBI |  | EF155243 | NCBI |
| KF259837 | NCBI |  | JF795138 | NCBI |  | KF259361 | NCBI |  | KF260104 | NCBI |
| EF155191 | fludb |  | DQ997452 | NCBI |  | JF795049 | NCBI |  | DQ981598 | NCBI |
| EF155192 | NCBI |  | EU346936 | NCBI |  | JN653656 | NCBI |  | KF260090 | NCBI |
| CY023481 | NCBI |  | DQ997482 | NCBI |  | JN653661 | NCBI |  | EF155235 | NCBI |
| CY023633 | NCBI |  | DQ997468 | NCBI |  | KC821203 | NCBI |  | AY664751 | NCBI |
| CY023641 | fludb |  | DQ997477 | NCBI |  | CY146663 | NCBI |  | CY024484 | NCBI |
| CY023521 | NCBI |  | DQ997499 | NCBI |  | EF155002 | NCBI |  | EF155212 | NCBI |
| JF795124 | NCBI |  | AF536713 | NCBI |  | EF154983 | NCBI |  | EF155210 | fludb |
| FJ492973 | NCBI |  | KF746812 | NCBI |  | KF259455 | NCBI |  | KF260088 | NCBI |
| CY024041 | fludb |  | AF508585 | NCBI |  | KC821204 | NCBI |  | EF155221 | fludb |
| EF155190 | NCBI |  | AF536709 | NCBI |  | KF259331 | NCBI |  | DQ064466 | NCBI |
| KF259834 | NCBI |  | AF508592 | NCBI |  | KF746822 | NCBI |  | CY023412 | NCBI |
| KF259843 | NCBI |  | FJ793310 | NCBI |  | KC821202 | NCBI |  | AY664752 | NCBI |
| KF259832 | NCBI |  | AF523402 | NCBI |  | AJ278647 | NCBI |  | CY024428 | NCBI |
| CY023673 | fludb |  | AF222812 | NCBI |  | GQ373132 | NCBI |  | DQ064480 | NCBI |
| KF259830 | NCBI |  | EU935061 | NCBI |  | CY023643 | NCBI |  | EF155265 | fludb |
| CY023689 | fludb |  | FJ793406 | fludb |  | GQ373127 | NCBI |  | CY024100 | NCBI |
| CY023497 | NCBI |  | DQ681217 | NCBI |  | KF259391 | NCBI |  | CY023212 | NCBI |
| CY023649 | fludb |  | AF536712 | NCBI |  | JF795073 | NCBI |  | DQ226119 | NCBI |
| CY023489 | NCBI |  | DQ064428 | NCBI |  | JF795041 | NCBI |  | CY024124 | NCBI |
| KF746868 | NCBI |  | EU753332 | NCBI |  | CY024315 | NCBI |  | EU502904 | NCBI |
| EF155189 | NCBI |  | FJ384753 | NCBI |  | EF155042 | NCBI |  | KF260113 | fludb |
| KF259827 | NCBI |  | DQ997438 | NCBI |  | GQ202051 | NCBI |  | EF155227 | NCBI |
| FJ492974 | NCBI |  | CY023210 | NCBI |  | EF154981 | NCBI |  | KF260117 | NCBI |
| CY023665 | NCBI |  | AY664701 | fludb |  | CY023683 | NCBI |  | EF155242 | NCBI |
| CY023513 | NCBI |  | AY253754 | NCBI |  | HM998917 | NCBI |  | CY023956 | NCBI |
| CY023529 | NCBI |  | EF626679 | NCBI |  | KC768043 | NCBI |  | AF523512 | NCBI |
| JF795118 | NCBI |  | EU346940 | NCBI |  | KC821206 | NCBI |  | CY023156 | NCBI |
| JQ228393 | NCBI |  | DQ681213 | NCBI |  | JF795065 | NCBI |  | CY024332 | NCBI |
| CY023777 | NCBI |  | AY664702 | fludb |  | KF059284 | NCBI |  | EF155219 | NCBI |
| KF259853 | fludb |  | DQ226136 | NCBI |  | KF059285 | NCBI |  | DQ981574 | NCBI |
| KF259864 | NCBI |  | EU753284 | NCBI |  | AF523483 | NCBI |  | CY023500 | NCBI |
| KF259845 | NCBI |  | FJ793390 | NCBI |  | KF259445 | NCBI |  | KF367735 | NCBI |
| GQ373096* | fludb |  | EU753276 | NCBI |  | KF059286 | NCBI |  | EF155232 | NCBI |
| KF259852 | NCBI |  | KF142483 | NCBI |  | KF259382 | NCBI |  | EU516315 | NCBI |
| CY023737 | NCBI |  | AY664710 | NCBI |  | JN653655 | NCBI |  | EF155237 | NCBI |
| KF259858 | NCBI |  | AY664700 | NCBI |  | KF059287 | NCBI |  | CY023588 | NCBI |
| KF259865 | NCBI |  | DQ064432 | NCBI |  | EF155015 | NCBI |  | JF795132 | NCBI |
| GQ373094 | NCBI |  | DQ226135 | NCBI |  | EF155035 | NCBI |  | CY023764 | NCBI |
| KF259841 | NCBI |  | EU753324 | NCBI |  | CY024737 | NCBI |  | CY024532 | fludb |
| KF259870 | NCBI |  | DQ997506 | NCBI |  | FJ499466 | NCBI |  | CY023892 | NCBI |
| GQ373100 | NCBI |  | EU753316 | NCBI |  | GQ373124 | NCBI |  | DQ064477 | fludb |
| GQ373085 | NCBI |  | DQ997461 | NCBI |  | CY024211 | NCBI |  | DQ226117 | NCBI |
| CY023833 | NCBI |  | EU753308 | NCBI |  | KF059288 | NCBI |  | EF155270 | NCBI |
| CY023793 | fludb |  | DQ997188 | NCBI |  | CY024259 | NCBI |  | KF260116 | NCBI |
| KF259844 | NCBI |  | GQ335472 | NCBI |  | CY023099 | NCBI |  | CY023788 | fludb |
| KF259846 | NCBI |  | AY664707 | NCBI |  | CY024427 | NCBI |  | CY024132 | NCBI |
| EU086253 | NCBI |  | DQ997421 | NCBI |  | KF259346 | NCBI |  | DQ981542 | NCBI |
| CY023761 | NCBI |  | EU346937 | NCBI |  | KF259326 | NCBI |  | CY024316 | NCBI |
| CY023817 | NCBI |  | AY664711 | NCBI |  | DQ107516 | NCBI |  | KF260119 | NCBI |
| GQ373095 | NCBI |  | EU365373 | NCBI |  | CY023835 | NCBI |  | CY024324 | NCBI |
| GQ373099 | NCBI |  | AY664698 | NCBI |  | EF154986 | NCBI |  | DQ981590 | NCBI |
| GQ373097 | NCBI |  | FJ793294 | fludb |  | JF916708 | NCBI |  | JN222385 | NCBI |
| JN869517 | NCBI |  | EF626678 | NCBI |  | KF007015 | NCBI |  | EF155225 | NCBI |
| GQ373092 | NCBI |  | DQ226137 | NCBI |  | JF906209 | NCBI |  | AF523513 | NCBI |
| JF916706 | NCBI |  | CY023754 | NCBI |  | CY023515 | NCBI |  | EF155256 | NCBI |
| KF259839 | NCBI |  | EU753300 | NCBI |  | KF059289 | NCBI |  | EF155214 | fludb |
| GQ373086 | NCBI |  | EU753292 | NCBI |  | KF059290 | NCBI |  | GQ373146* | fludb |
| KF259857 | NCBI |  | FJ793374 | NCBI |  | KF059291 | NCBI |  | CY024420 | NCBI |
| GQ373101 | NCBI |  | GQ335468 | NCBI |  | JN653662 | NCBI |  | CY023284 | NCBI |
| CY055157 | NCBI |  | GQ373109 | NCBI |  | CY024251 | NCBI |  | JF795126 | NCBI |
| KF259851 | NCBI |  | CY023794 | fludb |  | CY023483 | NCBI |  | KF260094 | fludb |
| JN222384 | NCBI |  | AY180813 | NCBI |  | JF795105 | NCBI |  | CY023492 | NCBI |
| KF259850 | NCBI |  | AY664699 | NCBI |  | EF155052 | NCBI |  | CY023532 | NCBI |
| CY023785 | fludb |  | GQ373117 | NCBI |  | EF154997 | NCBI |  | EF155208 | NCBI |
| KF259854 | fludb |  | GQ373113 | NCBI |  | KF259437 | NCBI |  | CY055160 | NCBI |
| KF259863 | NCBI |  | JF795112 | NCBI |  | CY024139 | NCBI |  | CY023164 | NCBI |
| CY023745 | NCBI |  | AF508583 | NCBI |  | KC417049 | NCBI |  | CY024735 | NCBI |
| KF259836 | NCBI |  | KF746789 | NCBI |  | JN653663 | NCBI |  | CY023404 | NCBI |
| KF259848 | NCBI |  | GU722377 | NCBI |  | KF059292 | NCBI |  | KC821251 | NCBI |
| KF259874 | NCBI |  | CY023834 | NCBI |  | KC779050 | NCBI |  | KF142485 | fludb |
| KF259859 | NCBI |  | GU722376 | NCBI |  | KF059293 | NCBI |  | JF795106 | NCBI |
| KF259862 | NCBI |  | GQ373111 | NCBI |  | KF714778 | NCBI |  | KF260146 | fludb |
| KF259888 | fludb |  | DQ064412 | NCBI |  | KM609600 | NCBI |  | JN869550 | NCBI |
| KF259873 | fludb |  | CY005502 | NCBI |  | CY087182 | NCBI |  | KC821253 | NCBI |
| KF259860 | NCBI |  | EU346935 | NCBI |  | JQ228392 | NCBI |  | KF260143 | fludb |
| KF259871 | NCBI |  | EU086250 | NCBI |  | KF714786 | NCBI |  | KC821254 | NCBI |
| KF259861 | NCBI |  | KF188390 | NCBI |  | GQ373130 | NCBI |  | KC821255 | NCBI |
| KF259875 | NCBI |  | FJ793430 | NCBI |  | KM609601 | NCBI |  | KC821252 | NCBI |
| KF259882 | fludb |  | AY180815 | fludb |  | CY024219 | NCBI |  | FJ793360 | NCBI |
| KF259855 | NCBI |  | AF536710 | NCBI |  | GU474601 | NCBI |  | KF260218 | NCBI |
| KF259881 | NCBI |  | GU722373 | NCBI |  | KM609602 | NCBI |  | KC821256 | NCBI |
| KF259883 | NCBI |  | AF222659 | NCBI |  | JN653664 | NCBI |  | JQ356894 | NCBI |
| KF259877 | NCBI |  | GQ373103 | NCBI |  | EF155024 | NCBI |  | JN653672 | NCBI |
| KF259849 | NCBI |  | DQ064424 | NCBI |  | KF259324 | NCBI |  | FJ793344 | NCBI |
| KF259856 | NCBI |  | CY023762 | NCBI |  | AF222662 | NCBI |  | KC821257 | NCBI |
| KF259876 | NCBI |  | EU346941 | NCBI |  | KF259345 | NCBI |  | EU250011 | NCBI |
| KF259872 | fludb |  | GU722378 | NCBI |  | KF259348 | NCBI |  | KF260220 | NCBI |
| KF259887 | fludb |  | GQ373104 | NCBI |  | KM609603 | NCBI |  | KF260168 | NCBI |
| KF259866 | NCBI |  | GQ373116 | NCBI |  | EU835746 | NCBI |  | KF260124 | NCBI |
| CY023193 | NCBI |  | GQ373102 | NCBI |  | JQ901689 | NCBI |  | EU753310 | NCBI |
| DQ064459 | NCBI |  | GQ373112* | fludb |  | KM609604 | NCBI |  | KC821258 | NCBI |
| EU086252 | NCBI |  | CY023778 | NCBI |  | KC768042 | NCBI |  | JF795058 | NCBI |
| CY023705 | NCBI |  | CY023746 | NCBI |  | KM609605 | NCBI |  | EU250013 | NCBI |
| AY664721 | fludb |  | AF508595 | NCBI |  | KM609606 | NCBI |  | DQ226126 | NCBI |
| DQ226149 | NCBI |  | GU722371 | NCBI |  | KM609607 | NCBI |  | FJ793408 | NCBI |
| DQ226147 | NCBI |  | EU753340 | NCBI |  | EF155049 | NCBI |  | JF795098 | NCBI |
| EU086251 | NCBI |  | KF746878 | NCBI |  | JF795081 | NCBI |  | JN653677 | NCBI |
| CY023209 | NCBI |  | AY664704 | NCBI |  | CY023667 | NCBI |  | GQ373143 | NCBI |
| EU086273 | NCBI |  | GQ373115 | NCBI |  | CY024051 | NCBI |  | GQ373141 | NCBI |
| AY664730 | NCBI |  | CY023786 | NCBI |  | EU835749 | NCBI |  | KF260210 | NCBI |
| EU516308 | NCBI |  | CY023738 | NCBI |  | EF154990 | NCBI |  | JQ356893 | NCBI |
| EU753299 | NCBI |  | AY043023 | NCBI |  | KF259436 | NCBI |  | KC821259 | NCBI |
| AY664729 | fludb |  | DQ465402 | fludb |  | CY024555 | NCBI |  | GQ202038 | NCBI |
| AY664717 | NCBI |  | AY043022 | NCBI |  | CY024331 | NCBI |  | KC821261 | NCBI |
| EU753291 | NCBI |  | KF188277 | NCBI |  | GQ373119 | NCBI |  | KF260219 | fludb |
| DQ907704 | NCBI |  | DQ064422 | NCBI |  | CY087190 | NCBI |  | JN653679 | NCBI |
| GQ477296 | NCBI |  | AF222657 | NCBI |  | CY024147 | NCBI |  | EU753278 | NCBI |
| CY023697 | NCBI |  | AF536714 | NCBI |  | CY023291 | NCBI |  | KF260128 | NCBI |
| AY664719 | NCBI |  | EU346934 | NCBI |  | JN869541 | NCBI |  | FJ581433 | NCBI |
| DQ064442 | NCBI |  | KF746764 | NCBI |  | KF259354 | NCBI |  | EU935069 | NCBI |
| EU753283 | NCBI |  | AY180821 | NCBI |  | KM609608 | NCBI |  | EU532050 | NCBI |
| AY664720 | fludb |  | AY262367 | NCBI |  | KF259390 | NCBI |  | KC821262 | NCBI |
| EU086274 | NCBI |  | AF536715 | NCBI |  | JN653660 | NCBI |  | KC417058 | NCBI |
| EU086309 | NCBI |  | AY180826 | fludb |  | KM609609 | NCBI |  | KC821270 | NCBI |
| EF612745 | NCBI |  | EF070734 | NCBI |  | CY023795 | NCBI |  | JN653676 | NCBI |
| CY023721 | NCBI |  | AY180822 | fludb |  | KM609610 | NCBI |  | KC821266 | NCBI |
| EU935060 | NCBI |  | AF222660 | NCBI |  | CY024563 | NCBI |  | DQ064469 | NCBI |
| EF155154 | NCBI |  | AF156394 | NCBI |  | JN869519 | NCBI |  | GQ373140 | NCBI |
| EU086289 | NCBI |  | EU346939 | NCBI |  | KM609611 | NCBI |  | KC821260 | NCBI |
| AY664718 | NCBI |  | AF508586 | NCBI |  | KF259340 | NCBI |  | EU250014 | NCBI |
| CY023729 | NCBI |  | AF222658 | NCBI |  | KM609612 | NCBI |  | KF746773 | NCBI |
| EU753307 | NCBI |  | KF746829 | NCBI |  | KM609613 | NCBI |  | AY664740 | fludb |
| DQ226148 | NCBI |  | FJ793414 | NCBI |  | CY024507 | NCBI |  | EU532052 | NCBI |
| EU086229 | NCBI |  | AF536716 | NCBI |  | CY024163 | NCBI |  | KC821271 | NCBI |
| FJ793325 | NCBI |  | CY005508 | NCBI |  | KF259336 | NCBI |  | KF260217 | NCBI |
| AY664726 | fludb |  | AF508589 | NCBI |  | EF155021 | NCBI |  | JN653685 | NCBI |
| CY023769 | NCBI |  | AY180824 | fludb |  | JF795147 | NCBI |  | EU086292 | fludb |
| KF259898 | NCBI |  | DQ064409 | NCBI |  | KM609614 | NCBI |  | KF260203 | NCBI |
| FJ499469 | NCBI |  | AF536717 | fludb |  | KM609615 | NCBI |  | EU532045 | fludb |
| FJ793301 | NCBI |  | AF222661 | NCBI |  | EF155018 | NCBI |  | KF260204 | NCBI |
| HM590763 | fludb |  | KF746837 | NCBI |  | KF259347 | NCBI |  | KF260202 | NCBI |
| EU753339 | NCBI |  | AF508588 | NCBI |  | AJ427865 | NCBI |  | KF260164 | NCBI |
| EU532042 | NCBI |  | CY077086 | NCBI |  | CY023403 | NCBI |  | KC768052 | NCBI |
| GQ202048 | NCBI |  | AY043020 | NCBI |  | KC779049 | NCBI |  | KF260137 | NCBI |
| KF259897 | fludb |  | DQ064429 | NCBI |  | JN653667 | NCBI |  | KF260155 | fludb |
| EU086290 | NCBI |  | KF188273 | fludb |  | KM609616 | NCBI |  | KF746781 | NCBI |
| GQ335473 | NCBI |  | DQ064419 | NCBI |  | EF155051 | NCBI |  | GQ202044 | fludb |
| GQ335515 | NCBI |  | CY023706 | NCBI |  | KF259358 | NCBI |  | KC779055 | NCBI |
| KF746796 | NCBI |  | EU086306 | NCBI |  | EF155014 | NCBI |  | FJ793376 | NCBI |
| CY023825 | NCBI |  | EU086323 | NCBI |  | DQ226095 | NCBI |  | KF260138 | NCBI |
| FJ793357 | NCBI |  | CY023722 | NCBI |  | CY024075 | NCBI |  | EU753294 | NCBI |
| EU086308 | NCBI |  | CY075040 | NCBI |  | KF259360 | NCBI |  | KF260201 | NCBI |
| GQ202042 | NCBI |  | GU722370 | NCBI |  | GQ373133 | NCBI |  | KC768053 | NCBI |
| FJ793445 | NCBI |  | EU086271 | NCBI |  | KF259403 | NCBI |  | KC821268 | NCBI |
| GQ373088 | NCBI |  | GU722372 | NCBI |  | GQ373123 | NCBI |  | EU935068 | NCBI |
| GQ335507 | NCBI |  | GU722374 | NCBI |  | CY024123 | NCBI |  | JF795074 | NCBI |
| KF259896 | NCBI |  | FJ793358 | NCBI |  | CY023827 | NCBI |  | KF178699 | NCBI |
| EU753347 | NCBI |  | EF612744 | NCBI |  | KM609617 | NCBI |  | EU086277 | NCBI |
| KF259869 | fludb |  | EU086270 | NCBI |  | HM751157 | NCBI |  | KC821267 | NCBI |
| FJ793397 | NCBI |  | HM590774 | NCBI |  | AF156463 | NCBI |  | KC821265 | NCBI |
| CY063663 | NCBI |  | HM590764 | NCBI |  | JQ356892 | NCBI |  | KF714787 | NCBI |
| GQ335491 | NCBI |  | CY023770 | NCBI |  | GQ373120 | NCBI |  | KC821264 | NCBI |
| FJ534544 | NCBI |  | EU086249 | NCBI |  | CY024115 | NCBI |  | CY023724 | fludb |
| KF259867 | NCBI |  | GU722379 | NCBI |  | KF259365 | NCBI |  | DQ997440 | NCBI |
| FJ793349 | NCBI |  | CY075056 | NCBI |  | EF155011 | NCBI |  | DQ226123 | NCBI |
| JF519788 | NCBI |  | DQ227353 | NCBI |  | KF259446 | NCBI |  | KC821263 | NCBI |
| KF259899 | NCBI |  | DQ064421 | NCBI |  | CY024195 | NCBI |  | KF260154 | NCBI |
| GQ373098 | NCBI |  | GU722380 | NCBI |  | KF259385 | NCBI |  | KF260152 | fludb |
| EU086325 | NCBI |  | EU086272 | NCBI |  | KF259344 | NCBI |  | KC821269 | NCBI |
| KF259902 | NCBI |  | DQ000239 | fludb |  | KF259330 | NCBI |  | EU753350 | NCBI |
| FJ793333 | NCBI |  | EU086322 | NCBI |  | GQ202059 | NCBI |  | EF612746 | fludb |
| KF367732 | NCBI |  | EU086307 | fludb |  | KF259315 | NCBI |  | KF260153 | NCBI |
| KF259868 | NCBI |  | EU086228 | NCBI |  | KM609618 | NCBI |  | KC821275 | NCBI |
| FJ793341 | NCBI |  | DQ064418 | NCBI |  | KM609619 | NCBI |  | KF260136 | NCBI |
| CY023841 | fludb |  | CY075032 | NCBI |  | KF259450 | NCBI |  | KC821281 | NCBI |
| GU121383 | NCBI |  | GU121384 | NCBI |  | CY024403 | NCBI |  | EU086254 | NCBI |
| EU086324 | NCBI |  | CY023730 | NCBI |  | CY023891 | NCBI |  | KF260142 | NCBI |
| HM590771 | fludb |  | EU086287 | NCBI |  | AF255364 | NCBI |  | KF260161 | fludb |
| KC821140 | NCBI |  | HQ117888 | NCBI |  | KC464601 | NCBI |  | EU250008 | NCBI |
| JF906207 | NCBI |  | CY075048 | NCBI |  | EF155043 | NCBI |  | KF260150 | NCBI |
| KC821142 | NCBI |  | EU086236 | NCBI |  | CY023323 | NCBI |  | EU086326 | NCBI |
| JN653626 | NCBI |  | GU722375 | NCBI |  | KM609620 | NCBI |  | KF746791 | NCBI |
| JN653625 | fludb |  | CY023698 | NCBI |  | EF155038 | NCBI |  | EU086327 | fludb |
| KC821144 | NCBI |  | EF626680 | NCBI |  | KF259374 | NCBI |  | KF746863 | NCBI |
| JF795079 | NCBI |  | DQ981628 | NCBI |  | JN869526 | NCBI |  | KC821279 | NCBI |
| KC821143 | NCBI |  | FJ793366 | NCBI |  | KF259441 | NCBI |  | KF746806 | NCBI |
| GU474567 | NCBI |  | DQ981612 | NCBI |  | KF259341 | NCBI |  | KF260145 | NCBI |
| FJ492972 | NCBI |  | AY180817 | fludb |  | KF259397 | NCBI |  | KF260160 | NCBI |
| KC821141 | NCBI |  | EU429699 | NCBI |  | JF795097 | NCBI |  | GQ477302 | NCBI |
| KF714784 | NCBI |  | CY023314 | NCBI |  | CY023507 | NCBI |  | KF260139 | NCBI |
| JN653636 | NCBI |  | FJ793350 | NCBI |  | KM609621 | NCBI |  | KC821280 | NCBI |
| GU474551 | NCBI |  | CY023330 | NCBI |  | KF367734 | NCBI |  | KF260163 | NCBI |
| KC821145 | NCBI |  | FJ793342 | NCBI |  | KF746805 | NCBI |  | CY087175 | NCBI |
| JN653628 | NCBI |  | EF155057 | NCBI |  | AF222666 | NCBI |  | GU474602 | NCBI |
| KC821146 | NCBI |  | DQ981604 | NCBI |  | JF795089 | NCBI |  | FJ534540 | NCBI |
| GQ202054 | fludb |  | CY024586 | NCBI |  | KM609622 | NCBI |  | JQ356895 | NCBI |
| JN653633 | NCBI |  | CY023978 | NCBI |  | EF155045 | NCBI |  | KC821274 | NCBI |
| KC821147 | NCBI |  | CY024578 | NCBI |  | JN869533 | NCBI |  | AY664737 | NCBI |
| GQ202062 | NCBI |  | DQ981620 | NCBI |  | CY024531 | NCBI |  | HM751158 | NCBI |
| KC821148 | NCBI |  | DQ981580 | NCBI |  | EF155009 | NCBI |  | KC821277 | NCBI |
| GU474575 | NCBI |  | CY024474 | NCBI |  | KM609623 | NCBI |  | EU532046 | NCBI |
| KF714776 | NCBI |  | CY005526 | NCBI |  | JN222383 | NCBI |  | CY023820 | NCBI |
| KC821152 | NCBI |  | EU429725 | NCBI |  | CY024003 | NCBI |  | DQ981606 | NCBI |
| GU474591 | NCBI |  | AF536718 | NCBI |  | KM609624 | NCBI |  | KF500981 | NCBI |
| KC821153 | NCBI |  | EU429707 | NCBI |  | GU474577 | NCBI |  | JN571295 | NCBI |
| KC821158 | NCBI |  | EF155056 | NCBI |  | KF178690 | NCBI |  | CY023196 | NCBI |
| KC821155 | NCBI |  | EF155061 | NCBI |  | KF259392 | NCBI |  | EU753326 | NCBI |
| JN653630 | NCBI |  | AY180816 | NCBI |  | JN653654 | NCBI |  | JF906210 | NCBI |
| GU474583 | NCBI |  | GQ477299 | NCBI |  | KM609625 | NCBI |  | JN653675 | fludb |
| JN653622 | fludb |  | CY023338 | NCBI |  | AY043025 | NCBI |  | DQ226125 | NCBI |
| JF795095 | NCBI |  | FJ793382 | NCBI |  | JN653659 | NCBI |  | KF260215 | NCBI |
| KF259969 | NCBI |  | CY024610 | NCBI |  | KF178682 | NCBI |  | AY664739 | NCBI |
| JF795145 | NCBI |  | CY024594 | fludb |  | CY087174 | NCBI |  | FJ793352 | NCBI |
| KC821151 | NCBI |  | EU516305 | NCBI |  | KF259453 | NCBI |  | HM998926 | NCBI |
| JN653631 | NCBI |  | FJ793318 | NCBI |  | AF508699 | NCBI |  | DQ981582 | NCBI |
| KF715237 | NCBI |  | CY024602 | NCBI |  | EF154996 | NCBI |  | GU474586 | NCBI |
| GQ373089 | NCBI |  | AF523401 | NCBI |  | AF523485 | NCBI |  | KC821276 | NCBI |
| FJ547484 | NCBI |  | GQ477298 | NCBI |  | CY023539 | NCBI |  | KF260151 | fludb |
| KC768048 | NCBI |  | CY023306 | NCBI |  | KF259359 | NCBI |  | KC821272 | NCBI |
| EU414523 | NCBI |  | CY023458 | NCBI |  | KM609626 | NCBI |  | KC417060 | NCBI |
| EU414524 | NCBI |  | CY023154 | NCBI |  | EU532033 | NCBI |  | KC821278 | NCBI |
| JF519793 | NCBI |  | FJ793326 | NCBI |  | KF259350 | NCBI |  | CY023732 | NCBI |
| JF519792 | NCBI |  | CY024570 | NCBI |  | KF259325 | NCBI |  | KF746815 | NCBI |
| HM751195 | NCBI |  | GQ373114 | NCBI |  | CY023843 | NCBI |  | JN869534 | NCBI |
| KF259966 | NCBI |  | EU753348 | NCBI |  | KM609627 | NCBI |  | CY087191 | NCBI |
| KF259970 | NCBI |  | FJ793302 | NCBI |  | EF154998 | NCBI |  | JN653683 | NCBI |
| GQ335482 | NCBI |  | EF626682 | NCBI |  | EU086248 | NCBI |  | KC821273 | NCBI |
| JF519794 | NCBI |  | GQ202037 | NCBI |  | HM751197 | NCBI |  | KF260156 | fludb |
| KF259964 | NCBI |  | KF746797 | NCBI |  | CY024299 | NCBI |  | KC821283 | NCBI |
| JF795055 | NCBI |  | EU216107 | NCBI |  | KF259404 | NCBI |  | EU086276 | fludb |
| KF259973 | NCBI |  | KC821214 | NCBI |  | DQ226096 | NCBI |  | KC821284 | NCBI |
| KC821159 | NCBI |  | FJ793446 | NCBI |  | JX312543 | NCBI |  | DQ981622 | NCBI |
| KF259968 | fludb |  | GQ373107 | NCBI |  | CY024539 | NCBI |  | CY023844 | NCBI |
| KF259967 | fludb |  | FJ534551 | fludb |  | CY024307 | NCBI |  | KC821282 | NCBI |
| HM751155 | NCBI |  | FJ534539 | NCBI |  | CY024235 | NCBI |  | JN653671 | NCBI |
| GQ373087 | NCBI |  | FJ499464 | NCBI |  | KM609628 | NCBI |  | KC821285 | NCBI |
| KF715229 | NCBI |  | KC821216 | NCBI |  | KM609629 | NCBI |  | KF746823 | NCBI |
| JF519790 | NCBI |  | FJ534547 | NCBI |  | CY023387 | NCBI |  | FJ793440 | NCBI |
| KF746749 | NCBI |  | KC821218 | NCBI |  | KM609630 | NCBI |  | KC779056 | NCBI |
| FJ492971 | NCBI |  | HQ398362 | NCBI |  | KC417051 | NCBI |  | KC417059 | NCBI |
| KF746844 | NCBI |  | GQ202057 | NCBI |  | JN653658 | NCBI |  | KJ128366 | NCBI |
| KF746770 | NCBI |  | KC821217 | NCBI |  | GQ373122 | NCBI |  | KF260199 | NCBI |
| JF519791 | NCBI |  | KC821215 | NCBI |  | KF259352 | NCBI |  | KF260212 | fludb |
| HM751187 | NCBI |  | HQ326723 | NCBI |  | CY024227 | NCBI |  | KF059314 | NCBI |
| JF519789 | NCBI |  | FJ793422 | NCBI |  | HM998925 | NCBI |  | KF260141 | fludb |
| JF795039 | NCBI |  | HQ398347 | NCBI |  | KF259323 | NCBI |  | KF059315 | NCBI |
| KF259979 | NCBI |  | FJ534549 | NCBI |  | CY024155 | NCBI |  | KF260144 | NCBI |
| JQ356879 | NCBI |  | GQ373106 | NCBI |  | GU474569 | NCBI |  | CY023772 | NCBI |
| CY087180 | NCBI |  | EU216093 | NCBI |  | KF259395 | NCBI |  | CY087183 | fludb |
| JF795047 | NCBI |  | CY063664 | NCBI |  | KM609631 | NCBI |  | FJ793336 | NCBI |
| JN653624 | NCBI |  | EU346938 | NCBI |  | KM609632 | NCBI |  | KF059316 | NCBI |
| CY087188 | NCBI |  | EU429726 | NCBI |  | KF259398 | NCBI |  | KF260130 | fludb |
| KC821157 | NCBI |  | JF519782 | NCBI |  | KF259369 | NCBI |  | KF260158 | NCBI |
| KC767260 | NCBI |  | JN653646 | NCBI |  | KF259370 | NCBI |  | KF260208 | NCBI |
| GQ373090 | NCBI |  | JF519783 | NCBI |  | JN869549 | NCBI |  | AF508723 | NCBI |
| KC821156 | NCBI |  | FJ793334 | NCBI |  | KM609633 | NCBI |  | KF059317 | NCBI |
| GQ373093 | NCBI |  | EF626681 | NCBI |  | KM609634 | NCBI |  | KF059318 | NCBI |
| GQ335499 | NCBI |  | EF626676 | NCBI |  | KF259439 | NCBI |  | KF746847 | NCBI |
| GQ373091 | NCBI |  | EU516313 | NCBI |  | KM609635 | NCBI |  | KF059319 | NCBI |
| KC821154 | NCBI |  | EF626683 | NCBI |  | KM609636 | NCBI |  | CY023708 | NCBI |
| KC821150 | NCBI |  | KC821219 | NCBI |  | EU835748 | NCBI |  | KF746752 | NCBI |
| JN653627 | fludb |  | HQ398343 | NCBI |  | KF259363 | NCBI |  | KF260129 | fludb |
| JN653635 | NCBI |  | CY023818 | NCBI |  | JN653668 | NCBI |  | FJ793448 | NCBI |
| CY087172 | NCBI |  | KF259504 | fludb |  | EF155000 | NCBI |  | KF059320 | NCBI |
| GU474607 | NCBI |  | KF259518 | fludb |  | KF259333 | NCBI |  | DQ997454 | NCBI |
| JN653637 | NCBI |  | KF259510 | NCBI |  | JN653666 | NCBI |  | AY664745 | fludb |
| GU474599 | NCBI |  | JQ770151 | NCBI |  | KM609637 | NCBI |  | KF260162 | NCBI |
| JF795103 | NCBI |  | HQ398344 | NCBI |  | KM609638 | NCBI |  | JN653684 | NCBI |
| KC779053 | NCBI |  | KF259545 | NCBI |  | KM609639 | NCBI |  | GU121386 | NCBI |
| KC821149 | NCBI |  | KF178671 | NCBI |  |  |  |  | KF059321 | NCBI |
| KC821160 | NCBI |  | KF259564 | NCBI |  |  |  |  | KF260134 | fludb |
| KF746820 | NCBI |  | KF259547 | NCBI |  |  |  |  | JN653673 | NCBI |
| JN653634 | fludb |  | KF259513 | NCBI |  |  |  |  | JF795148 | NCBI |
| JF795063 | NCBI |  | EU644488 | NCBI |  |  |  |  | JN653681 | fludb |
| HM998923 | NCBI |  | KF259505 | NCBI |  |  |  |  | AY664738 | NCBI |
| KC768050 | NCBI |  | KF178695 | NCBI |  |  |  |  | KC767263 | NCBI |
| HM998915 | NCBI |  | KC821220 | NCBI |  |  |  |  | KF260209 | NCBI |
| FJ581428 | NCBI |  | CY023842 | NCBI |  |  |  |  | KF059322 | NCBI |
| KC821162 | NCBI |  | KF259503 | NCBI |  |  |  |  | FJ793296 | NCBI |
| KC821167 | NCBI |  | HQ398346 | NCBI |  |  |  |  | GQ373148 | NCBI |
| JN653623 | NCBI |  | CY023826 | NCBI |  |  |  |  | CY023700 | NCBI |
| KC821165 | NCBI |  | KF259562 | NCBI |  |  |  |  | EU250012 | NCBI |
| KC821166 | NCBI |  | HQ398341 | NCBI |  |  |  |  | KF260122 | NCBI |
| KF746852 | NCBI |  | KF259546 | NCBI |  |  |  |  | KF260149 | NCBI |
| KC821173 | NCBI |  | KF178663 | NCBI |  |  |  |  | AY253756 | NCBI |
| KC779054 | NCBI |  | KF178687 | fludb |  |  |  |  | DQ226124 | NCBI |
| KC821170 | NCBI |  | KF259548 | NCBI |  |  |  |  | HM998918 | NCBI |
| KC821172 | NCBI |  | KF259542 | fludb |  |  |  |  | KF746814 | NCBI |
| KF259878 | NCBI |  | EU216106 | NCBI |  |  |  |  | HM751190 | NCBI |
| KC821174 | NCBI |  | KF259583 | NCBI |  |  |  |  | KF059323 | NCBI |
| KC821171 | NCBI |  | KF259517 | NCBI |  |  |  |  | EU250009 | NCBI |
| JF519795 | NCBI |  | JQ770152 | NCBI |  |  |  |  | HM590773 | fludb |
| JF519797 | NCBI |  | KF297306 | NCBI |  |  |  |  | EU086275 | fludb |
| KF259880 | NCBI |  | KF259549 | fludb |  |  |  |  | KC464602 | NCBI |
| KC821161 | NCBI |  | KC821221 | NCBI |  |  |  |  | FJ793312 | NCBI |
| JF795071 | NCBI |  | KF259591 | NCBI |  |  |  |  | KF260167 | NCBI |
| KC821164 | NCBI |  | KF367733 | NCBI |  |  |  |  | KM609720 | NCBI |
| KC821168 | NCBI |  | KC821222 | NCBI |  |  |  |  | KM609721 | NCBI |
| KC821163 | NCBI |  | KF259550 | NCBI |  |  |  |  | KF260166 | fludb |
| KF746803 | NCBI |  | JQ405084 | NCBI |  |  |  |  | GU474594 | NCBI |
| KC464599 | NCBI |  | KF259577 | NCBI |  |  |  |  | HM751198 | NCBI |
| KC821169 | NCBI |  | KF259581 | NCBI |  |  |  |  | KM609722 | NCBI |
| JF519796 | NCBI |  | KC767261 | NCBI |  |  |  |  | KM609723 | NCBI |
| KF059304 | NCBI |  | KF259590 | NCBI |  |  |  |  | GU474554 | NCBI |
| KF059305 | NCBI |  | KF259570 | fludb |  |  |  |  | AY664746 | NCBI |
| KF059306 | NCBI |  | KF259556 | NCBI |  |  |  |  | JF795066 | NCBI |
| KF367739 | NCBI |  | KF259541 | NCBI |  |  |  |  | EU086230 | NCBI |
| KF059307 | NCBI |  | JQ770145 | NCBI |  |  |  |  | GQ373137 | NCBI |
| KF059308 | NCBI |  | KC821224 | NCBI |  |  |  |  | KM609724 | NCBI |
| KF059309 | NCBI |  | KF259571 | NCBI |  |  |  |  | KM609725 | NCBI |
| KF059310 | NCBI |  | KF259561 | NCBI |  |  |  |  | KM609726 | NCBI |
| KC417055 | NCBI |  | KF259589 | NCBI |  |  |  |  | KM609727 | NCBI |
| KF059311 | NCBI |  | KC821225 | NCBI |  |  |  |  | JN653680 | NCBI |
| JF795087 | NCBI |  | KF259587 | NCBI |  |  |  |  | KM609728 | NCBI |
| KC768049 | NCBI |  | KF259555 | fludb |  |  |  |  | KF367742 | NCBI |
| KF059312 | NCBI |  | KF259586 | NCBI |  |  |  |  | KC951126 | NCBI |
| KF059313 | NCBI |  | KF259568 | NCBI |  |  |  |  | JN653670 | NCBI |
| KM609680 | NCBI |  | KC951124 | NCBI |  |  |  |  | JN653674 | NCBI |
| KF259890 | NCBI |  | KF259551 | NCBI |  |  |  |  | GQ202058 | NCBI |
| KF259904 | NCBI |  | KF297304 | NCBI |  |  |  |  | JN653678 | NCBI |
| KF259918 | NCBI |  | KF297308 | fludb |  |  |  |  | KM609729 | NCBI |
| KF259909 | NCBI |  | KF259566 | NCBI |  |  |  |  | KF260140 | NCBI |
| KF259907 | NCBI |  | KF259554 | fludb |  |  |  |  | KM609730 | NCBI |
| KF259903 | NCBI |  | KF259575 | NCBI |  |  |  |  | DQ997423 | NCBI |
| KF259884 | NCBI |  | KF259552 | NCBI |  |  |  |  | KF260147 | NCBI |
| KF259922 | NCBI |  | KF259560 | NCBI |  |  |  |  | KM609731 | NCBI |
| KF259901 | NCBI |  | KF259529 | NCBI |  |  |  |  | KF260123 | NCBI |
| KF259892 | NCBI |  | KF259553 | NCBI |  |  |  |  | KM609732 | NCBI |
| KF259920 | NCBI |  | KF259558 | NCBI |  |  |  |  | KF260133 | NCBI |
| KF259924 | NCBI |  | KF259557 | NCBI |  |  |  |  | EU086255 | fludb |
| KF259900 | NCBI |  | KF259595 | fludb |  |  |  |  | GQ373138 | NCBI |
| KF259906 | fludb |  | KF259572 | fludb |  |  |  |  | KM609733 | NCBI |
| KM609682 | NCBI |  | KF259569 | NCBI |  |  |  |  | KF715232 | NCBI |
| KF259917 | NCBI |  | KF259573 | fludb |  |  |  |  | JF795082 | NCBI |
| KF259910 | NCBI |  | KF259580 | fludb |  |  |  |  | KM609734 | NCBI |
| KF259879 | NCBI |  | KF259593 | fludb |  |  |  |  | KF260216 | NCBI |
| KF259915 | NCBI |  | KF297307 | NCBI |  |  |  |  | CY146664 | NCBI |
| KF259885 | fludb |  | KF500979 | NCBI |  |  |  |  | CY024060 | NCBI |
| KF259913 | NCBI |  | KF178679 | NCBI |  |  |  |  | EU086311 | NCBI |
| KF259889 | NCBI |  | KF259596 | fludb |  |  |  |  | HM590766 | fludb |
| JN869547 | NCBI |  | KF259579 | NCBI |  |  |  |  | KM609735 | NCBI |
| KC951123 | NCBI |  | KF259594 | fludb |  |  |  |  | KM609736 | NCBI |
| KF259912 | NCBI |  | KC821233 | NCBI |  |  |  |  | KM609737 | NCBI |
| KF259919 | NCBI |  | KC821229 | NCBI |  |  |  |  | EU250010 | NCBI |
| KF259908 | fludb |  | KF297309 | NCBI |  |  |  |  | KM609738 | NCBI |
| KF259921 | NCBI |  | KC821223 | NCBI |  |  |  |  | KF746839 | NCBI |
| KF259886 | fludb |  | KC821234 | NCBI |  |  |  |  | EU086291 | NCBI |
| KF259911 | fludb |  | KC821231 | NCBI |  |  |  |  | KF260165 | NCBI |
| KF259905 | NCBI |  | KF259576 | NCBI |  |  |  |  | KM609739 | NCBI |
| KF259916 | NCBI |  | KF259563 | NCBI |  |  |  |  | KM609740 | NCBI |
| KF259895 | NCBI |  | KF259592 | fludb |  |  |  |  | KF260214 | fludb |
| KF259894 | NCBI |  | KJ128364 | NCBI |  |  |  |  | KF715248 | NCBI |
| JN869524 | NCBI |  | KF259565 | NCBI |  |  |  |  | GQ202050 | NCBI |
| KF259923 | NCBI |  | KF259574 | NCBI |  |  |  |  | KM609741 | NCBI |
| KM609683 | NCBI |  | KF259578 | fludb |  |  |  |  | KM609742 | NCBI |
| KC417056 | NCBI |  | KF715230 | NCBI |  |  |  |  | KM609743 | NCBI |
| JQ356880 | NCBI |  | HM998924 | NCBI |  |  |  |  | JF795090 | NCBI |
| KM609684 | NCBI |  | KF746783 | NCBI |  |  |  |  | JN653682 | NCBI |
| KF259974 | NCBI |  | JF519776 | NCBI |  |  |  |  | KF260213 | fludb |
| KM609685 | NCBI |  | JF795048 | NCBI |  |  |  |  | KM609744 | NCBI |
| JN653632 | NCBI |  | JN683653 | NCBI |  |  |  |  | KF260148 | NCBI |
| KF259959 | NCBI |  | JN683651 | NCBI |  |  |  |  | KM609745 | NCBI |
| KF259956 | NCBI |  | KC821230 | NCBI |  |  |  |  | DQ997431 | NCBI |
| KM609686 | NCBI |  | FJ581434 | NCBI |  |  |  |  | EU086310 | fludb |
| JN869539 | NCBI |  | GQ373105 | NCBI |  |  |  |  | DQ226127 | NCBI |
| JN869531 | NCBI |  | KF746771 | NCBI |  |  |  |  | AY664736 | NCBI |
| KM609687 | NCBI |  | KF715238 | NCBI |  |  |  |  | AY664749 | fludb |
| CY146661 | NCBI |  | KC821228 | NCBI |  |  |  |  | HM751174 | NCBI |
| KM609688 | NCBI |  | KF746861 | NCBI |  |  |  |  | DQ981630 | NCBI |
| KF259955 | NCBI |  | JF519777 | NCBI |  |  |  |  | GU474578 | NCBI |
| JX312545 | NCBI |  | CY087173 | NCBI |  |  |  |  | KM609746 | NCBI |
| JQ356878 | NCBI |  | KF746750* | fludb |  |  |  |  | EU516307 | NCBI |
| KF259914 | NCBI |  | EU346933 | NCBI |  |  |  |  | KM609747 | NCBI |
| KM609689 | NCBI |  | HQ185385 | NCBI |  |  |  |  | KF260157 | fludb |
| KF715245 | NCBI |  | GU474600 | NCBI |  |  |  |  | FJ499465 | NCBI |
| KM609690 | NCBI |  | EU939162 | NCBI |  |  |  |  | FJ547485 | NCBI |
| KF259893 | NCBI |  | KC821227 | NCBI |  |  |  |  | KF178691 | NCBI |
| KF259960 | NCBI |  | JF519784 | NCBI |  |  |  |  | KM609748 | NCBI |
| KM609691 | NCBI |  | CY087189 | NCBI |  |  |  |  | KM609749 | NCBI |
| KF178681 | NCBI |  | GQ373110 | NCBI |  |  |  |  | JF795050 | NCBI |
| JN653629 | NCBI |  | EU340028 | NCBI |  |  |  |  | KF715240 | NCBI |
| KM609691 | NCBI |  | HM751156 | NCBI |  |  |  |  | KM609750 | NCBI |
| KM609692 | NCBI |  | GU474608 | NCBI |  |  |  |  | CY023828 | NCBI |
| KM609693 | NCBI |  | KF746845 | NCBI |  |  |  |  | JF795042 | NCBI |
| KC417057 | NCBI |  | FJ492964 | NCBI |  |  |  |  | KM609751 | NCBI |
| KM609694 | NCBI |  | JF519781 | NCBI |  |  |  |  | FJ793304 | NCBI |
| KF259972 | NCBI |  | KC768045 | NCBI |  |  |  |  | JN869527 | NCBI |
| KM609695 | NCBI |  | GQ335500 | NCBI |  |  |  |  | DQ064487 | NCBI |
| KM609696 | NCBI |  | KC821226 | NCBI |  |  |  |  | KM609752 | NCBI |
| KM609697 | NCBI |  | KC821232 | NCBI |  |  |  |  | KM609753 | NCBI |
| KM609698 | NCBI |  | HM751188 | NCBI |  |  |  |  | KF260159 | NCBI |
| KM609699 | NCBI |  | CY087181 | NCBI |  |  |  |  | EU753342 | fludb |
| KM609700 | NCBI |  | JF795080 | NCBI |  |  |  |  | KC768051 | NCBI |
| KM609701 | NCBI |  | JN683650 | NCBI |  |  |  |  | GQ373139 | NCBI |
| KF259976 | NCBI |  | JF519780 | NCBI |  |  |  |  | GU474570 | NCBI |
| KM609702 | NCBI |  | GQ335508 | NCBI |  |  |  |  | KF260211 | NCBI |
| KM609703 | NCBI |  | JF519778 | NCBI |  |  |  |  | JN869542 | NCBI |
| KM609704 | NCBI |  | GQ335481 | NCBI |  |  |  |  | KF260200 | NCBI |
| KM609705 | NCBI |  | KF746821 | NCBI |  |  |  |  | FJ793328 | NCBI |
| KF259965 | NCBI |  | JF519779 | NCBI |  |  |  |  | GU474610 | NCBI |
| KM609706 | NCBI |  | KF367740 | NCBI |  |  |  |  | KM609754 | NCBI |
| KF259971 | fludb |  | GU474584 | NCBI |  |  |  |  | KF714779 | NCBI |
| KF259975 | NCBI |  | KF259514 | NCBI |  |  |  |  | KM609755 | NCBI |
| KJ128365 | NCBI |  | JF795104 | NCBI |  |  |  |  | KM609756 | NCBI |
| KM609707 | NCBI |  | GQ373108 | NCBI |  |  |  |  | KM609757 | NCBI |
| KM609708 | NCBI |  | GU474552 | NCBI |  |  |  |  | KM609758 | NCBI |
| KM609709 | NCBI |  | JF519786 | NCBI |  |  |  |  | KM609759 | NCBI |
| KF259957 | NCBI |  | JN653647 | NCBI |  |  |  |  |  |  |
| KM609710 | NCBI |  | KC768046 | NCBI |  |  |  |  |  |  |
| KM609711 | NCBI |  | KC821238 | NCBI |  |  |  |  |  |  |
| KM609712 | NCBI |  | JN653644 | NCBI |  |  |  |  |  |  |
| KF500980 | NCBI |  | JF795040 | NCBI |  |  |  |  |  |  |
| KM609713 | NCBI |  | JN653651 | NCBI |  |  |  |  |  |  |
| KM609714 | NCBI |  | KC821248 | NCBI |  |  |  |  |  |  |
| KM609715 | NCBI |  | JF519785 | NCBI |  |  |  |  |  |  |
| KF259958 | NCBI |  | JF795096 | NCBI |  |  |  |  |  |  |
| KF178665 | fludb |  | GQ373118 | NCBI |  |  |  |  |  |  |
| KM609716 | NCBI |  | KC768047 | NCBI |  |  |  |  |  |  |
| KM609717 | NCBI |  | HM998916 | NCBI |  |  |  |  |  |  |
| KM609718 | NCBI |  | HM751196 | NCBI |  |  |  |  |  |  |
| KM609719 | NCBI |  | JN653649 | NCBI |  |  |  |  |  |  |
|  |  |  | HQ378728 | NCBI |  |  |  |  |  |  |
|  |  |  | JF795056 | NCBI |  |  |  |  |  |  |
|  |  |  | KC821244 | NCBI |  |  |  |  |  |  |
|  |  |  | KC779051 | NCBI |  |  |  |  |  |  |
|  |  |  | JN653650 | NCBI |  |  |  |  |  |  |
|  |  |  | GU474592 | NCBI |  |  |  |  |  |  |
|  |  |  | JF795146 | NCBI |  |  |  |  |  |  |
|  |  |  | JN653642 | NCBI |  |  |  |  |  |  |
|  |  |  | GQ335492 | NCBI |  |  |  |  |  |  |
|  |  |  | GU474568 | NCBI |  |  |  |  |  |  |
|  |  |  | GU474576 | NCBI |  |  |  |  |  |  |
|  |  |  | JN653652 | NCBI |  |  |  |  |  |  |
|  |  |  | JN653638 | NCBI |  |  |  |  |  |  |
|  |  |  | KC821245 | NCBI |  |  |  |  |  |  |
|  |  |  | JN653639 | NCBI |  |  |  |  |  |  |
|  |  |  | JN653641 | NCBI |  |  |  |  |  |  |
|  |  |  | KF746804 | NCBI |  |  |  |  |  |  |
|  |  |  | JX312544 | NCBI |  |  |  |  |  |  |
|  |  |  | KF259543 | NCBI |  |  |  |  |  |  |
|  |  |  | KC821236 | NCBI |  |  |  |  |  |  |
|  |  |  | KF714777 | NCBI |  |  |  |  |  |  |
|  |  |  | KF259539 | NCBI |  |  |  |  |  |  |
|  |  |  | JN653648 | NCBI |  |  |  |  |  |  |
|  |  |  | KF259515 | NCBI |  |  |  |  |  |  |
|  |  |  | JQ770150 | NCBI |  |  |  |  |  |  |
|  |  |  | KF259536 | NCBI |  |  |  |  |  |  |
|  |  |  | GQ335516 | NCBI |  |  |  |  |  |  |
|  |  |  | KF259528 | NCBI |  |  |  |  |  |  |
|  |  |  | KF259531 | fludb |  |  |  |  |  |  |
|  |  |  | KC821241 | NCBI |  |  |  |  |  |  |
|  |  |  | KF259540 | NCBI |  |  |  |  |  |  |
|  |  |  | KC821242 | NCBI |  |  |  |  |  |  |
|  |  |  | JQ770149 | NCBI |  |  |  |  |  |  |
|  |  |  | KC821246 | NCBI |  |  |  |  |  |  |
|  |  |  | JN869532 | NCBI |  |  |  |  |  |  |
|  |  |  | KC821237 | NCBI |  |  |  |  |  |  |
|  |  |  | KF259544 | NCBI |  |  |  |  |  |  |
|  |  |  | JQ770154 | NCBI |  |  |  |  |  |  |
|  |  |  | JN653643 | NCBI |  |  |  |  |  |  |
|  |  |  | KF259530 | NCBI |  |  |  |  |  |  |
|  |  |  | KF188362 | NCBI |  |  |  |  |  |  |
|  |  |  | KF259526 | NCBI |  |  |  |  |  |  |
|  |  |  | KC821235 | NCBI |  |  |  |  |  |  |
|  |  |  | JF906208 | NCBI |  |  |  |  |  |  |
|  |  |  | KF188402 | NCBI |  |  |  |  |  |  |
|  |  |  | KF714785 | NCBI |  |  |  |  |  |  |
|  |  |  | JN869548 | NCBI |  |  |  |  |  |  |
|  |  |  | JQ770163 | NCBI |  |  |  |  |  |  |
|  |  |  | JN653640 | NCBI |  |  |  |  |  |  |
|  |  |  | KC821240 | NCBI |  |  |  |  |  |  |
|  |  |  | KF259522 | fludb |  |  |  |  |  |  |
|  |  |  | KC821243 | NCBI |  |  |  |  |  |  |
|  |  |  | JN653645 | NCBI |  |  |  |  |  |  |
|  |  |  | JN869540 | NCBI |  |  |  |  |  |  |
|  |  |  | JQ770146 | NCBI |  |  |  |  |  |  |
|  |  |  | HQ225838 | NCBI |  |  |  |  |  |  |
|  |  |  | KF259520 | NCBI |  |  |  |  |  |  |
|  |  |  | JF795064 | NCBI |  |  |  |  |  |  |
|  |  |  | JF795072 | NCBI |  |  |  |  |  |  |
|  |  |  | KC821239 | NCBI |  |  |  |  |  |  |
|  |  |  | HM036347 | NCBI |  |  |  |  |  |  |
|  |  |  | KC821247 | NCBI |  |  |  |  |  |  |
|  |  |  | JN869525 | NCBI |  |  |  |  |  |  |
|  |  |  | KF746853 | NCBI |  |  |  |  |  |  |
|  |  |  | KF059294 | NCBI |  |  |  |  |  |  |
|  |  |  | KF059295 | NCBI |  |  |  |  |  |  |
|  |  |  | JQ770162 | NCBI |  |  |  |  |  |  |
|  |  |  | KF059296 | NCBI |  |  |  |  |  |  |
|  |  |  | JQ356877 | NCBI |  |  |  |  |  |  |
|  |  |  | JN683649 | NCBI |  |  |  |  |  |  |
|  |  |  | KC779052 | NCBI |  |  |  |  |  |  |
|  |  |  | JF795088 | NCBI |  |  |  |  |  |  |
|  |  |  | JQ770158 | NCBI |  |  |  |  |  |  |
|  |  |  | KF059297 | NCBI |  |  |  |  |  |  |
|  |  |  | JN653653 | NCBI |  |  |  |  |  |  |
|  |  |  | KF059298 | NCBI |  |  |  |  |  |  |
|  |  |  | KF259516 | NCBI |  |  |  |  |  |  |
|  |  |  | JN683648 | NCBI |  |  |  |  |  |  |
|  |  |  | JQ770161 | NCBI |  |  |  |  |  |  |
|  |  |  | KF059299 | NCBI |  |  |  |  |  |  |
|  |  |  | KF259521 | fludb |  |  |  |  |  |  |
|  |  |  | KC608159 | NCBI |  |  |  |  |  |  |
|  |  |  | KF059300 | NCBI |  |  |  |  |  |  |
|  |  |  | KF059301 | NCBI |  |  |  |  |  |  |
|  |  |  | KF059302 | NCBI |  |  |  |  |  |  |
|  |  |  | KF059303 | NCBI |  |  |  |  |  |  |
|  |  |  | KM609640 | NCBI |  |  |  |  |  |  |
|  |  |  | JQ770147 | NCBI |  |  |  |  |  |  |
|  |  |  | JQ770159 | NCBI |  |  |  |  |  |  |
|  |  |  | KM609641 | NCBI |  |  |  |  |  |  |
|  |  |  | KM609642 | NCBI |  |  |  |  |  |  |
|  |  |  | JQ770156 | NCBI |  |  |  |  |  |  |
|  |  |  | KM609643 | NCBI |  |  |  |  |  |  |
|  |  |  | KC417052 | NCBI |  |  |  |  |  |  |
|  |  |  | KM609644 | NCBI |  |  |  |  |  |  |
|  |  |  | JQ356876 | NCBI |  |  |  |  |  |  |
|  |  |  | KM609645 | NCBI |  |  |  |  |  |  |
|  |  |  | KC417054 | NCBI |  |  |  |  |  |  |
|  |  |  | JQ770157 | fludb |  |  |  |  |  |  |
|  |  |  | JQ356875 | NCBI |  |  |  |  |  |  |
|  |  |  | KF715246 | NCBI |  |  |  |  |  |  |
|  |  |  | JQ770155 | NCBI |  |  |  |  |  |  |
|  |  |  | KC417053 | NCBI |  |  |  |  |  |  |
|  |  |  | JQ770160 | NCBI |  |  |  |  |  |  |
|  |  |  | JQ770148 | NCBI |  |  |  |  |  |  |
|  |  |  | KM609646 | NCBI |  |  |  |  |  |  |
|  |  |  | JQ770153 | NCBI |  |  |  |  |  |  |
|  |  |  | JQ409042 | NCBI |  |  |  |  |  |  |
|  |  |  | JQ770144 | NCBI |  |  |  |  |  |  |
|  |  |  | KM609647 | NCBI |  |  |  |  |  |  |
|  |  |  | CY146662 | NCBI |  |  |  |  |  |  |
|  |  |  | KM609648 | NCBI |  |  |  |  |  |  |
|  |  |  | KM609649 | NCBI |  |  |  |  |  |  |
|  |  |  | KM609650 | NCBI |  |  |  |  |  |  |
|  |  |  | KM609651 | NCBI |  |  |  |  |  |  |
|  |  |  | KM609652 | NCBI |  |  |  |  |  |  |
|  |  |  | KM609653 | NCBI |  |  |  |  |  |  |
|  |  |  | KM609654 | NCBI |  |  |  |  |  |  |
|  |  |  | KM609655 | NCBI |  |  |  |  |  |  |
|  |  |  | KM609656 | NCBI |  |  |  |  |  |  |
|  |  |  | KM609657 | NCBI |  |  |  |  |  |  |
|  |  |  | KM609658 | NCBI |  |  |  |  |  |  |
|  |  |  | KM609659 | NCBI |  |  |  |  |  |  |
|  |  |  | KM609660 | NCBI |  |  |  |  |  |  |
|  |  |  | KM609661 | NCBI |  |  |  |  |  |  |
|  |  |  | KC464600 | NCBI |  |  |  |  |  |  |
|  |  |  | KM609662 | NCBI |  |  |  |  |  |  |
|  |  |  | KM609663 | NCBI |  |  |  |  |  |  |
|  |  |  | KM609664 | NCBI |  |  |  |  |  |  |
|  |  |  | KM609665 | NCBI |  |  |  |  |  |  |
|  |  |  | KM609666 | NCBI |  |  |  |  |  |  |
|  |  |  | KM609667 | NCBI |  |  |  |  |  |  |
|  |  |  | KM609668 | NCBI |  |  |  |  |  |  |
|  |  |  | KM609669 | NCBI |  |  |  |  |  |  |
|  |  |  | KM609670 | NCBI |  |  |  |  |  |  |
|  |  |  | KM609671 | NCBI |  |  |  |  |  |  |
|  |  |  | KM609672 | NCBI |  |  |  |  |  |  |
|  |  |  | KM609673 | NCBI |  |  |  |  |  |  |
|  |  |  | KM609674 | NCBI |  |  |  |  |  |  |
|  |  |  | KM609675 | NCBI |  |  |  |  |  |  |
|  |  |  | KM609676 | NCBI |  |  |  |  |  |  |
|  |  |  | KM609677 | NCBI |  |  |  |  |  |  |
|  |  |  | KM609678 | NCBI |  |  |  |  |  |  |
|  |  |  | KM609679 | NCBI |  |  |  |  |  |  |
