# Supplementary material for: Genetic evolution of influenza H9N2 viruses isolated from various hosts in China from 1994 to 2013
Source: Emerg Microbes Infect. 2017 Nov 29;6(11):e106–. doi: 10.1038/emi.2017.94 (PMC5717095; doi:10.1038/emi.2017.94)
Supplement: Supplementary Table S1 [file emi201794x3.pdf]

Supplementary Figure S1

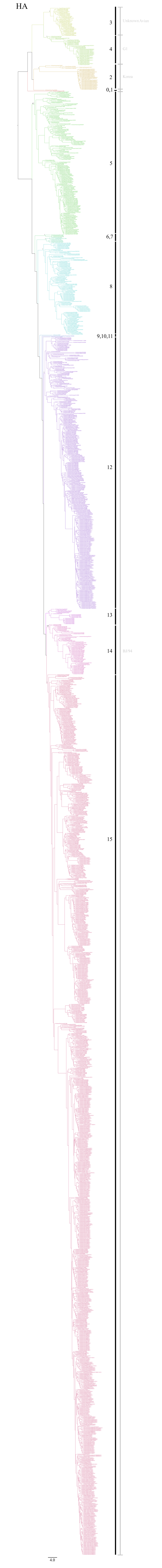

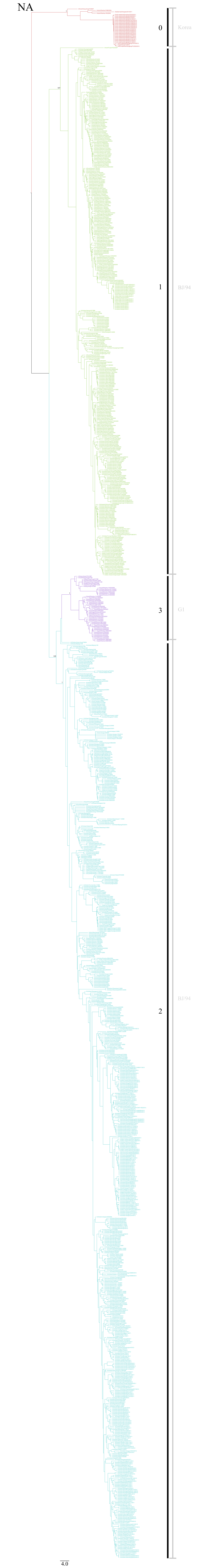
NA
0
Korea
1
BJ/94
3
G1
2
BJ/94
4.0

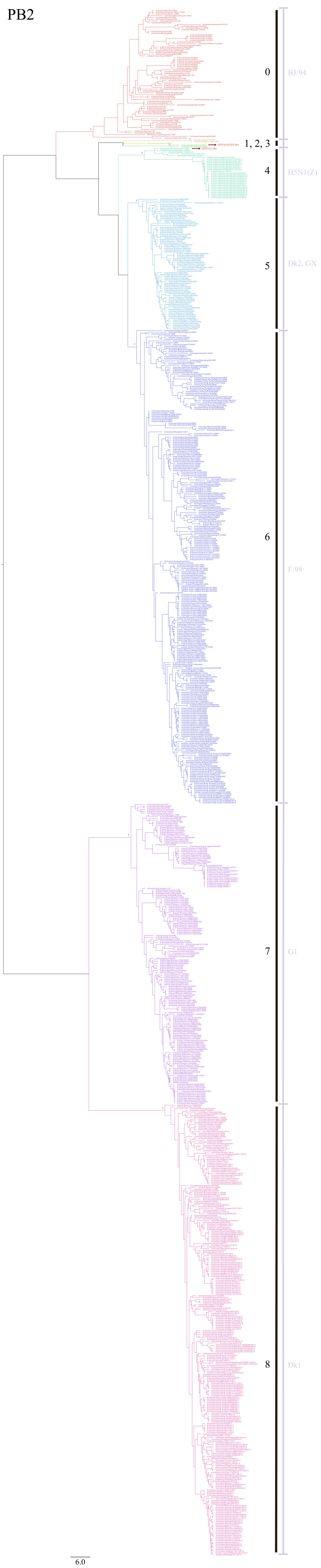
PB2
0
BJ/94
1, 2, 3
4
H5N1(Z)
5
Dk2, GX
6
F/98
7
G1
8
Dk1
6.0

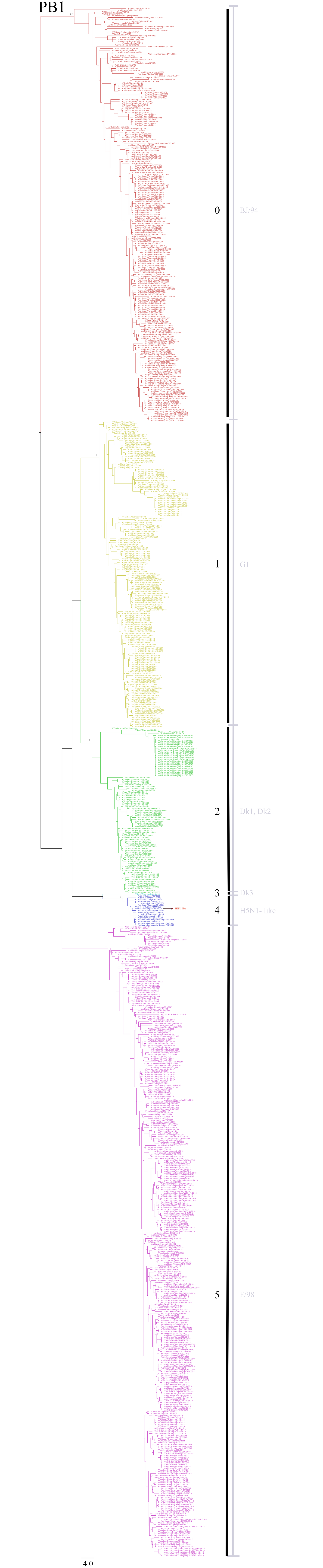
PB1
0
BJ/94
1
G1
2
Dk1, Dk2
3
Dk3
4
H5N1- like
5
F/98
4.0

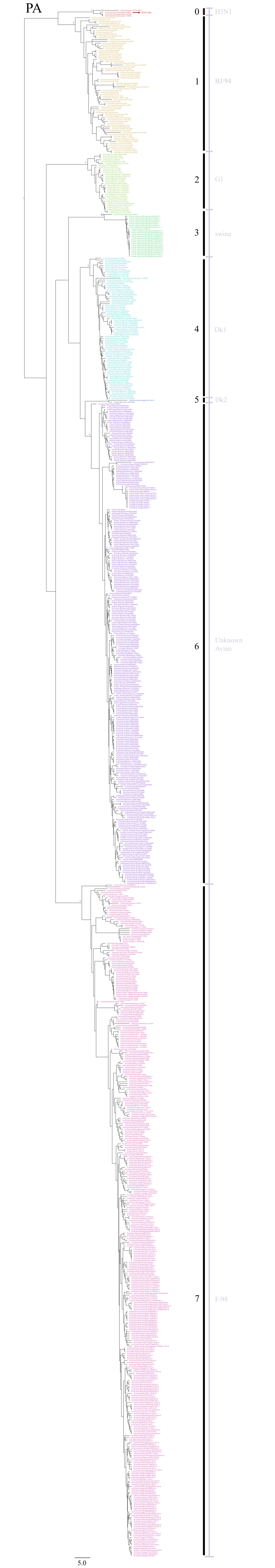
PA
0
H5N1
1
BJ/94
2
G1
3
swine
4
Dk1
5
Dk2
6
Unknown
Avian
7
F/98
5.0

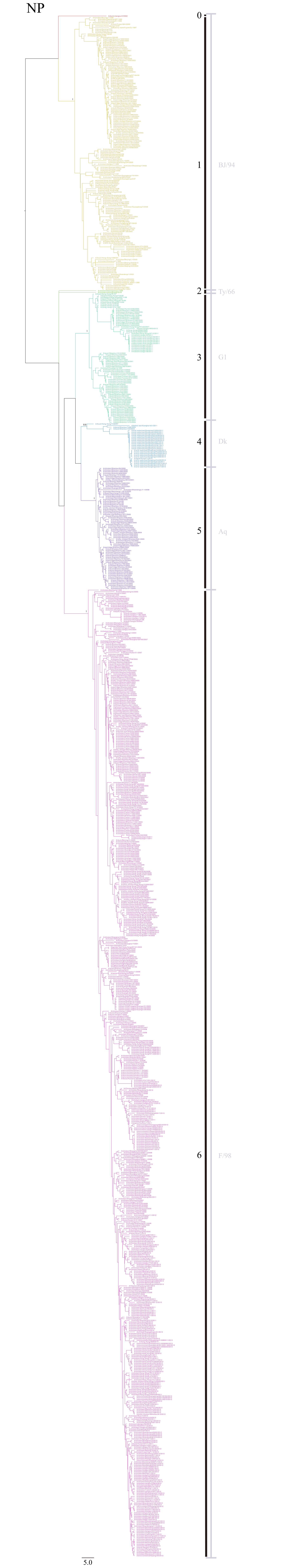
NP
0
1
BJ/94
2
Ty/66
3
G1
4
Dk
5
Aq
6
F/98
5.0

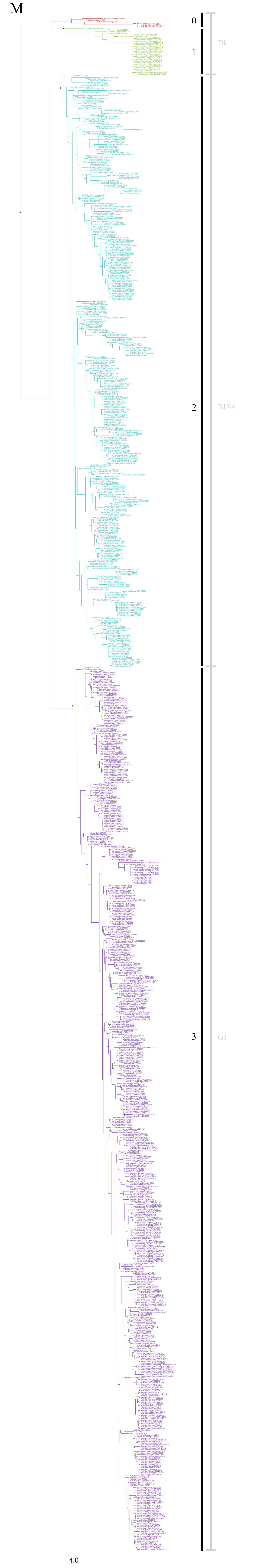
M
0
1
Dk
2
BJ/94
3
G1
4.0

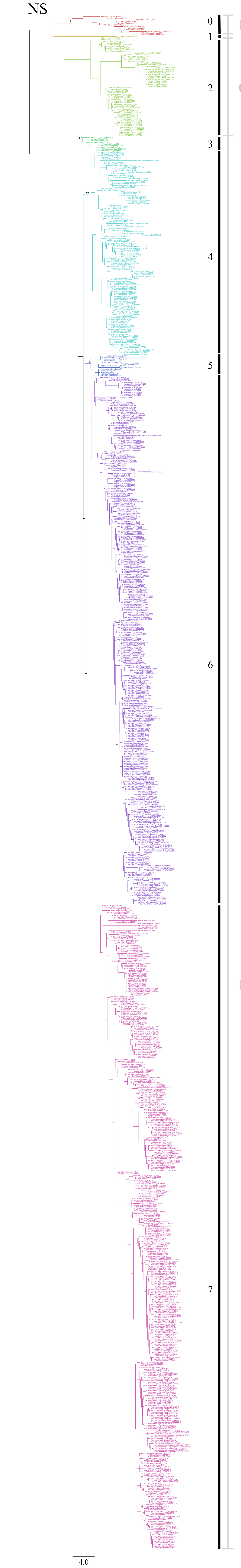

Supplementary Figure S1 Phylogenies of the HA, NA, PB2, PA, NP, M, and NS genes. The eight phylogenetic trees include all H9N2viruses isolated in China from 1994 to 2013. Posterior values are shown for selected clades/lineages. Node branch colors indicate specific clades. Vertical black lines with numbers indicate the previous clade classification. Arrows mark the strains with a close relation with the H5-subtype influenza virus described in previous studies.
